# Supplementary material for: PHF6-mediated transcriptional control of NSC via Ephrin receptors is impaired in the intellectual disability syndrome BFLS
Source: EMBO Rep. 2024 Mar 1;25(3):20. doi: 10.1038/s44319-024-00082-0 (PMC10933485; doi:10.1038/s44319-024-00082-0)
Supplement: Supplementary file 1 — Table EV1 [file 44319_2024_82_MOESM1_ESM.docx]

Expanded View Table EV1

| **RT-qPCR Primers** | **Sequence 5’**🡺**3’** |
| --- | --- |
| mEphA4-F | AGCAACTTGGTCTGCAAGGT |
| mEphA4-R | CTCCAGACATCACTGGCTGA |
| mEphA7-F | CAGAAAGATCGGGCGGAAAG |
| mEphA7-R | AAGGCAGTGAAGTCAGGAGT |
| mEphB1-F | CCAACATCATTCGCCTGGAG |
| mEphB1-R | GGTCCCGGTGCACATAATTC |
| mEphB2-F | ATCGTCATGTGGGAGGTGAT |
| mEphB2-R | TGGGCGGAGGTAGTCTGTAG |
| PHF6-F | TGAAATATGAGCTGGTCAATCAC |
| PHF6-R | TACAGTATTTTGGGGAAGCTGG |
| **Genotyping PCR**  **Primers** | **Sequence 5’**🡺**3’** |
| PHF6(C99F)-F | CAGTTGTATCTAGCTCAGCTC |
| PHF6(C99F)-R-wt | TGGTAGTGGTATGTCCTGTGGC |
| PHF6(C99F)-R-m | TGGTAGTGGTATGTCCTGTGGA |
| PHF6 R342X F | GCATGGTGTACAAGTGGAGATC |
| PHF6 R342X R-wt | CCACGGCTTTTACTCTCTCG |
| PHF6 R342X R-mutant | CCACGGCTTTTACTCTCTCA |
| PHF6(LoxP)-F | TGAAATATGAGCTGGTCAATCAC |
| PHF6(LoxP)-R | TACAGTATTTTGGGGAAGCTGG |
| Nestin-CreERT2 (oIMR1084) | GCGGTCTGGCAGTAAAAACTATC |
| Nestin-CreERT2  (oIMR1085) | GTGAAACAGCATTGCTGTCACTT |
| Nestin-CreERT2  (oIMR7338) | CTAGGCCACAGAATTGAAAGATCT |
| Nestin-CreERT2  (oIMR7339) | GTAGGTGGAAATTCTAGCATCATCC |
| Nestin-Cre-F | ATGCTTCTGTCCGTTTGCCG |
| Nestin-Cre-R | CCTGTTTTGCACGTTCACCG |
| **Luciferase Primers** | **Sequence 5’**🡺**3’** |
| EphA4-F | TAATCTCGGTACCCCCTACCCCAGATCCTTAGC |
| EphA4-R | TAATTCCGATATCGGATGTACGGAGTGGGAAGA RC(ATCTATCCATCCAGCCAGCC) |
| EphA7-F | TAATCTCGGTACCCGTTGATTGGCTCCTGCT |
| EphA7-R | TAATTCCGATATCTCAGAACAAACTTTGCTTTCCTC  RC(GAGGAAAGCAAAGTTTGTTCTGA) |
| EphB1-F | TAATCTCGGTACCCTTCCTAACCCTCCCACACA |
| EphB1-R | TAATTCCGATATCTCTCTCTCCAGCACCAGGAT RC(ATCCTGGTGCTGGAGAGAGA) |
| **ChIP-qPCR Primers** | **Sequence 5’**🡺**3’** |
| EphA4-F | GGAGGGGGAGAGAGACAGAC |
| EphA4-R | TTTTTCTGTCCGAGGTGAGG |
| EphA7-F | AAACGTGCCTCTGAGCTGAT |
| EphA7-R | CGGTTTCTGGTCACCAAAGT |
| EphB1-F | CAGGGAGAAAACCAAAGCAA |
| EphB1-R | CCCGTTTCTTCTCACACTCC |
| ZFP735-F | TGGTCCATCCTTTTGACACA |
| ZFP735-R | ACTTTGCCCCTTCGAATTTT |

**Table EV1:** Primer sequences from 5’ to 3’, forward and reverse, are listed for all genes assayed in RT-qPCR analysis, genotyping PCRs, luciferase construct design, and ChIP-qPCR.
