# Supplementary figures and images for: PHF6-mediated transcriptional control of NSC via Ephrin receptors is impaired in the intellectual disability syndrome BFLS (part 1 of 2)
Source: EMBO Rep. 2024 Mar 1;25(3):20. doi: 10.1038/s44319-024-00082-0 (PMC10933485; doi:10.1038/s44319-024-00082-0)

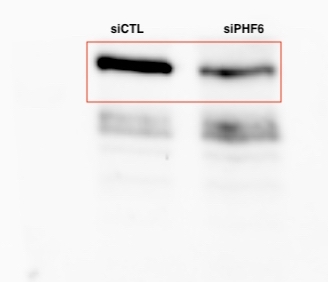

Supplement: Supplementary file 8 — Source Data Fig. 3 [file 44319_2024_82_MOESM8_ESM.zip › Figure 3/3C/western PHF6.jpg]

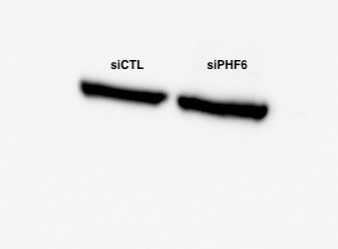

Supplement: Supplementary file 8 — Source Data Fig. 3 [file 44319_2024_82_MOESM8_ESM.zip › Figure 3/3C/western TUBULIN.jpg]

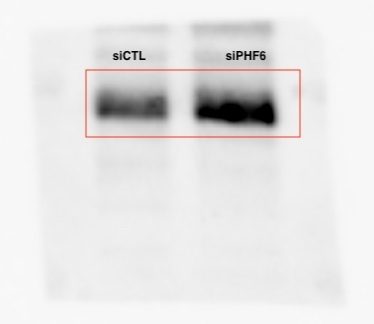

Supplement: Supplementary file 8 — Source Data Fig. 3 [file 44319_2024_82_MOESM8_ESM.zip › Figure 3/3C/western SOX2.jpg]

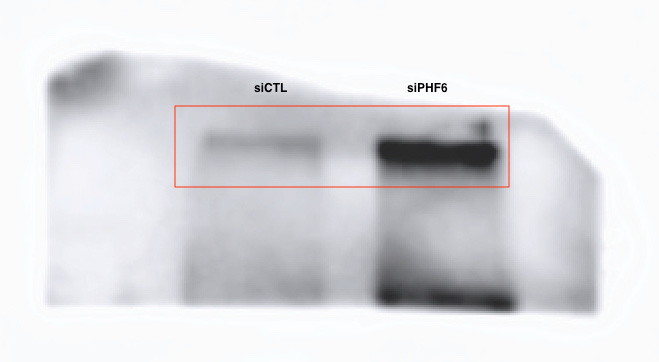

Supplement: Supplementary file 8 — Source Data Fig. 3 [file 44319_2024_82_MOESM8_ESM.zip › Figure 3/3C/western NESTIN.jpg]

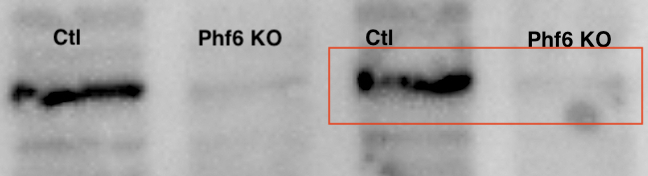

Supplement: Supplementary file 8 — Source Data Fig. 3 [file 44319_2024_82_MOESM8_ESM.zip › Figure 3/3D/western PHF6.png]

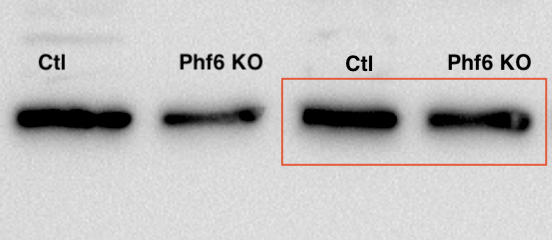

Supplement: Supplementary file 8 — Source Data Fig. 3 [file 44319_2024_82_MOESM8_ESM.zip › Figure 3/3D/western GAPDH.png]

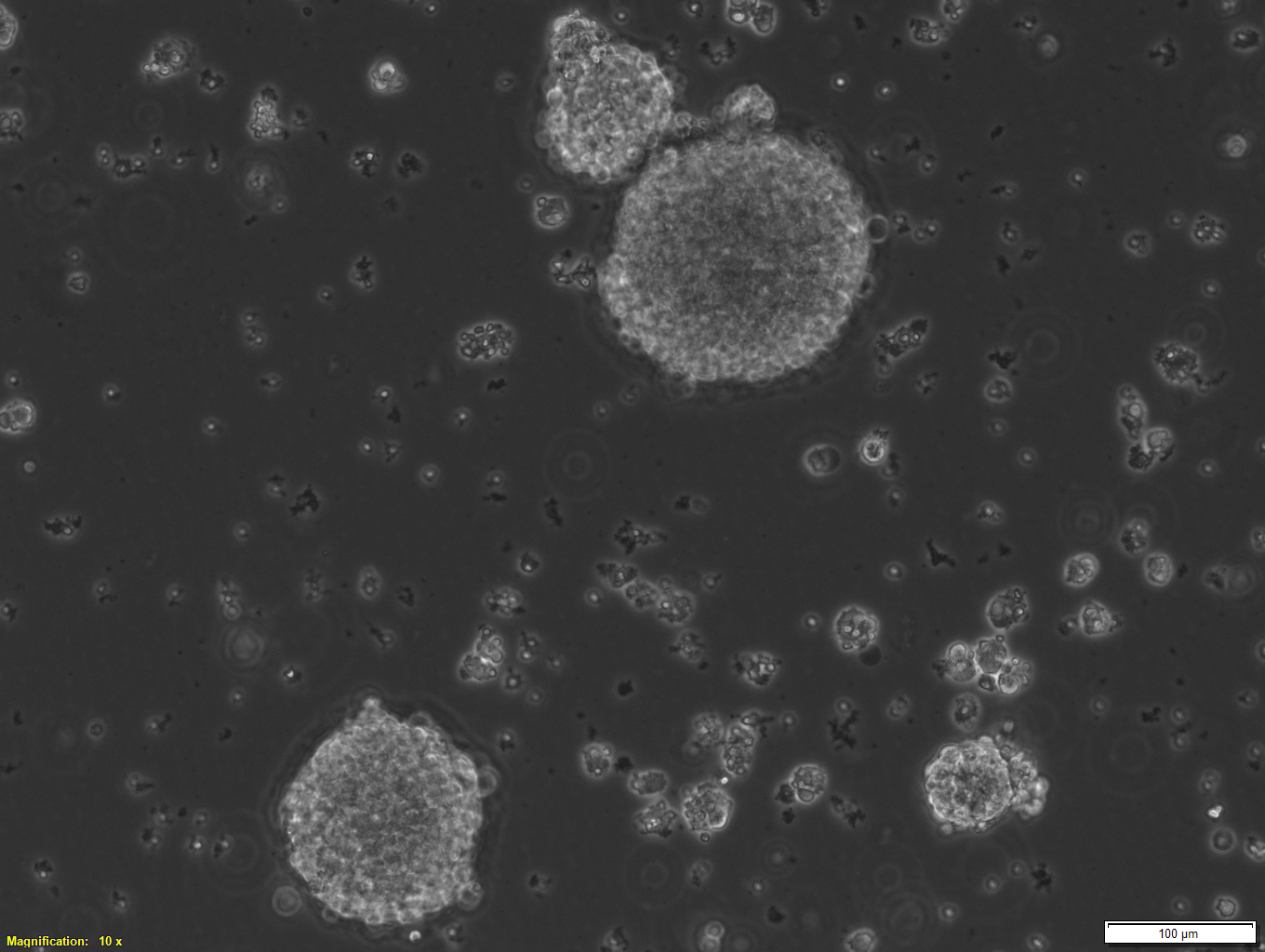

Supplement: Supplementary file 8 — Source Data Fig. 3 [file 44319_2024_82_MOESM8_ESM.zip › Figure 3/3M/3M Wild-type.tiff]

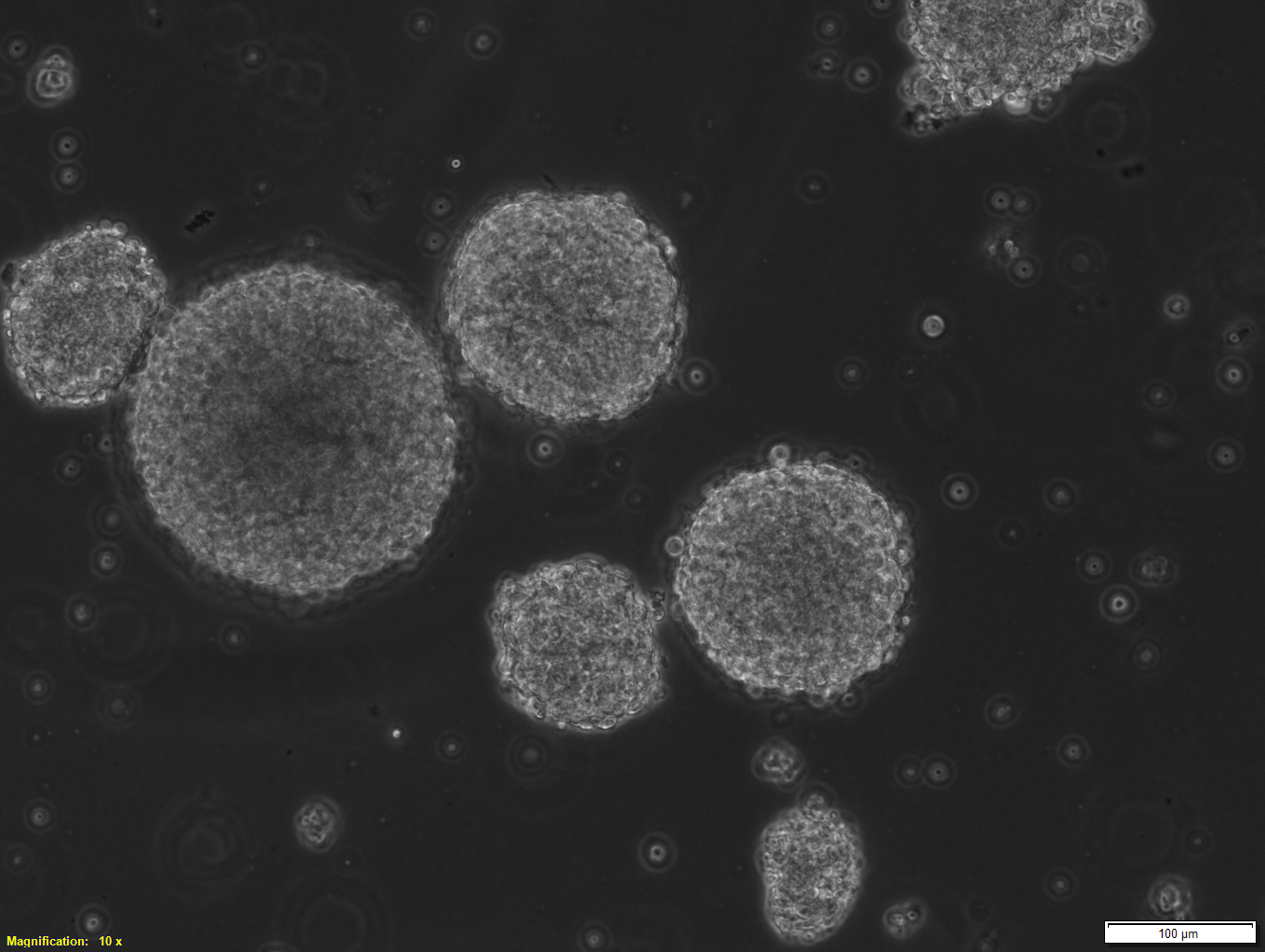

Supplement: Supplementary file 8 — Source Data Fig. 3 [file 44319_2024_82_MOESM8_ESM.zip › Figure 3/3M/3M R342X.tiff]

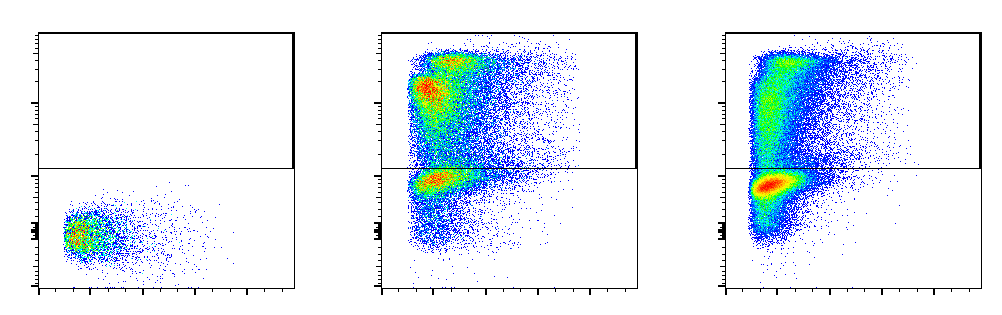

Supplement: Supplementary file 8 — Source Data Fig. 3 [file 44319_2024_82_MOESM8_ESM.zip › Figure 3/3J/3J EdU visualization.tiff]

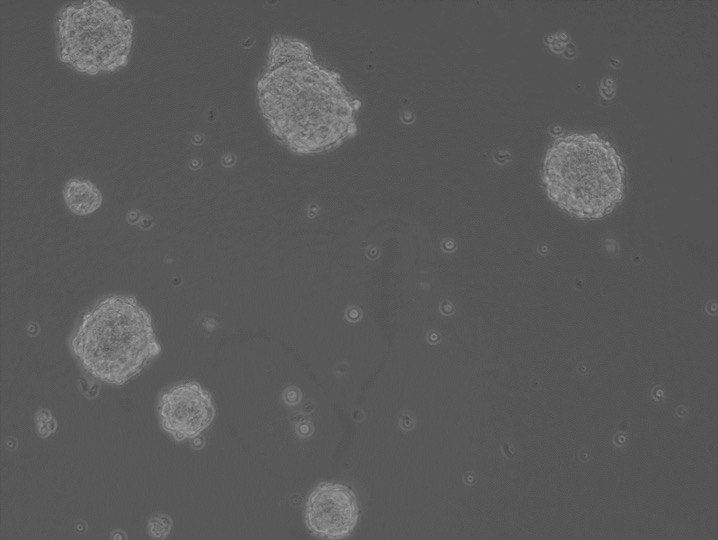

Supplement: Supplementary file 8 — Source Data Fig. 3 [file 44319_2024_82_MOESM8_ESM.zip › Figure 3/3A/3A siPhf6.jpg]

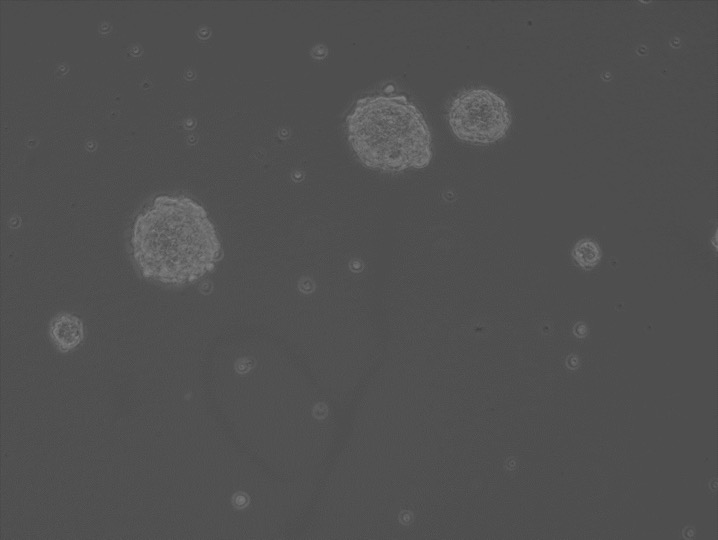

Supplement: Supplementary file 8 — Source Data Fig. 3 [file 44319_2024_82_MOESM8_ESM.zip › Figure 3/3A/3A sictl.jpg]

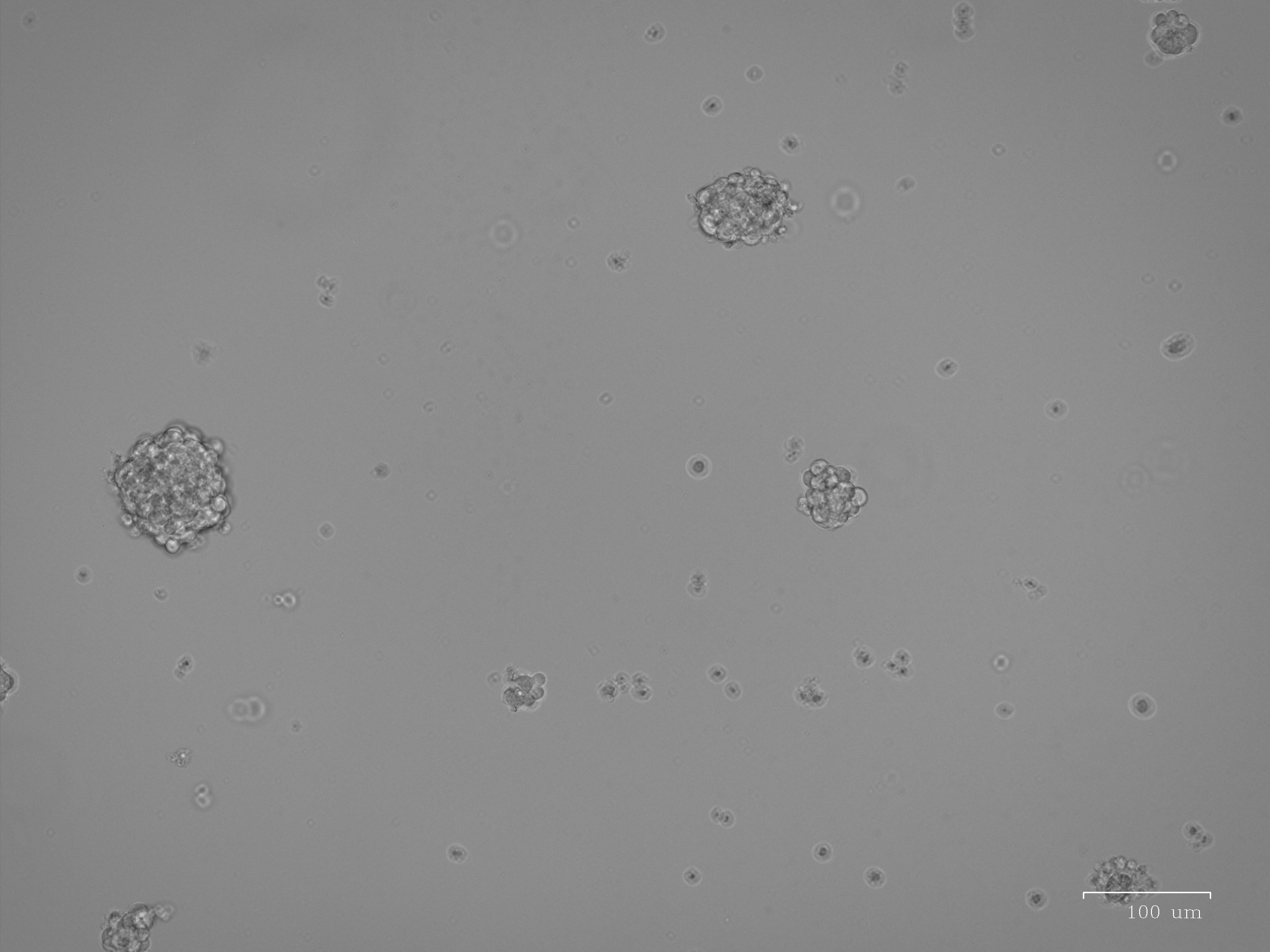

Supplement: Supplementary file 8 — Source Data Fig. 3 [file 44319_2024_82_MOESM8_ESM.zip › Figure 3/3G/3G Phf6 loxp:Y : Nestin-CreERT2-.tif]

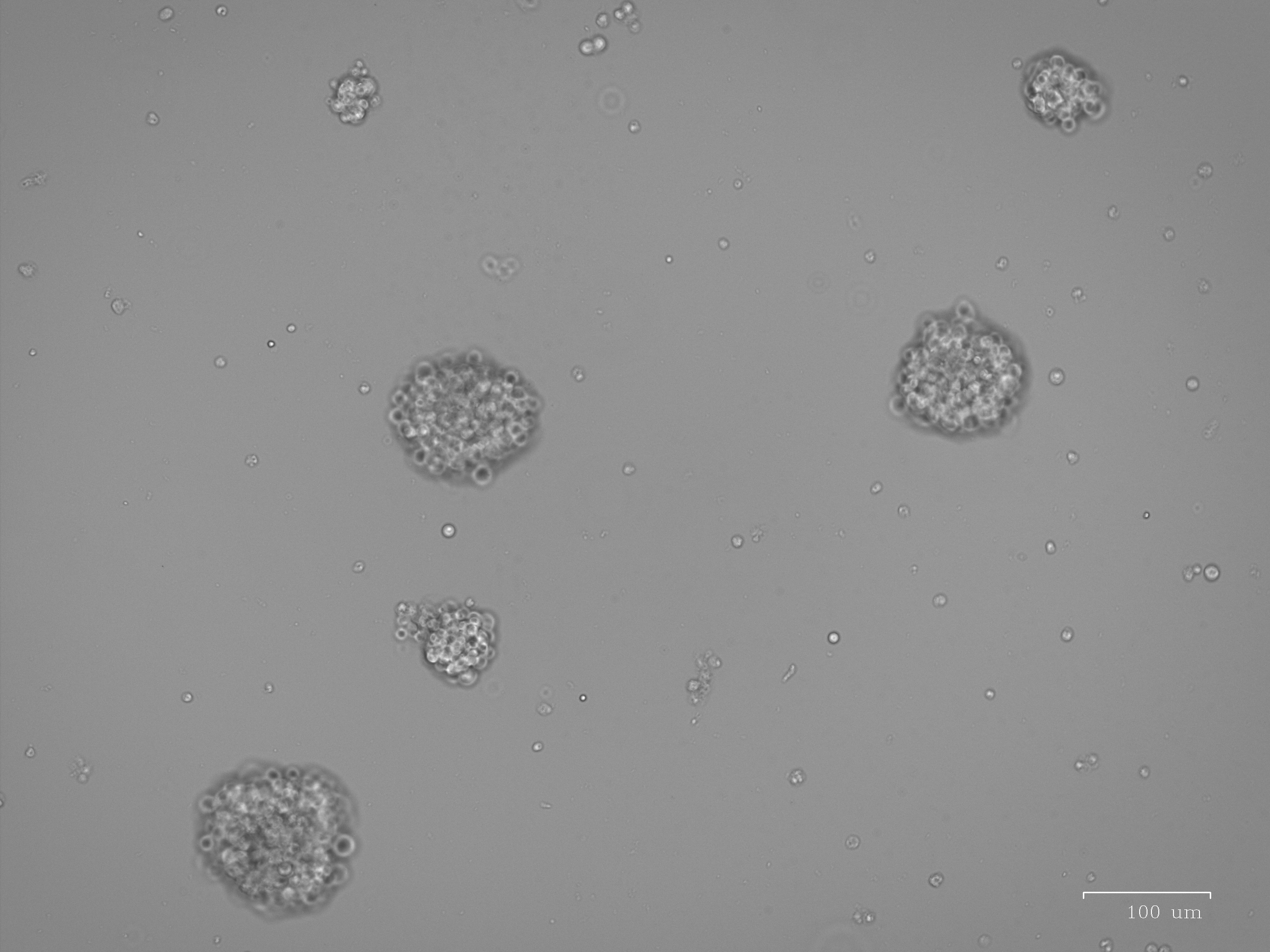

Supplement: Supplementary file 8 — Source Data Fig. 3 [file 44319_2024_82_MOESM8_ESM.zip › Figure 3/3G/3G Phf6 -:Y : Nestin-CreERT2+.tif]

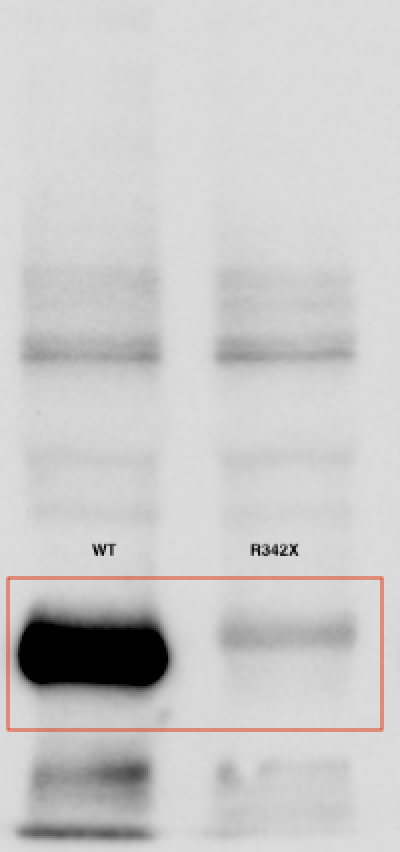

Supplement: Supplementary file 9 — Source Data Fig. 5 [file 44319_2024_82_MOESM9_ESM.zip › Figure 5/5D/western PHF6.png]

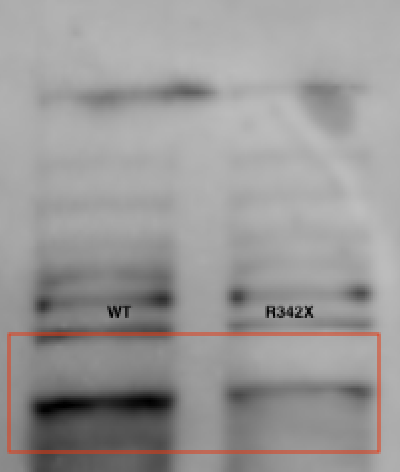

Supplement: Supplementary file 9 — Source Data Fig. 5 [file 44319_2024_82_MOESM9_ESM.zip › Figure 5/5D/western EPHA7.png]

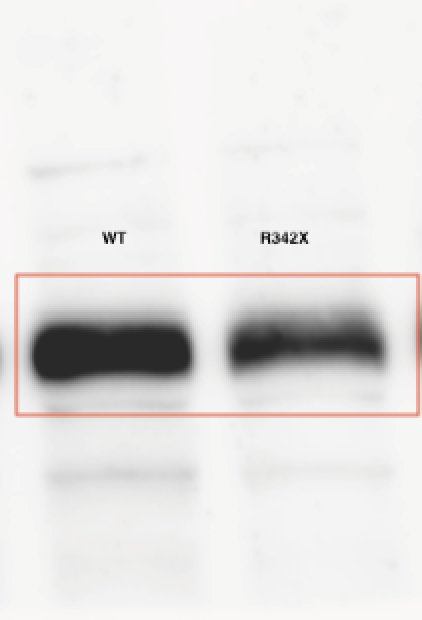

Supplement: Supplementary file 9 — Source Data Fig. 5 [file 44319_2024_82_MOESM9_ESM.zip › Figure 5/5D/western EPHA4.png]

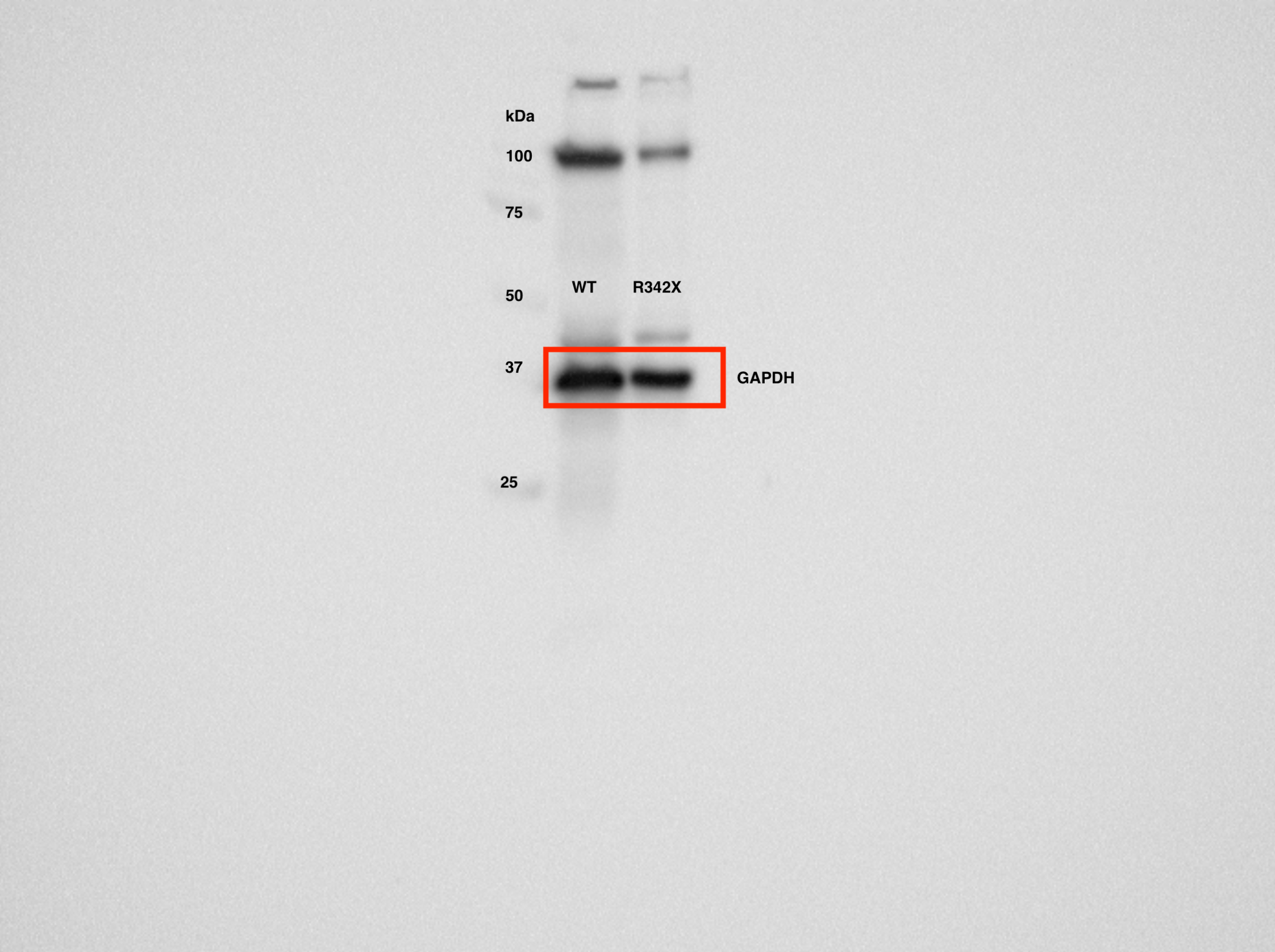

Supplement: Supplementary file 9 — Source Data Fig. 5 [file 44319_2024_82_MOESM9_ESM.zip › Figure 5/5D/western GAPDH.tiff]

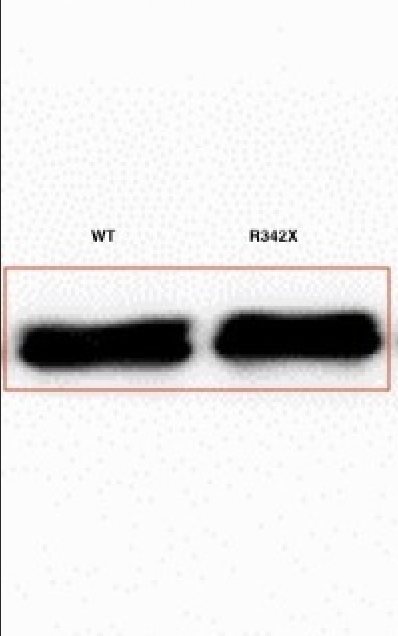

Supplement: Supplementary file 9 — Source Data Fig. 5 [file 44319_2024_82_MOESM9_ESM.zip › Figure 5/5D/western TUBULIN.png]

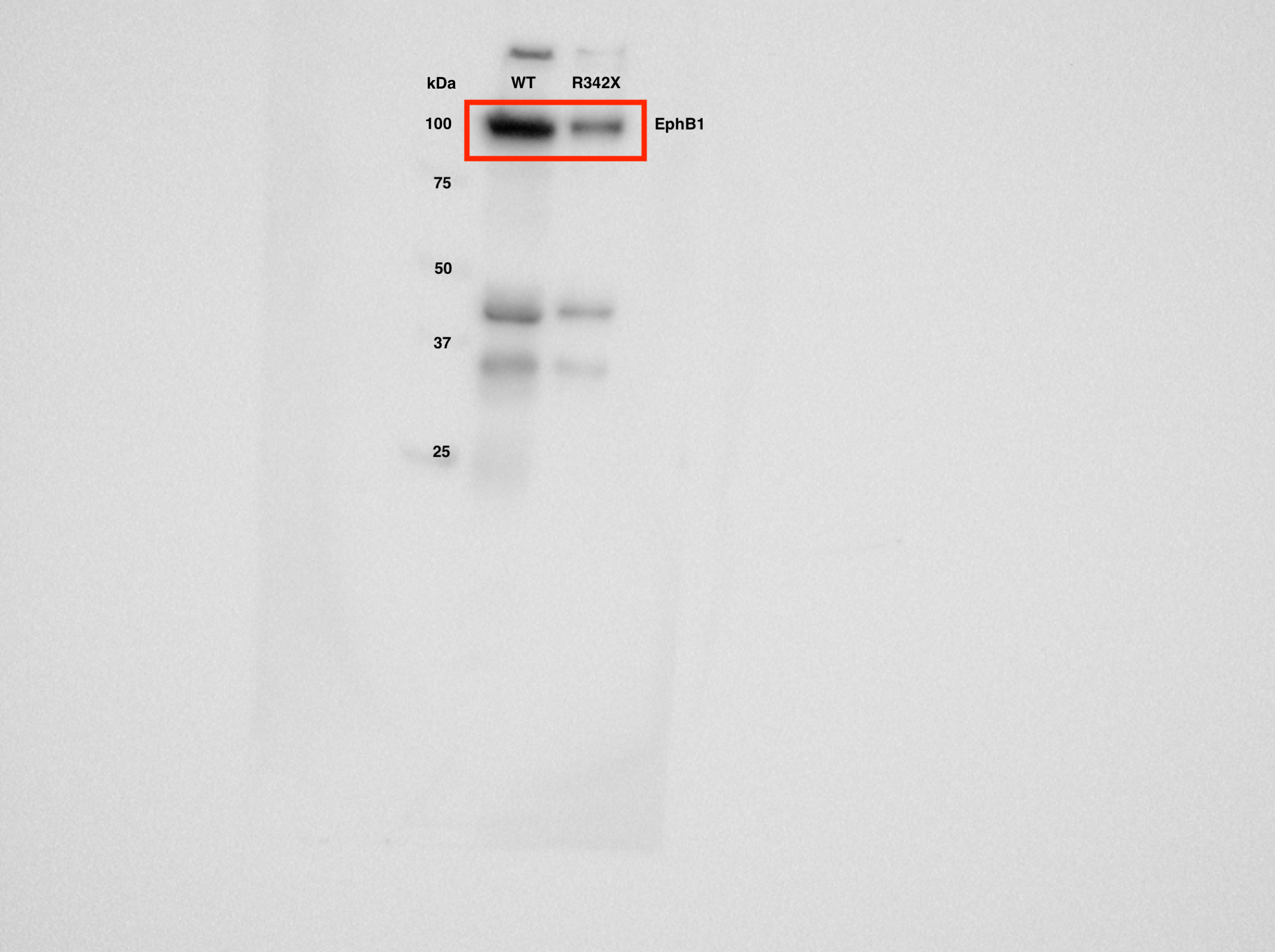

Supplement: Supplementary file 9 — Source Data Fig. 5 [file 44319_2024_82_MOESM9_ESM.zip › Figure 5/5D/western EPHB1.tiff]

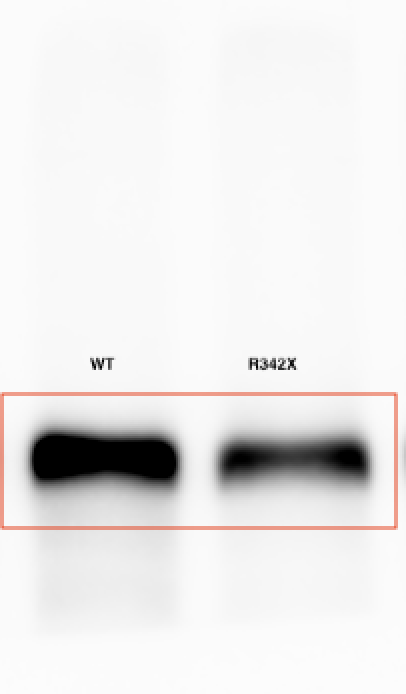

Supplement: Supplementary file 9 — Source Data Fig. 5 [file 44319_2024_82_MOESM9_ESM.zip › Figure 5/5D/western EPHB2.png]

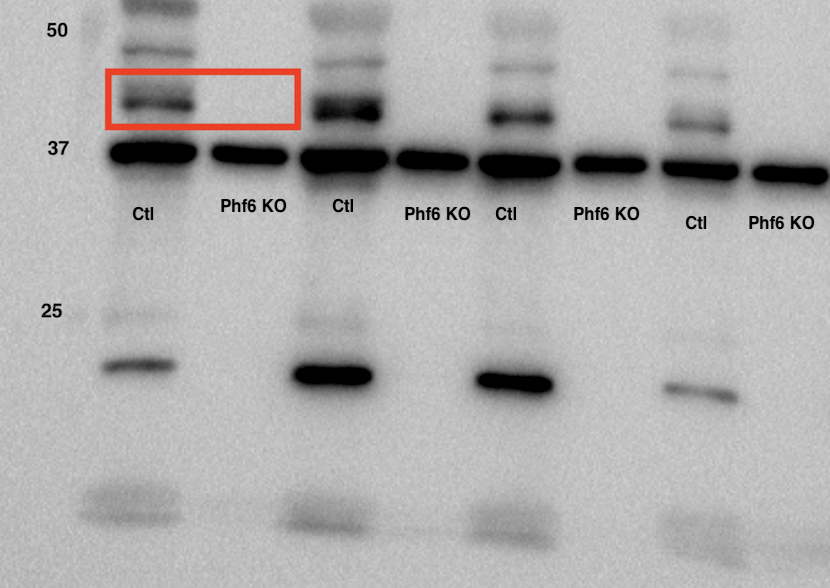

Supplement: Supplementary file 9 — Source Data Fig. 5 [file 44319_2024_82_MOESM9_ESM.zip › Figure 5/5B/western PHF6.png]

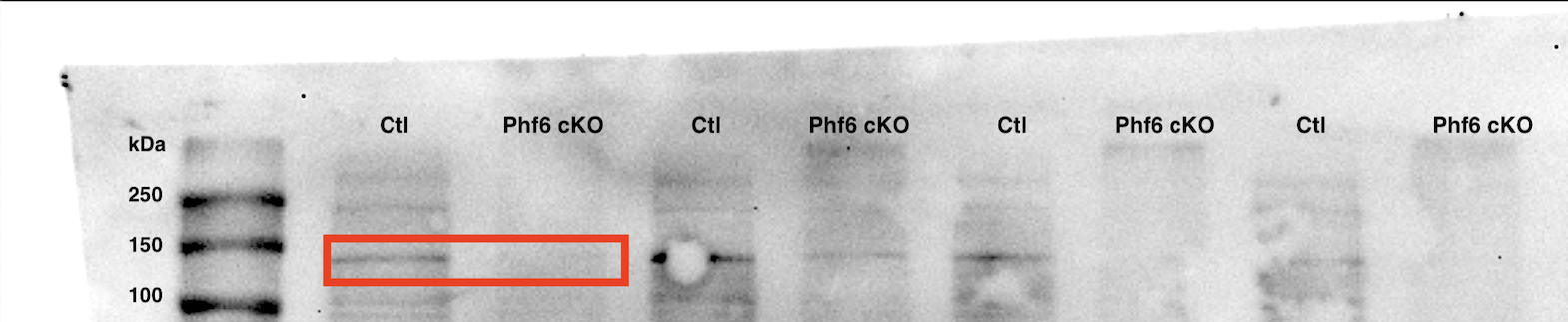

Supplement: Supplementary file 9 — Source Data Fig. 5 [file 44319_2024_82_MOESM9_ESM.zip › Figure 5/5B/western EPHA7.png]

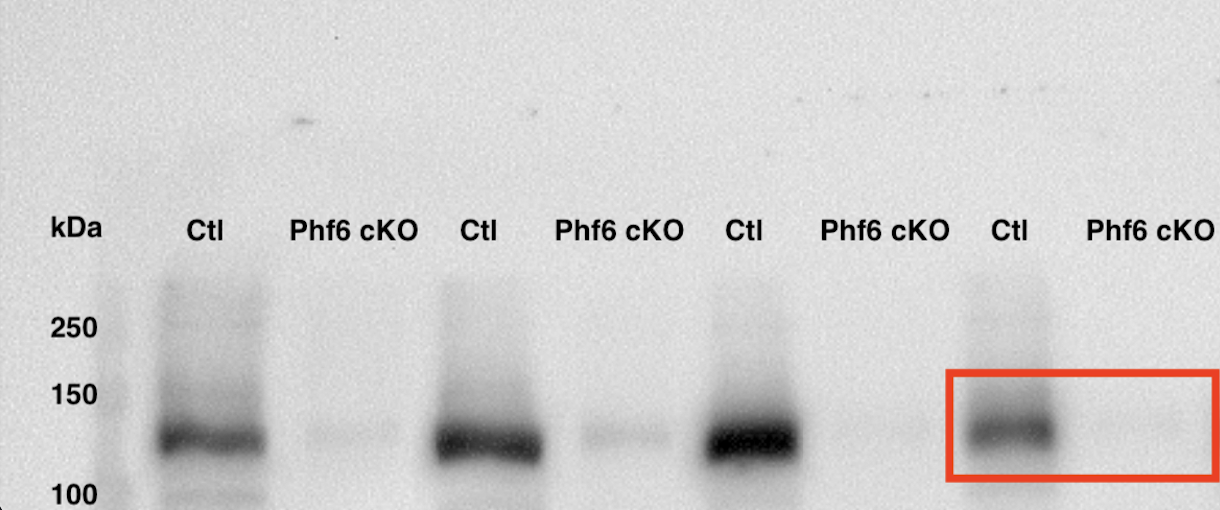

Supplement: Supplementary file 9 — Source Data Fig. 5 [file 44319_2024_82_MOESM9_ESM.zip › Figure 5/5B/western EPHA4.png]

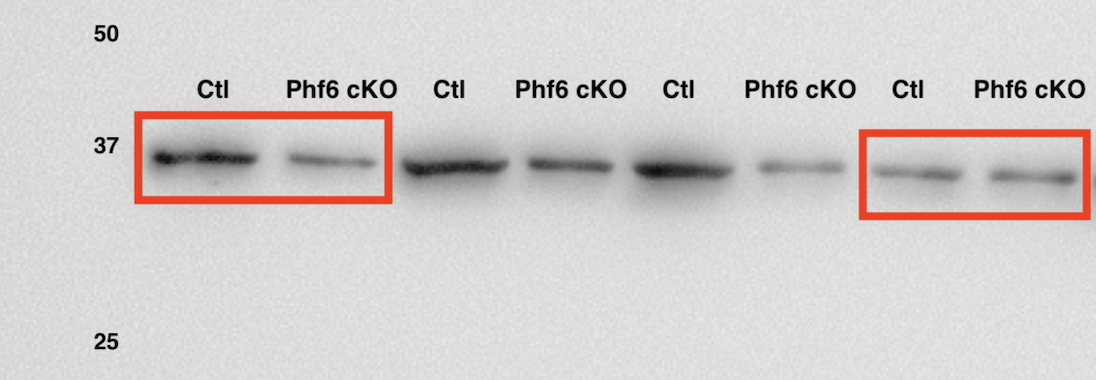

Supplement: Supplementary file 9 — Source Data Fig. 5 [file 44319_2024_82_MOESM9_ESM.zip › Figure 5/5B/western GAPDH.png]

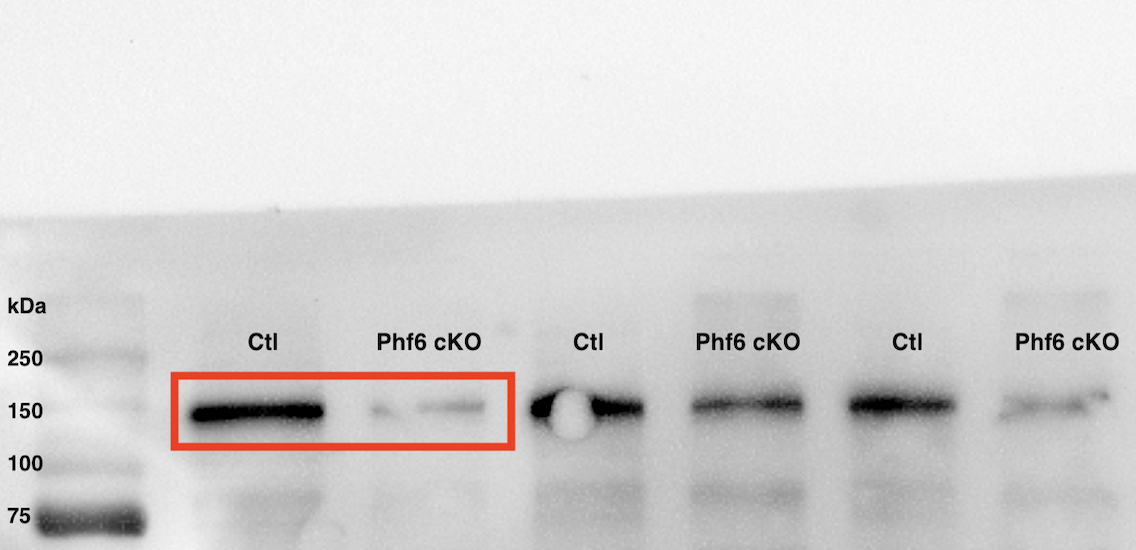

Supplement: Supplementary file 9 — Source Data Fig. 5 [file 44319_2024_82_MOESM9_ESM.zip › Figure 5/5B/western EPHB2.png]

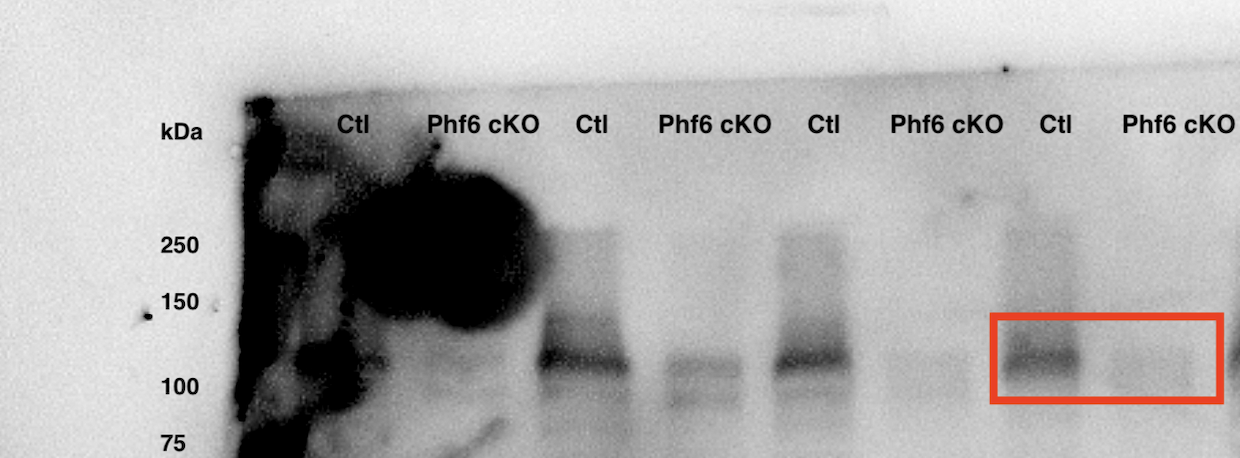

Supplement: Supplementary file 9 — Source Data Fig. 5 [file 44319_2024_82_MOESM9_ESM.zip › Figure 5/5B/western EPHB1.png]

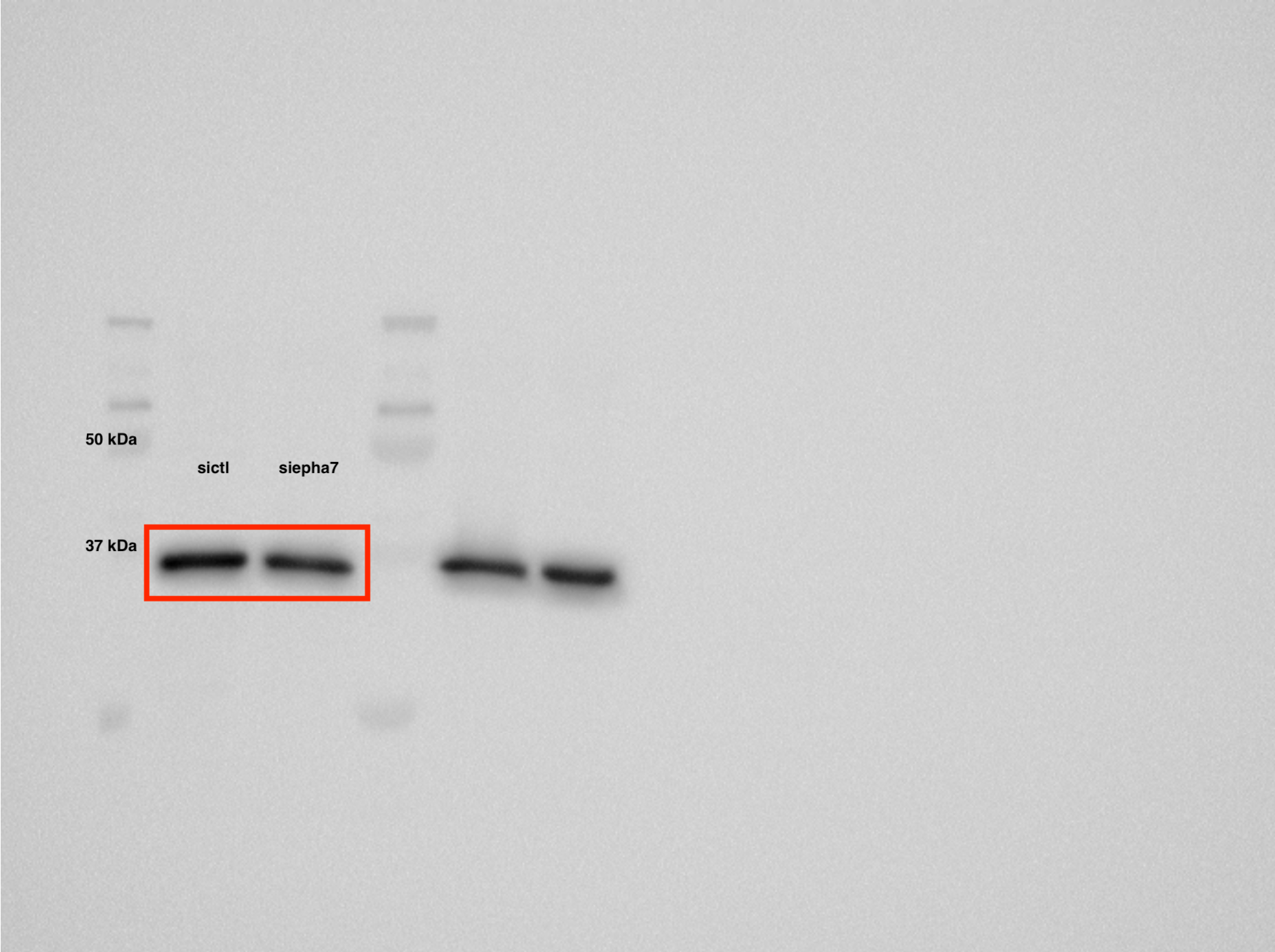

Supplement: Supplementary file 10 — Source Data Fig. 6 [file 44319_2024_82_MOESM10_ESM.zip › Figure 6/6F/western GAPDH.tiff]

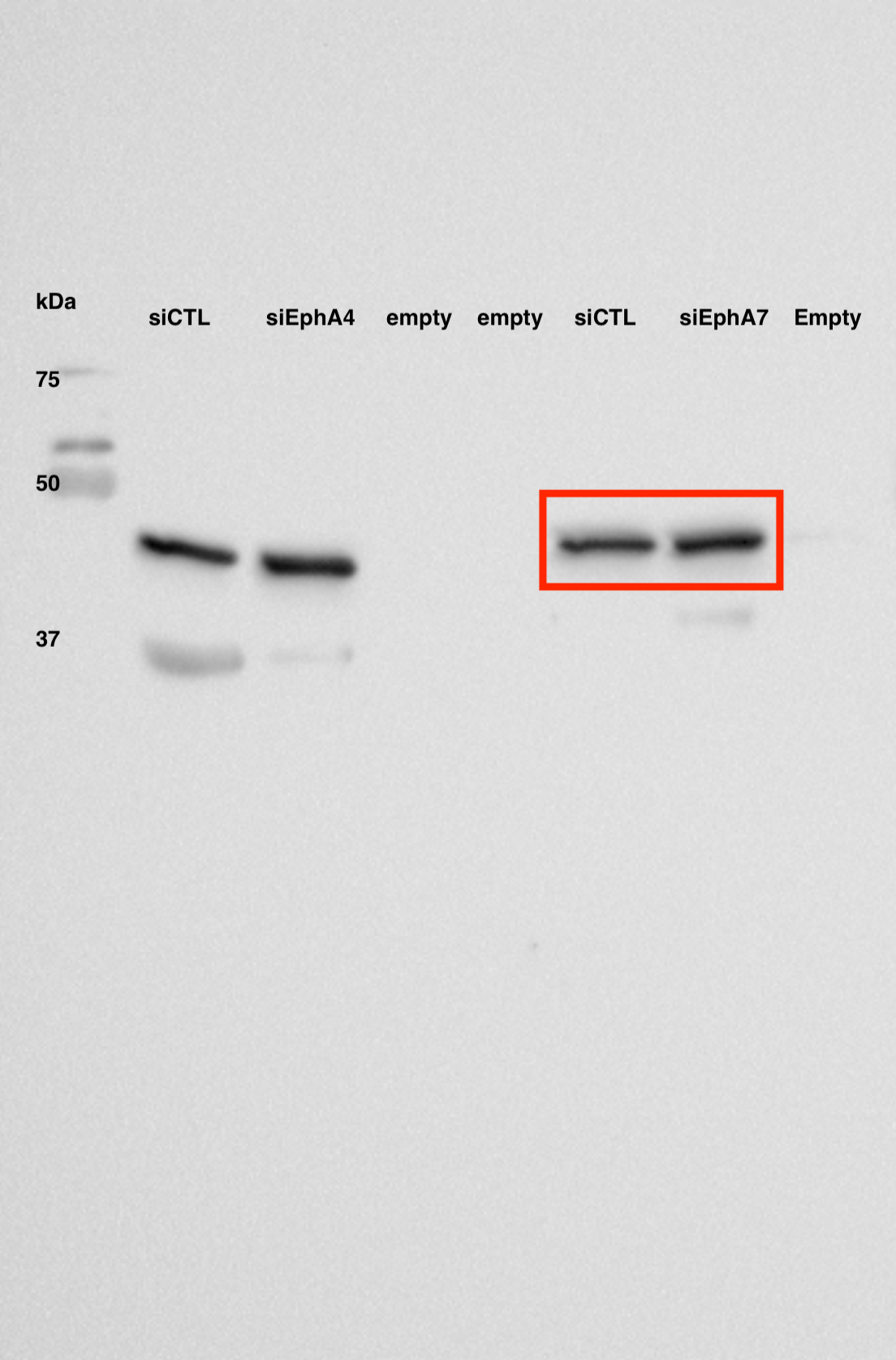

Supplement: Supplementary file 10 — Source Data Fig. 6 [file 44319_2024_82_MOESM10_ESM.zip › Figure 6/6F/western ACTIN.tiff]

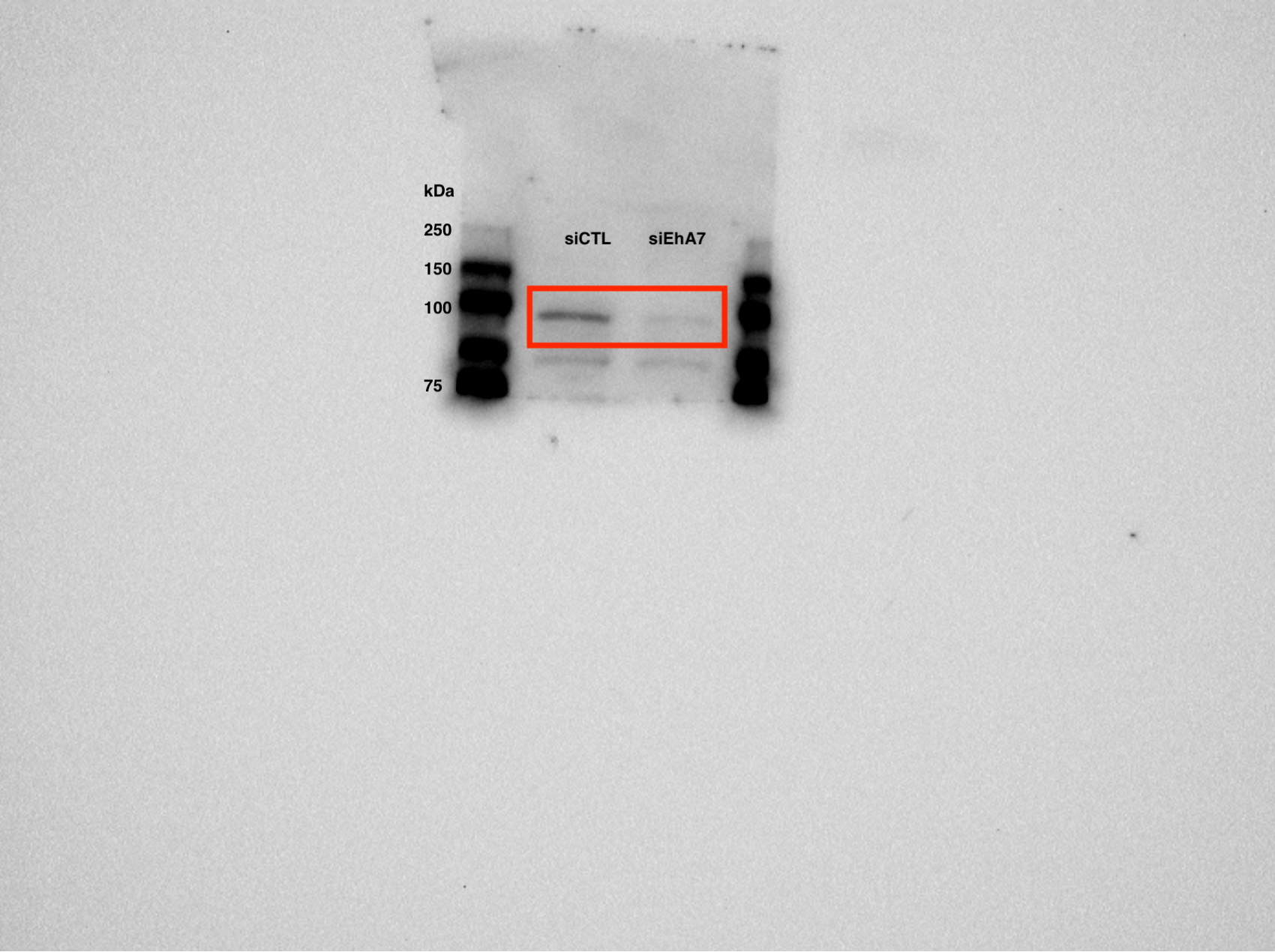

Supplement: Supplementary file 10 — Source Data Fig. 6 [file 44319_2024_82_MOESM10_ESM.zip › Figure 6/6F/western EPHA7.tiff]

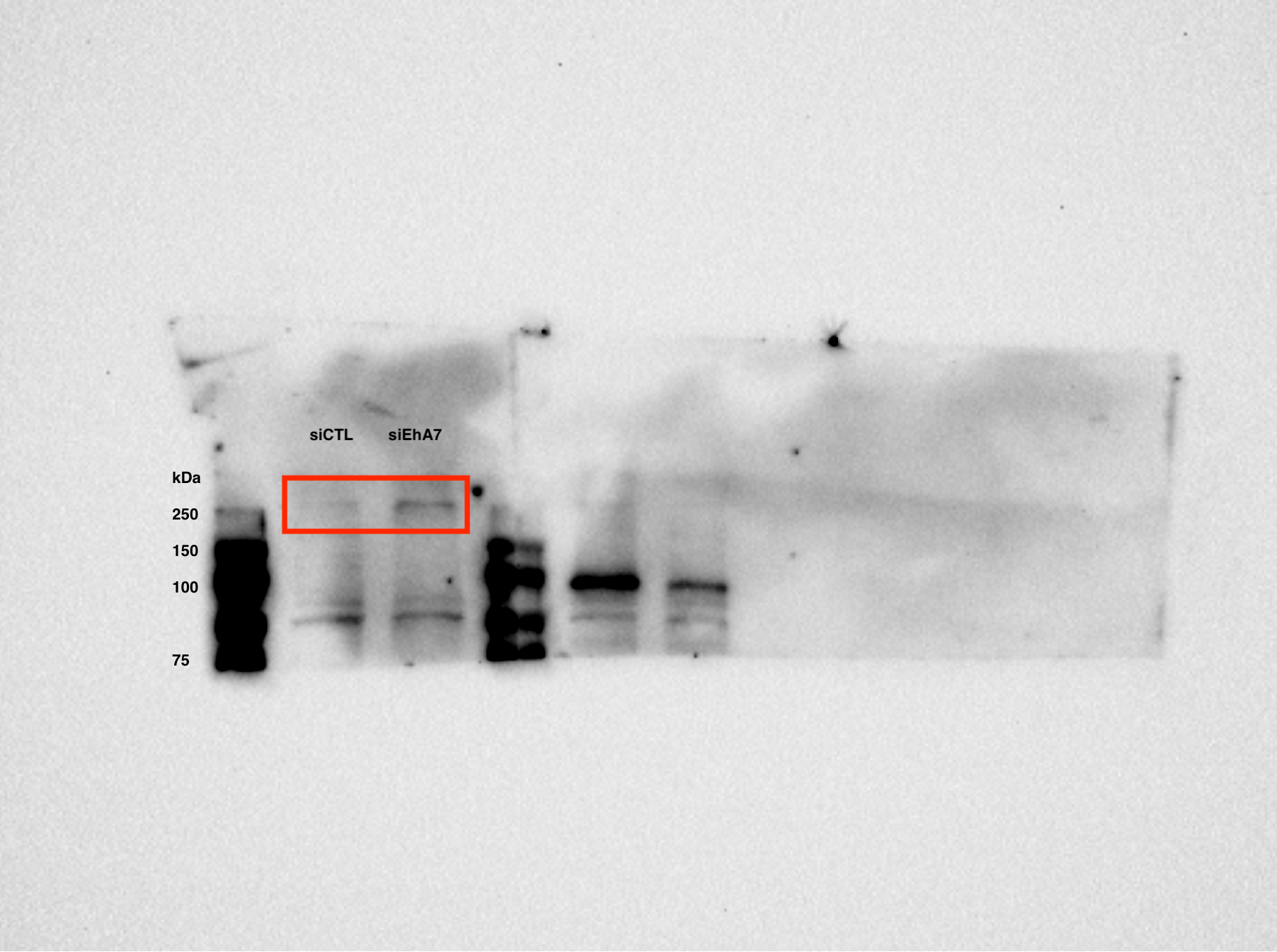

Supplement: Supplementary file 10 — Source Data Fig. 6 [file 44319_2024_82_MOESM10_ESM.zip › Figure 6/6F/western NESTIN.tiff]

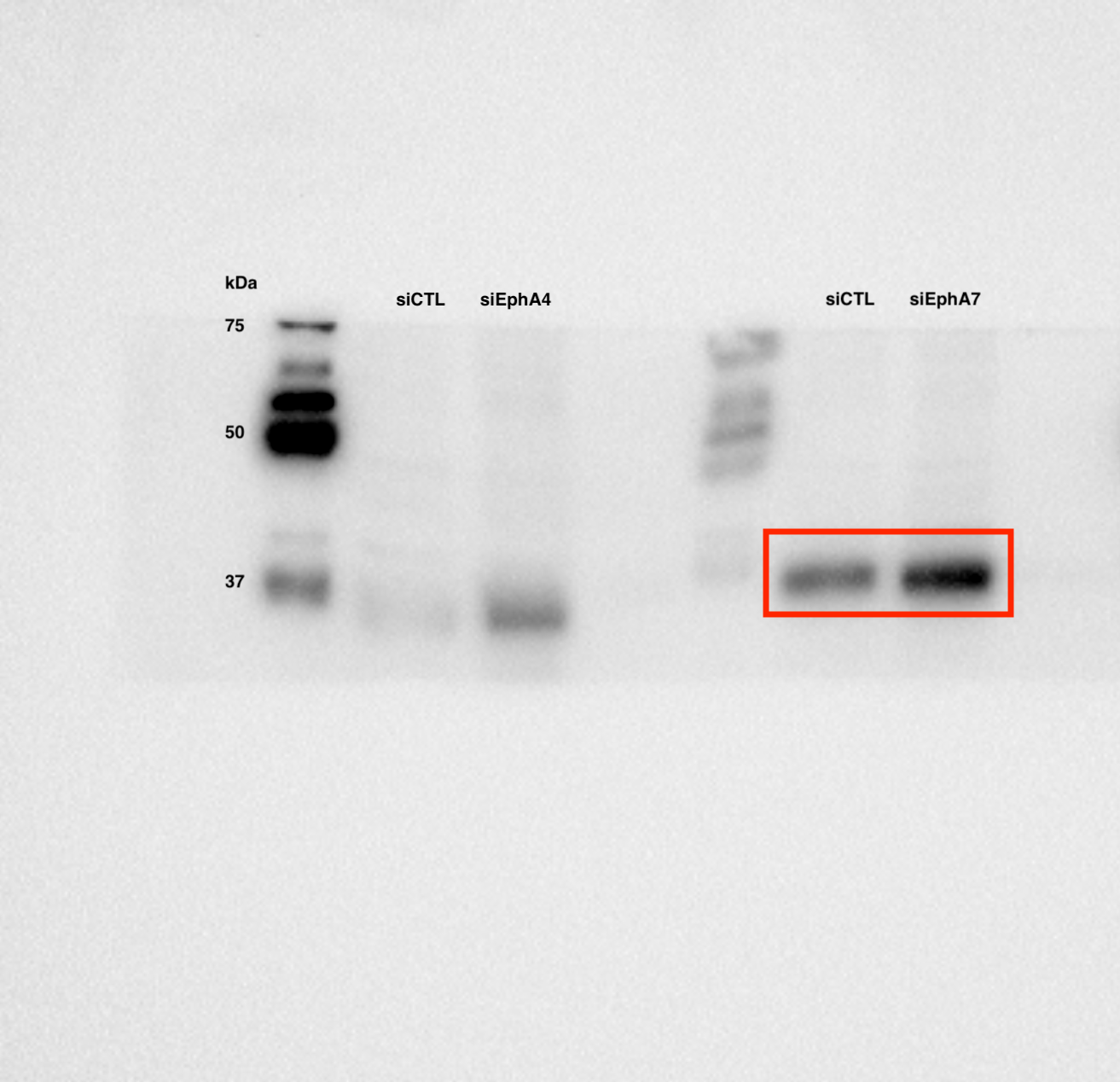

Supplement: Supplementary file 10 — Source Data Fig. 6 [file 44319_2024_82_MOESM10_ESM.zip › Figure 6/6F/western SOX2.tiff]

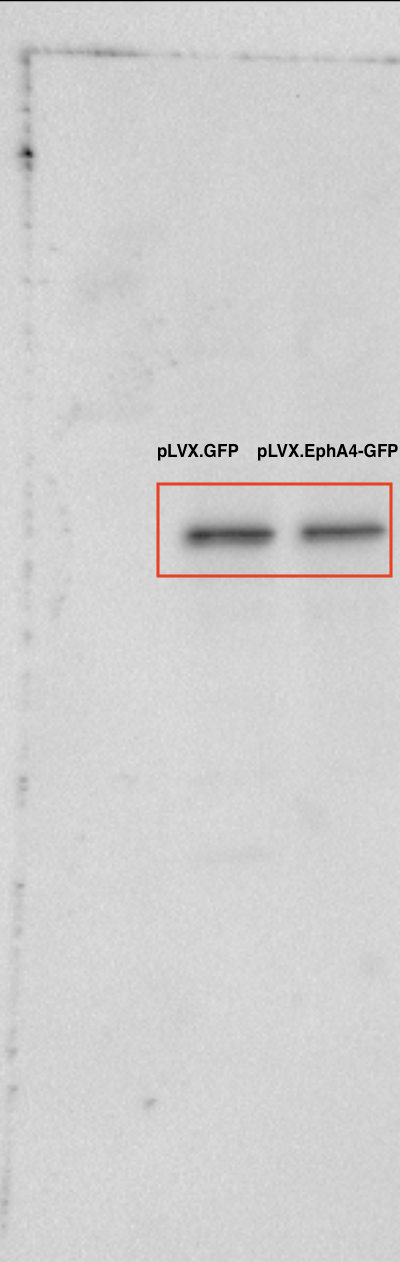

Supplement: Supplementary file 10 — Source Data Fig. 6 [file 44319_2024_82_MOESM10_ESM.zip › Figure 6/6I/western TUBULIN.png]

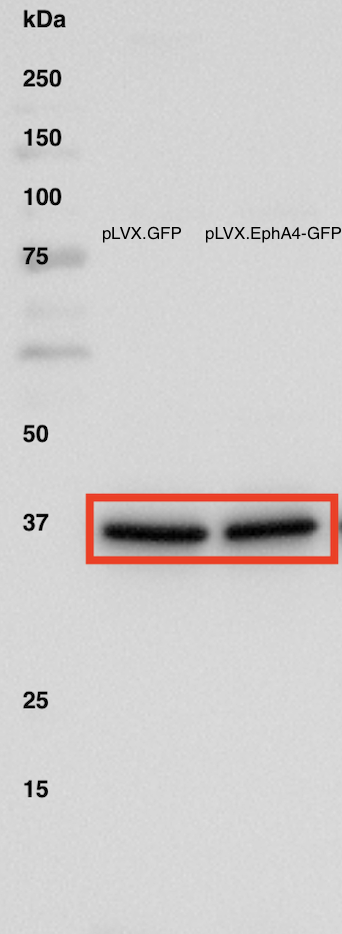

Supplement: Supplementary file 10 — Source Data Fig. 6 [file 44319_2024_82_MOESM10_ESM.zip › Figure 6/6I/western GAPDH.png]

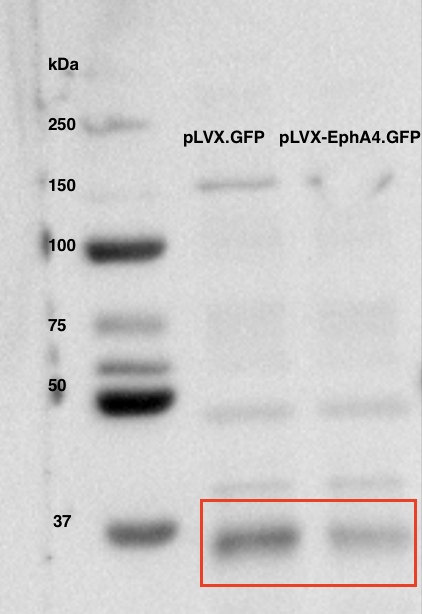

Supplement: Supplementary file 10 — Source Data Fig. 6 [file 44319_2024_82_MOESM10_ESM.zip › Figure 6/6I/western SOX2.png]

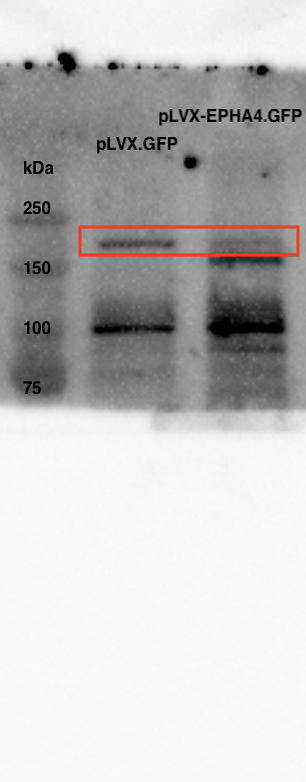

Supplement: Supplementary file 10 — Source Data Fig. 6 [file 44319_2024_82_MOESM10_ESM.zip › Figure 6/6I/western NESTIN.png]

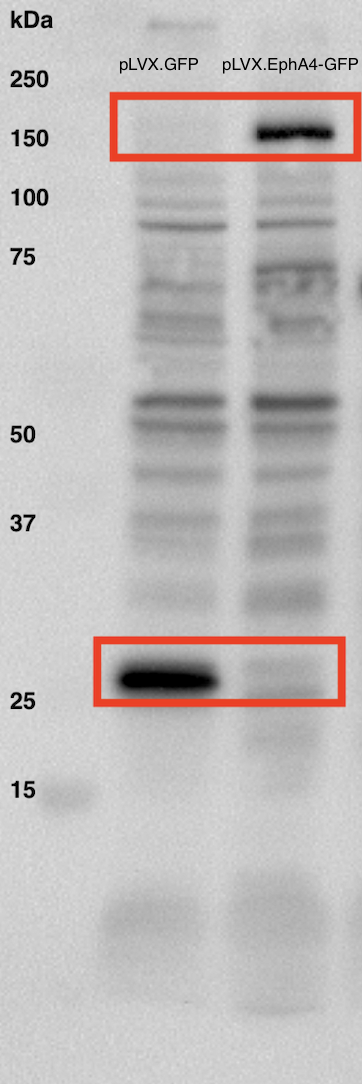

Supplement: Supplementary file 10 — Source Data Fig. 6 [file 44319_2024_82_MOESM10_ESM.zip › Figure 6/6I/western GFP and GFP-EPHA4.png]

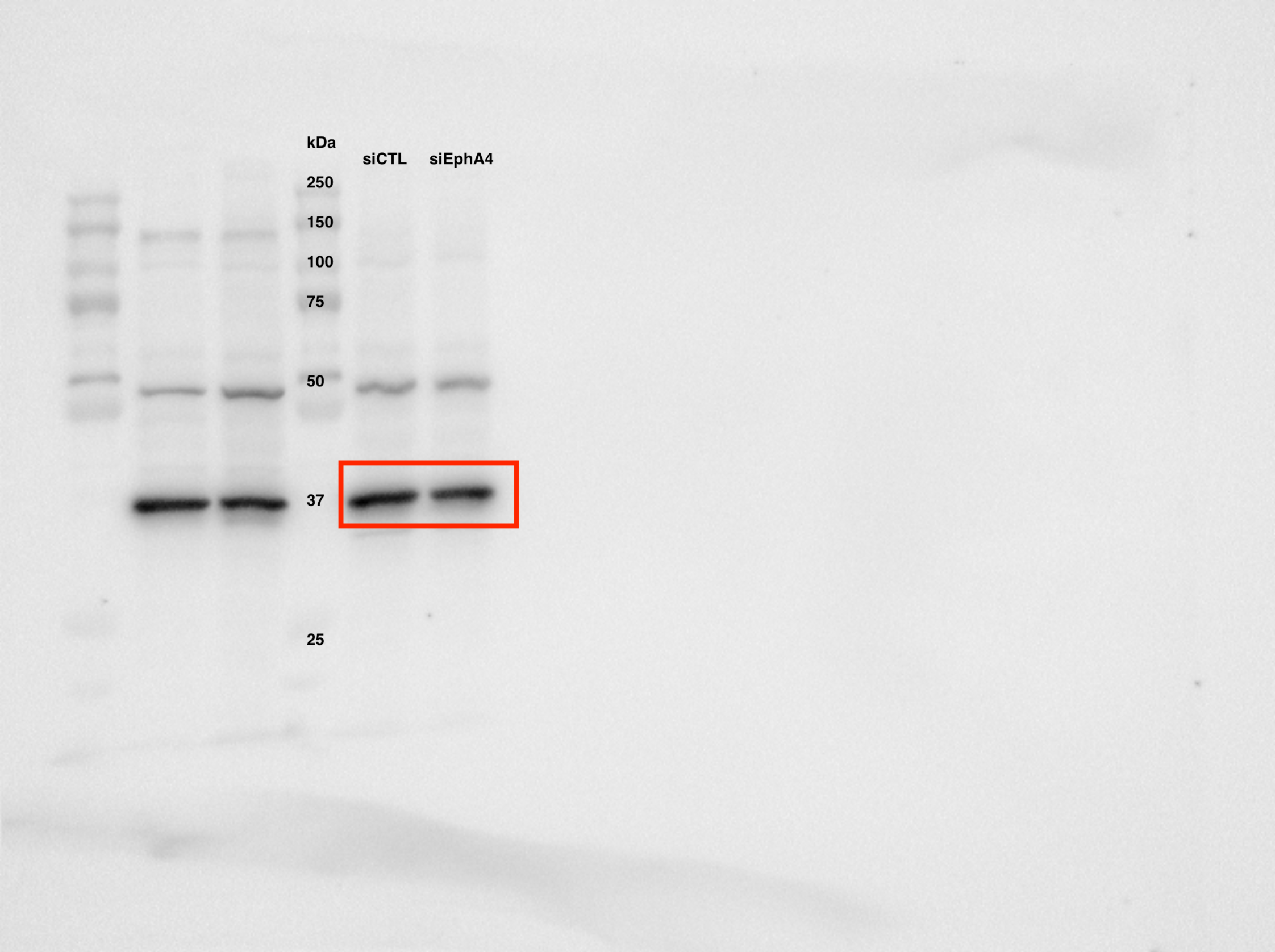

Supplement: Supplementary file 10 — Source Data Fig. 6 [file 44319_2024_82_MOESM10_ESM.zip › Figure 6/6E/western GAPDH.tiff]

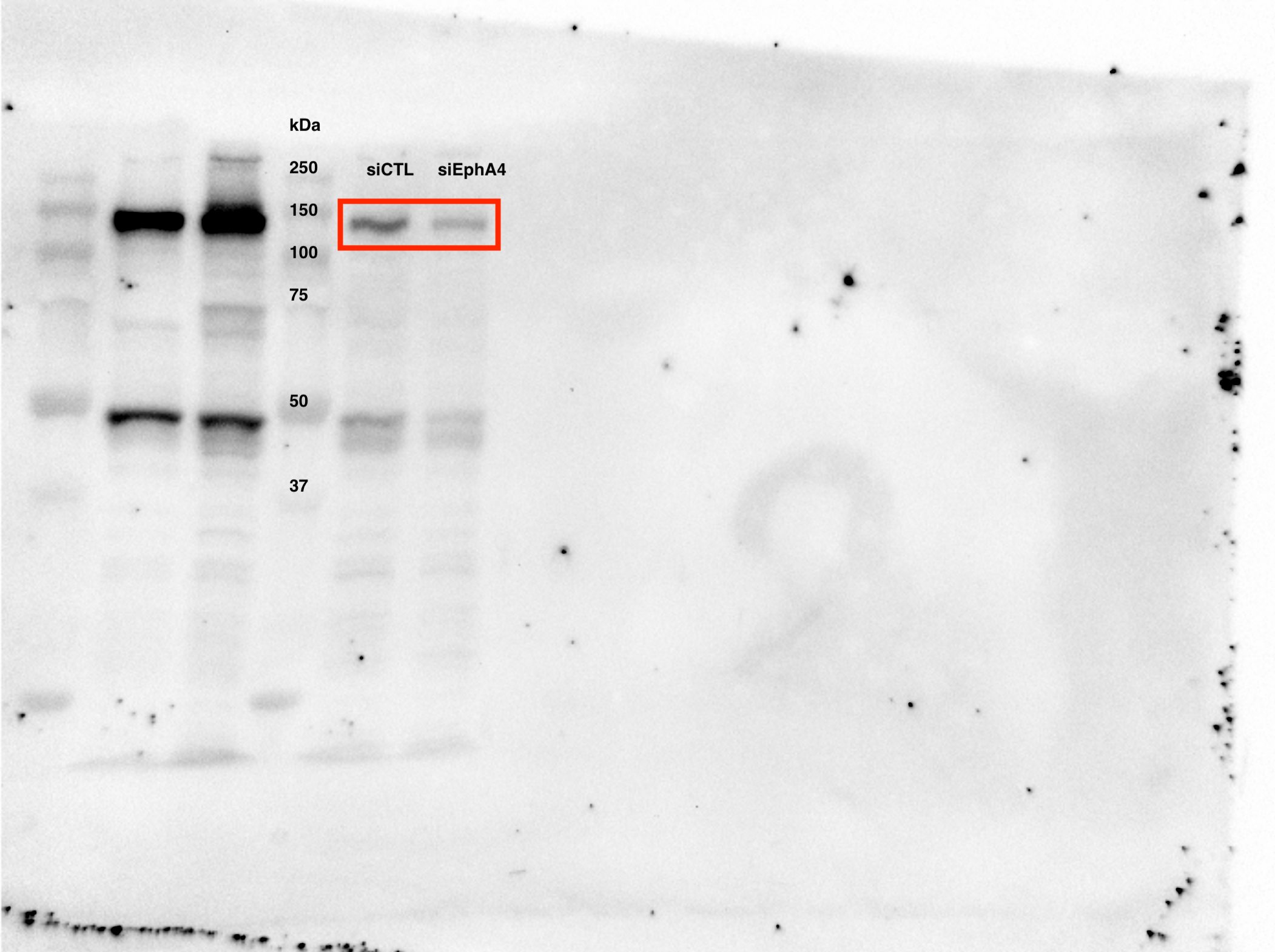

Supplement: Supplementary file 10 — Source Data Fig. 6 [file 44319_2024_82_MOESM10_ESM.zip › Figure 6/6E/western EPHA4.tiff]

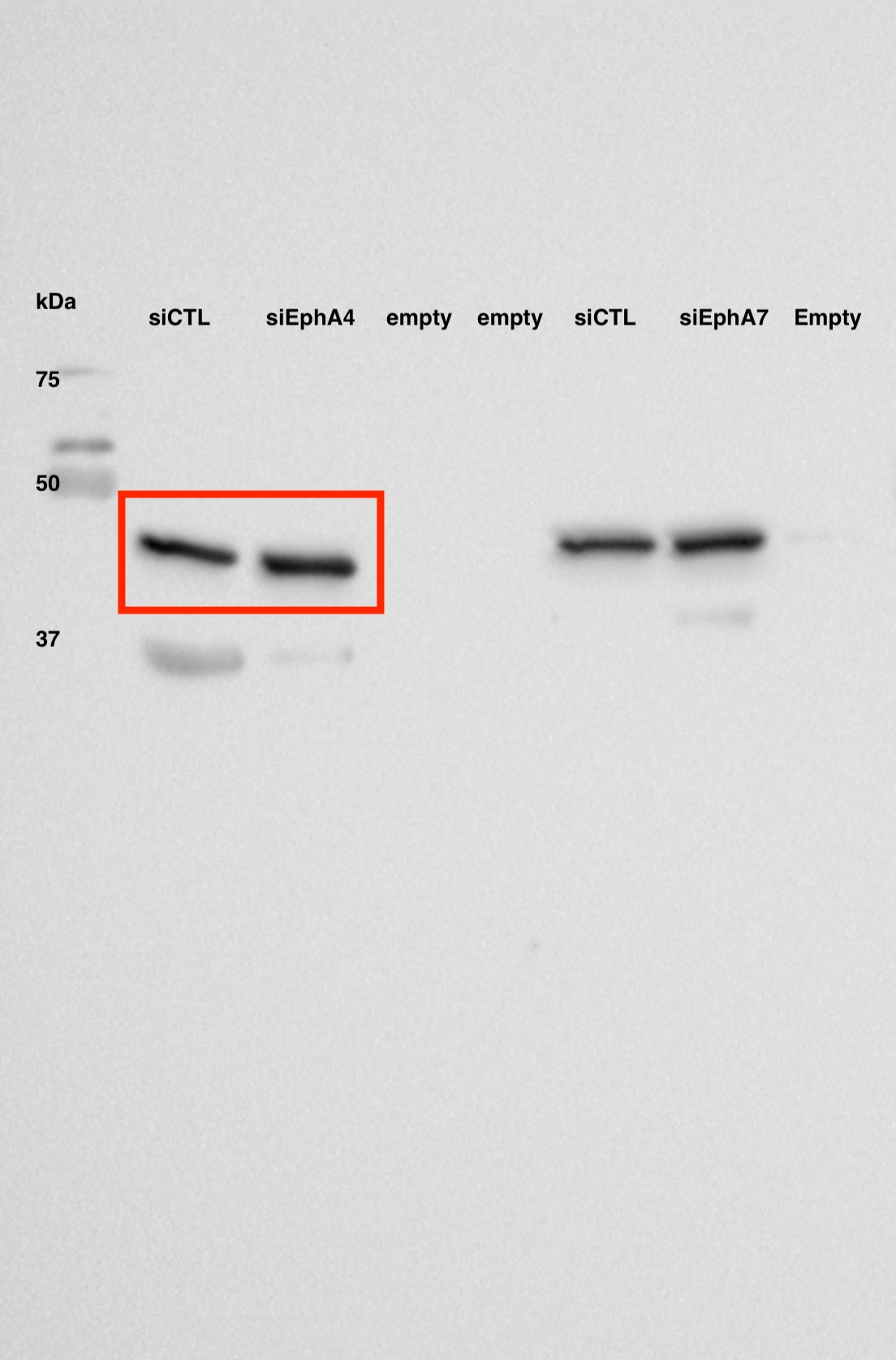

Supplement: Supplementary file 10 — Source Data Fig. 6 [file 44319_2024_82_MOESM10_ESM.zip › Figure 6/6E/western ACTIN.tiff]

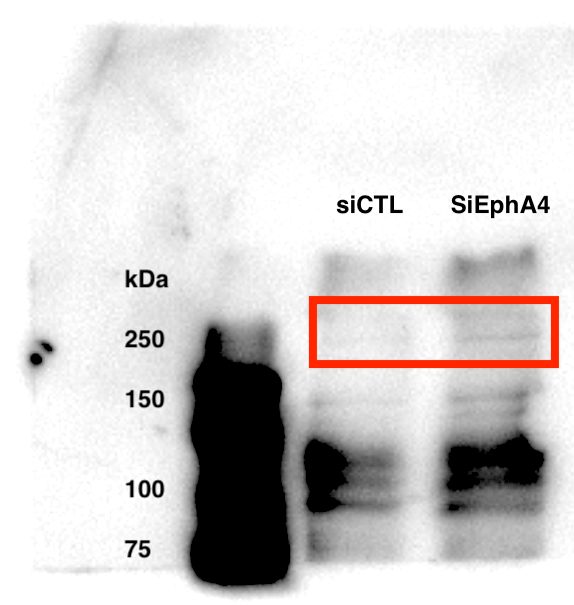

Supplement: Supplementary file 10 — Source Data Fig. 6 [file 44319_2024_82_MOESM10_ESM.zip › Figure 6/6E/Western NESTIN.tiff]

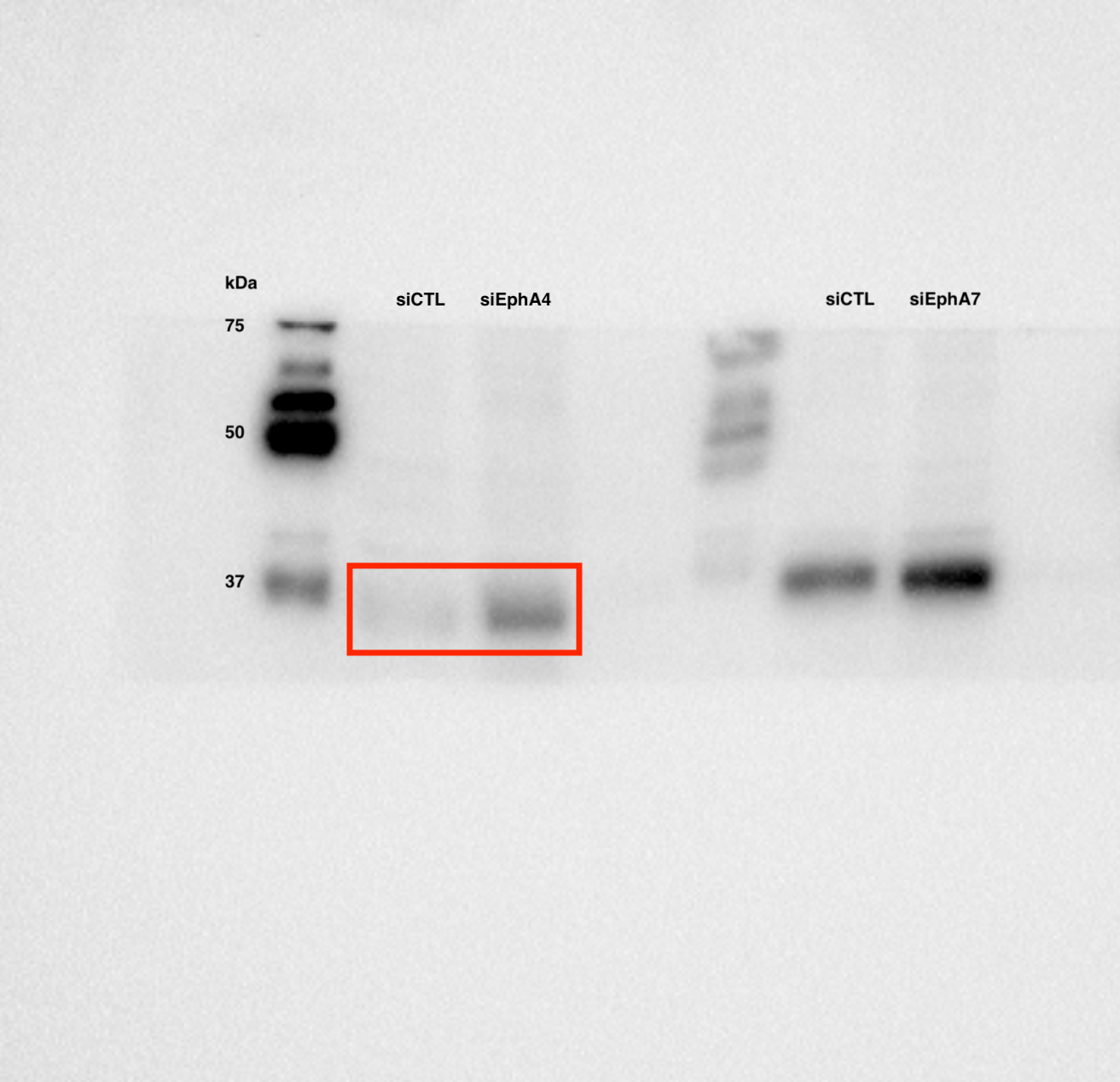

Supplement: Supplementary file 10 — Source Data Fig. 6 [file 44319_2024_82_MOESM10_ESM.zip › Figure 6/6E/western SOX2.tiff]

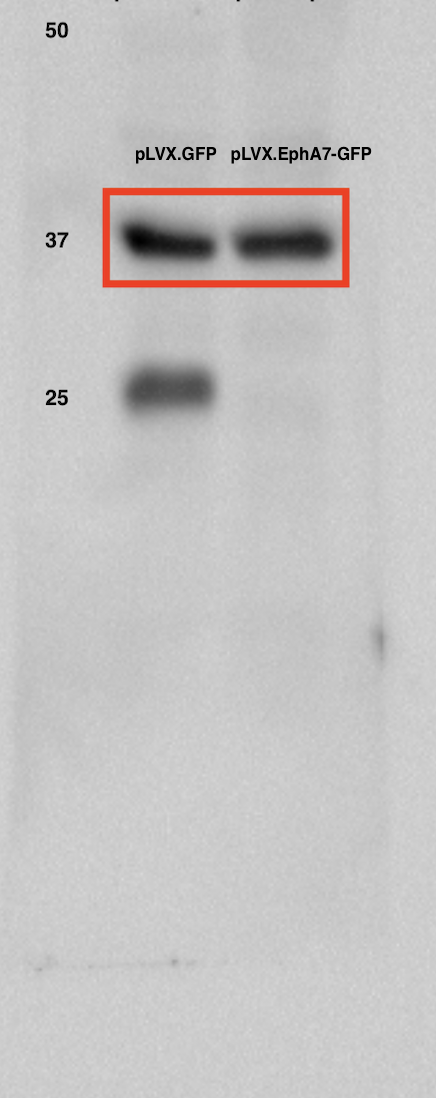

Supplement: Supplementary file 10 — Source Data Fig. 6 [file 44319_2024_82_MOESM10_ESM.zip › Figure 6/6L/western GAPDH.png]

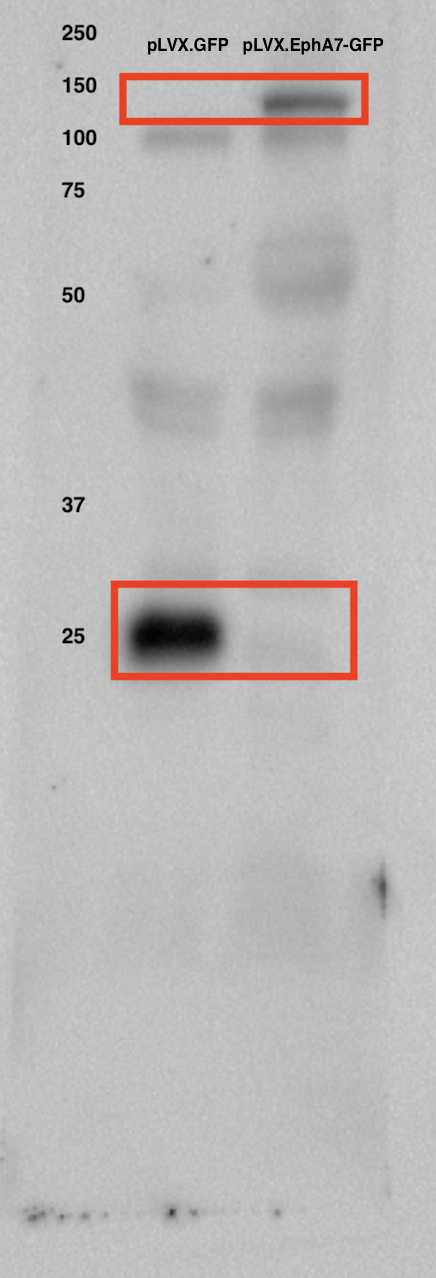

Supplement: Supplementary file 10 — Source Data Fig. 6 [file 44319_2024_82_MOESM10_ESM.zip › Figure 6/6L/western GFP and GFP-EphA7.png]

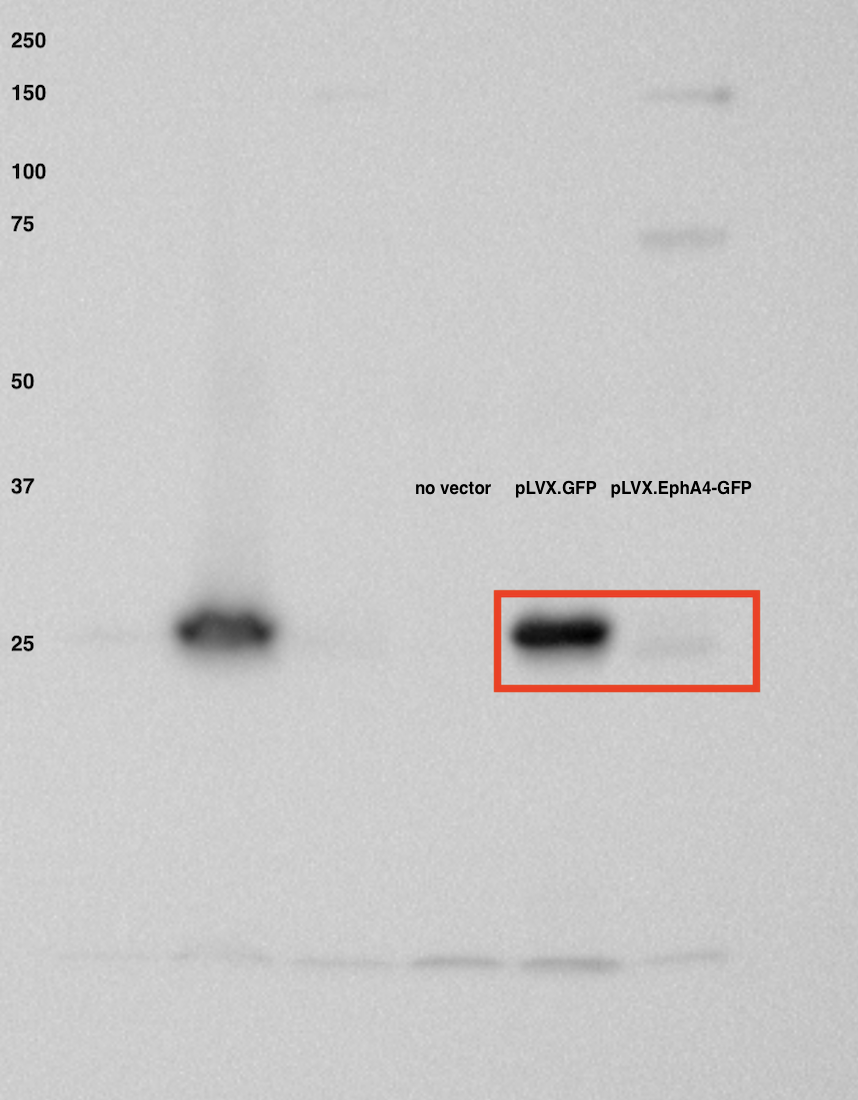

Supplement: Supplementary file 10 — Source Data Fig. 6 [file 44319_2024_82_MOESM10_ESM.zip › Figure 6/6K/western GFP.png]

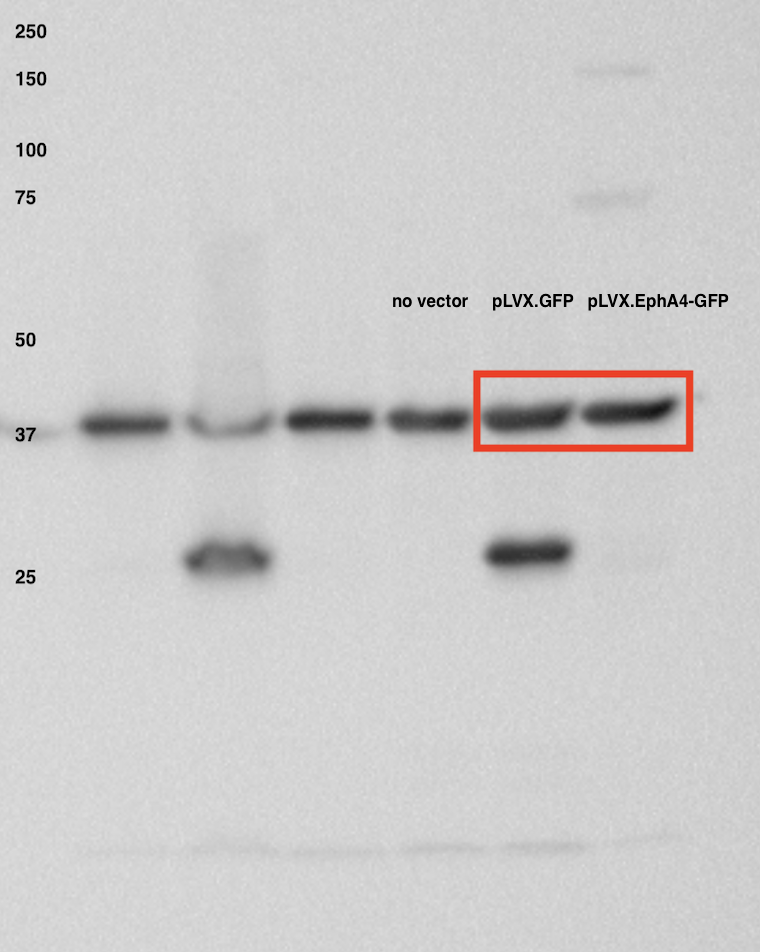

Supplement: Supplementary file 10 — Source Data Fig. 6 [file 44319_2024_82_MOESM10_ESM.zip › Figure 6/6K/western GAPDH.png]

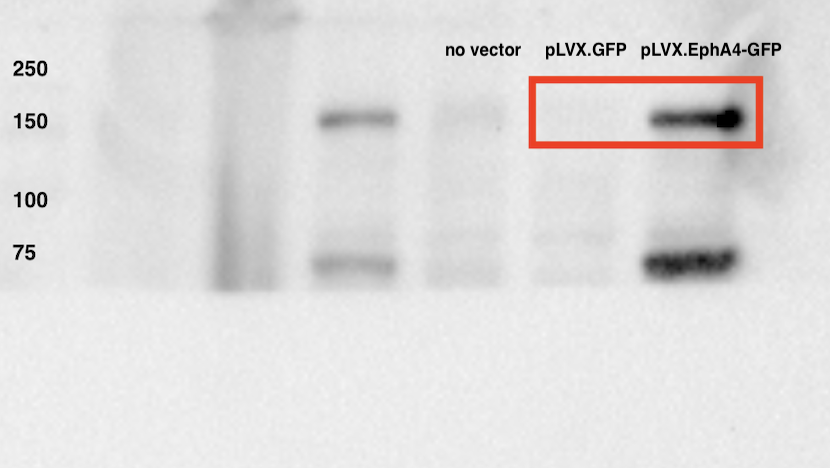

Supplement: Supplementary file 10 — Source Data Fig. 6 [file 44319_2024_82_MOESM10_ESM.zip › Figure 6/6K/western GFP-EphA4.png]

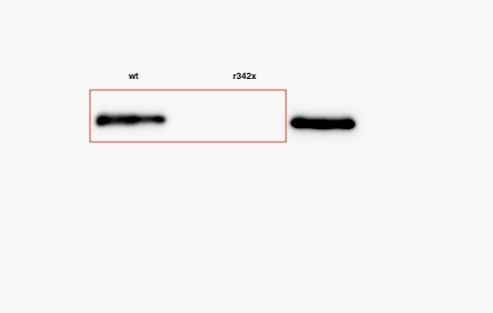

Supplement: Supplementary file 11 — Source Data Fig. 7 [file 44319_2024_82_MOESM11_ESM.zip › Figure 7/7B/western phf6.jpg]

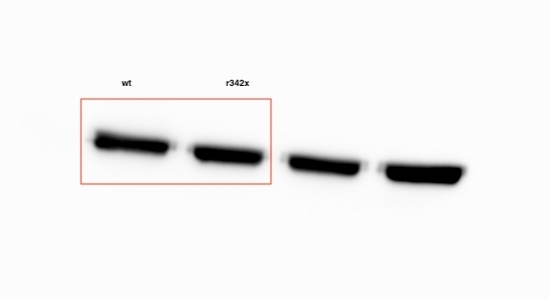

Supplement: Supplementary file 11 — Source Data Fig. 7 [file 44319_2024_82_MOESM11_ESM.zip › Figure 7/7B/western tubulin.jpg]

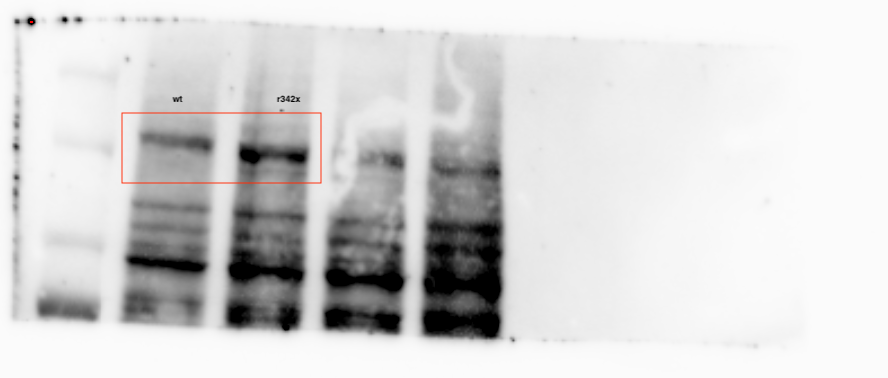

Supplement: Supplementary file 11 — Source Data Fig. 7 [file 44319_2024_82_MOESM11_ESM.zip › Figure 7/7B/western nestin.tif]

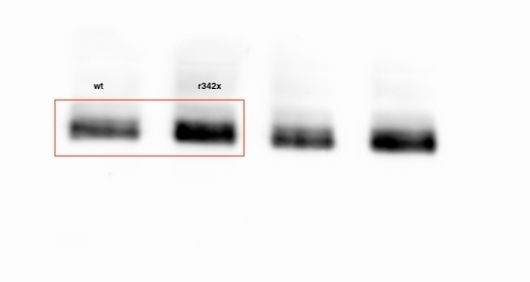

Supplement: Supplementary file 11 — Source Data Fig. 7 [file 44319_2024_82_MOESM11_ESM.zip › Figure 7/7B/western sox2.jpg]

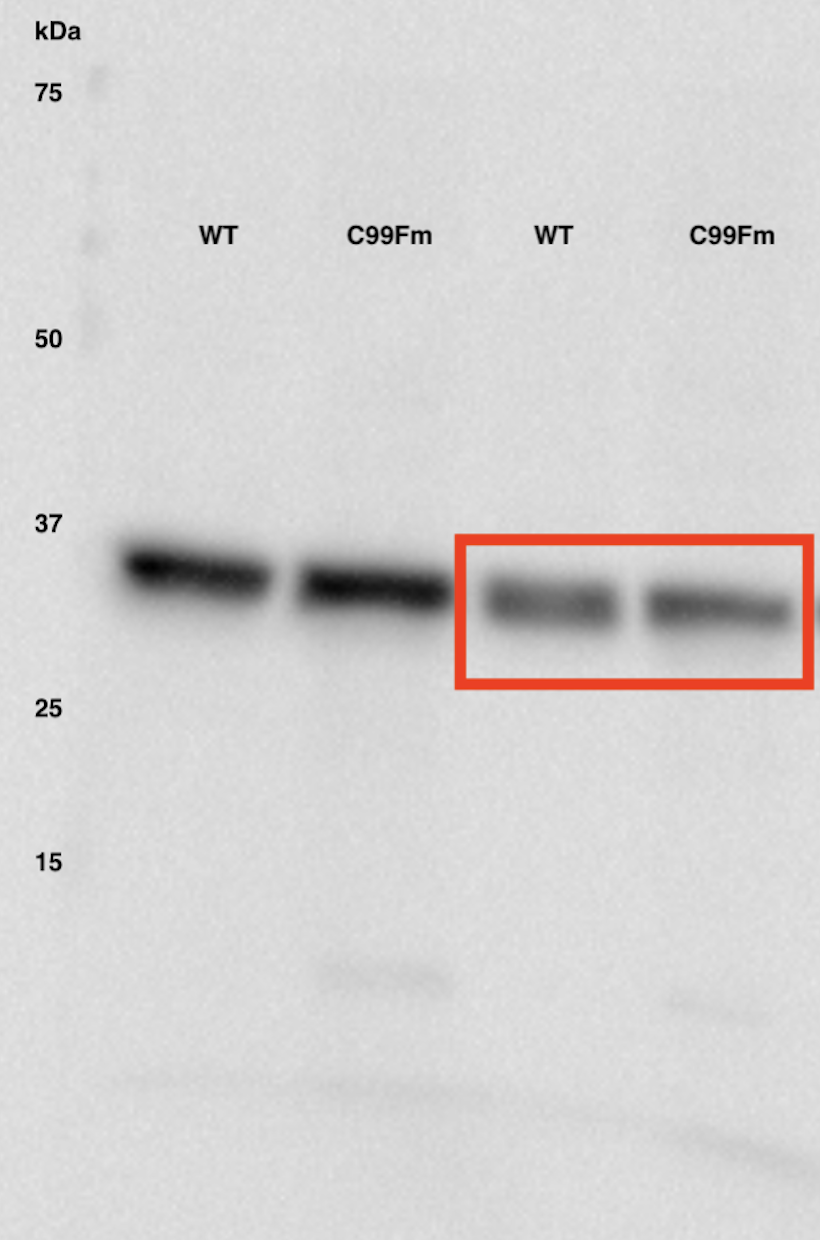

Supplement: Supplementary file 11 — Source Data Fig. 7 [file 44319_2024_82_MOESM11_ESM.zip › Figure 7/7A/western GAPDH.png]

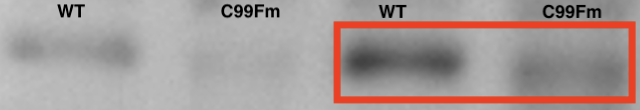

Supplement: Supplementary file 11 — Source Data Fig. 7 [file 44319_2024_82_MOESM11_ESM.zip › Figure 7/7A/western PHF6.tiff]

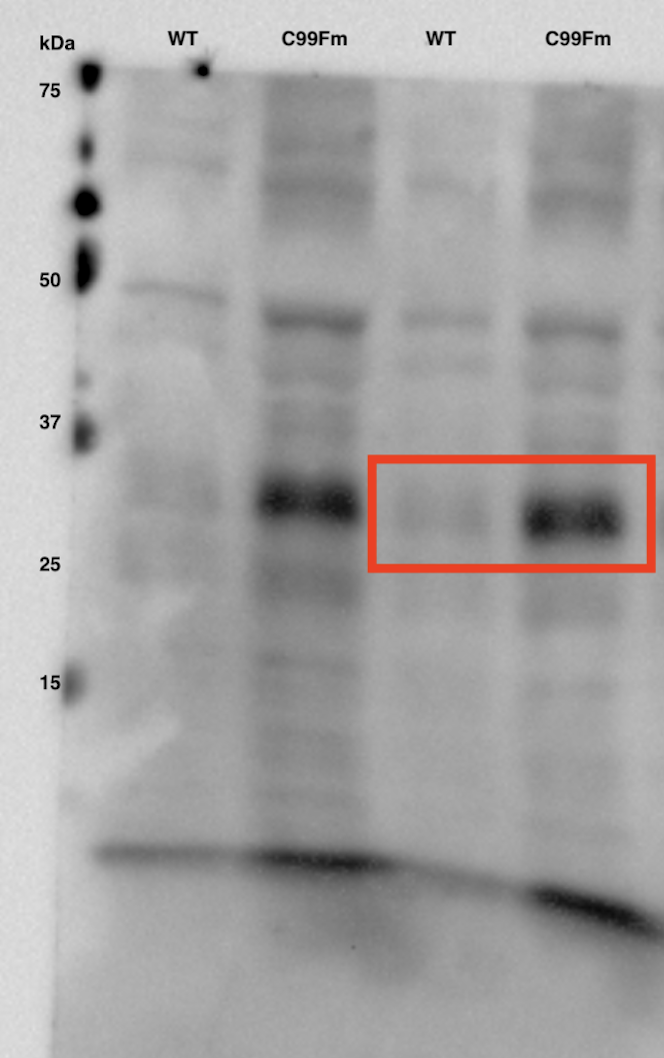

Supplement: Supplementary file 11 — Source Data Fig. 7 [file 44319_2024_82_MOESM11_ESM.zip › Figure 7/7A/western sox2.png]

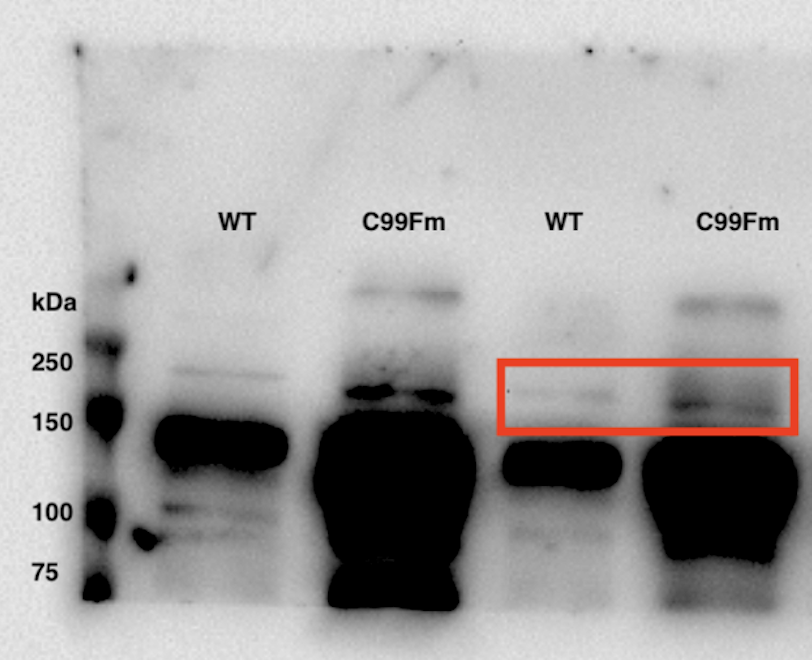

Supplement: Supplementary file 11 — Source Data Fig. 7 [file 44319_2024_82_MOESM11_ESM.zip › Figure 7/7A/western Nestin.png]

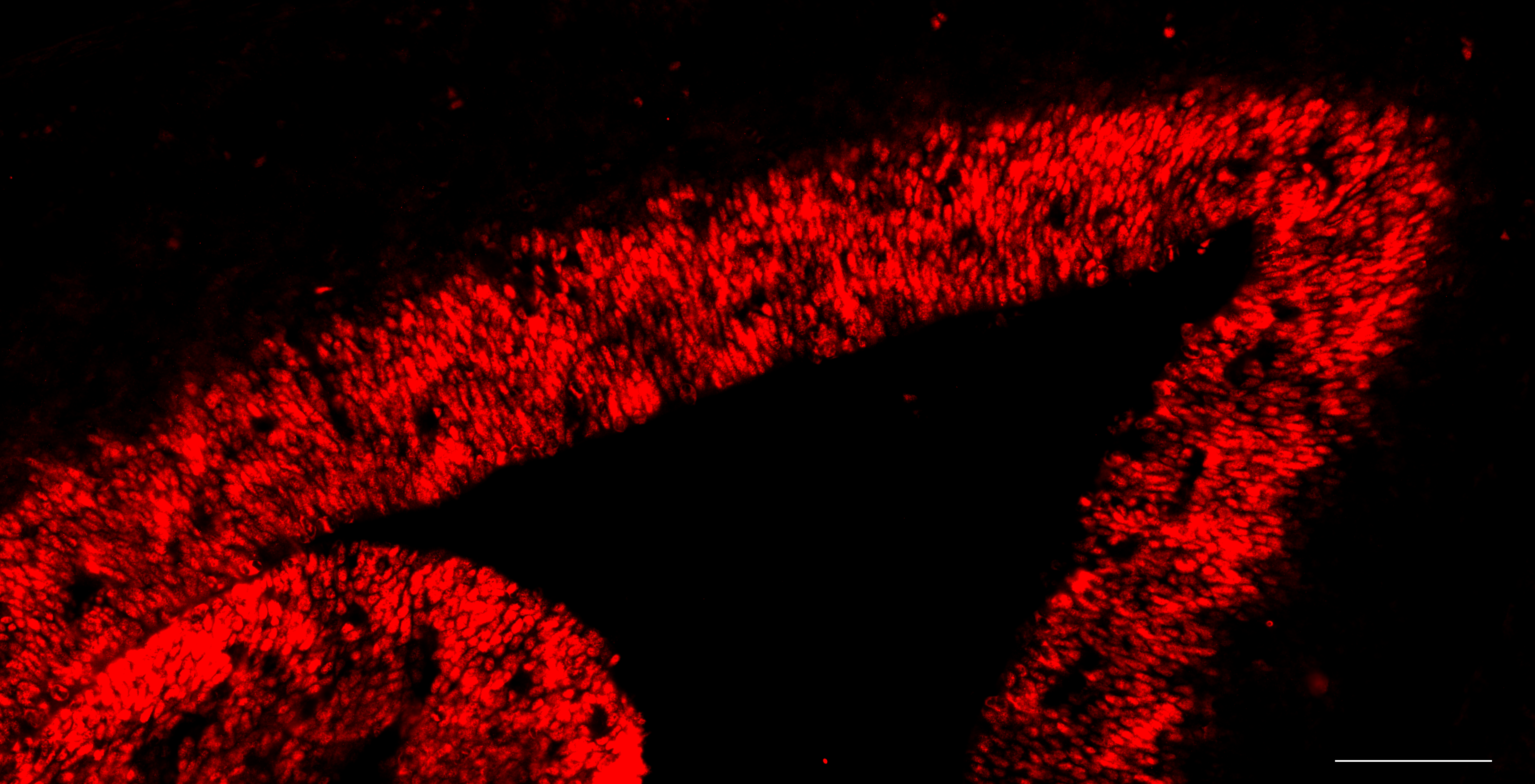

Supplement: Supplementary file 11 — Source Data Fig. 7 [file 44319_2024_82_MOESM11_ESM.zip › Figure 7/7F/7F WT full structure/wt sox2.tif]

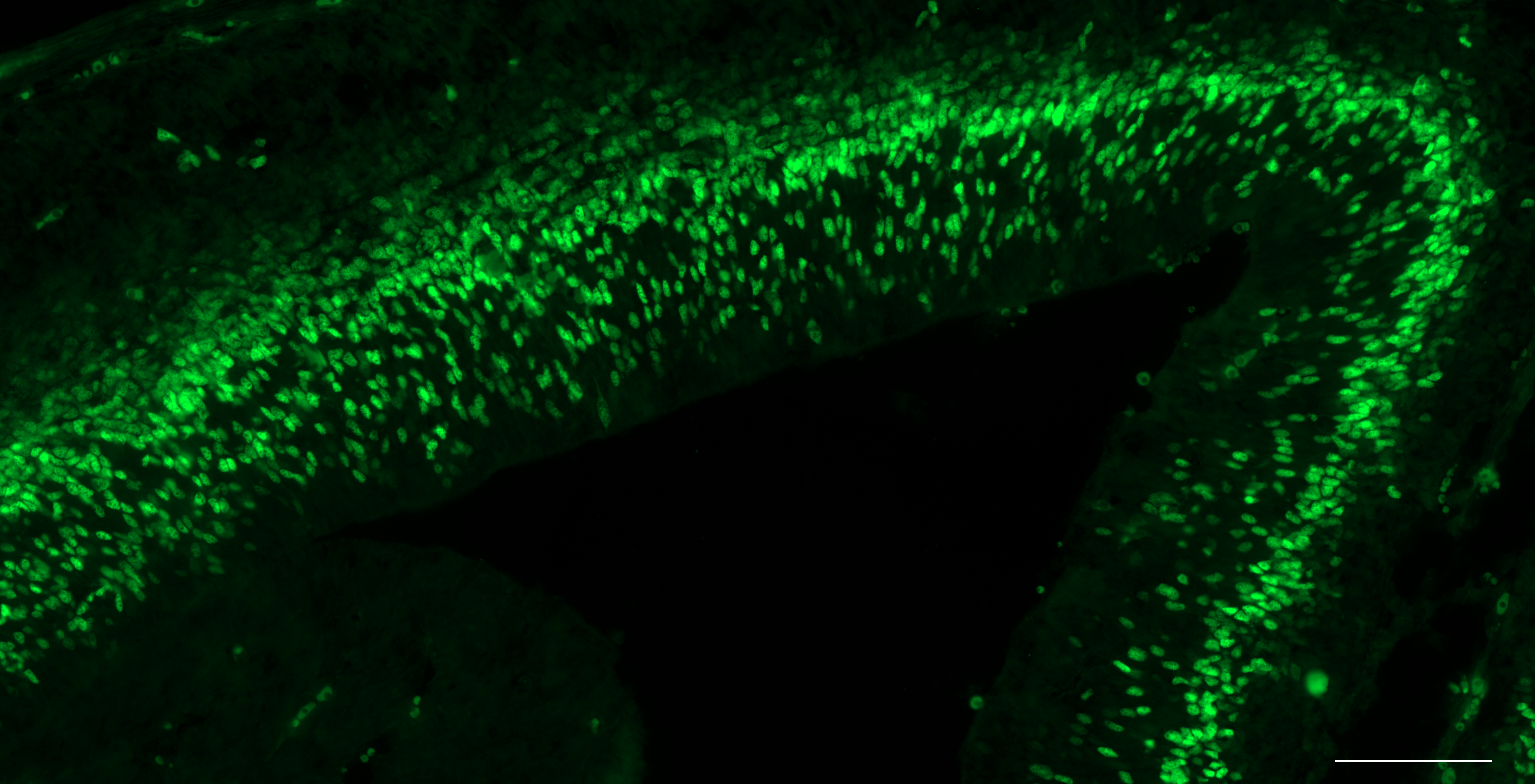

Supplement: Supplementary file 11 — Source Data Fig. 7 [file 44319_2024_82_MOESM11_ESM.zip › Figure 7/7F/7F WT full structure/wt tbr2.tif]

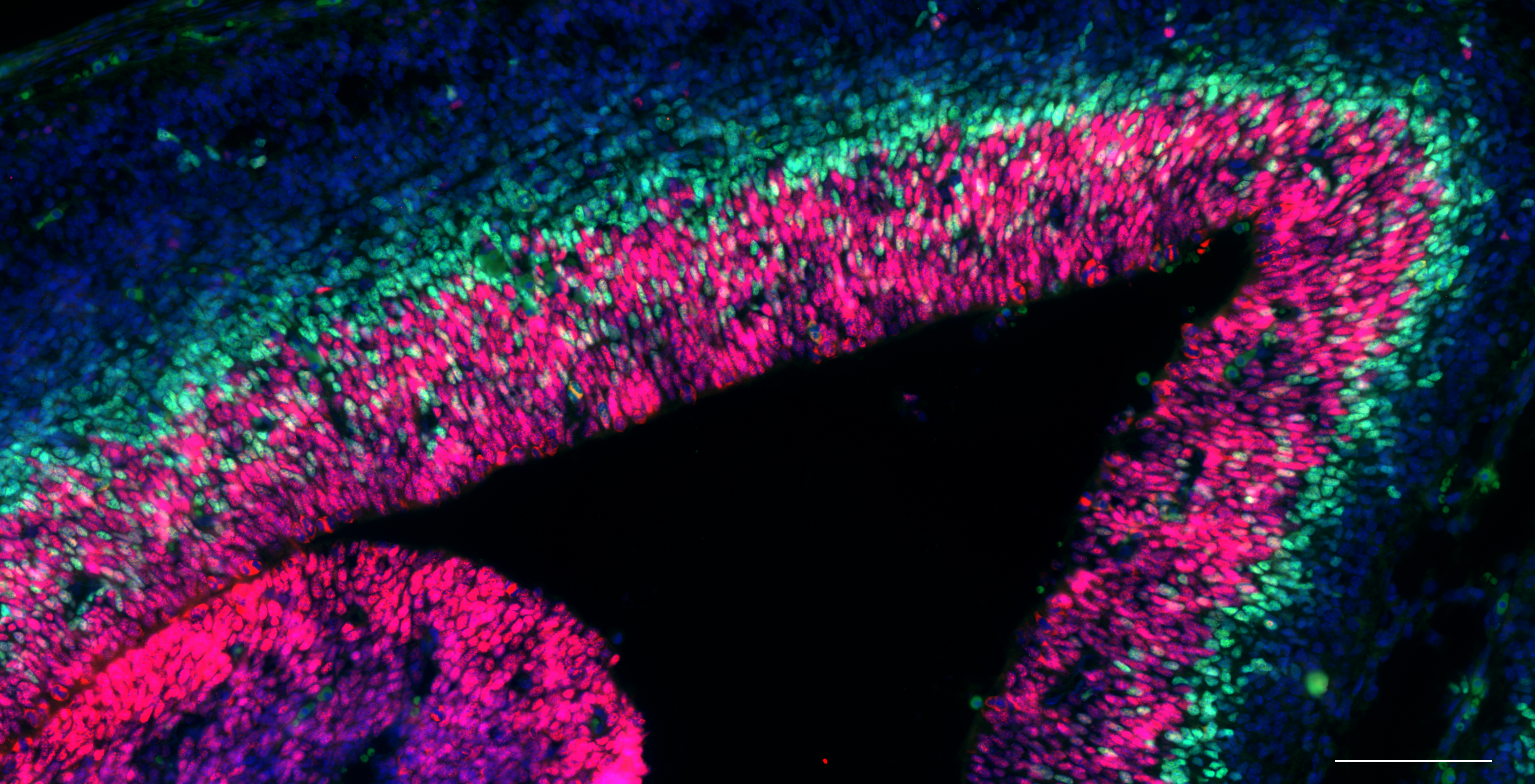

Supplement: Supplementary file 11 — Source Data Fig. 7 [file 44319_2024_82_MOESM11_ESM.zip › Figure 7/7F/7F WT full structure/wt merged.tif]

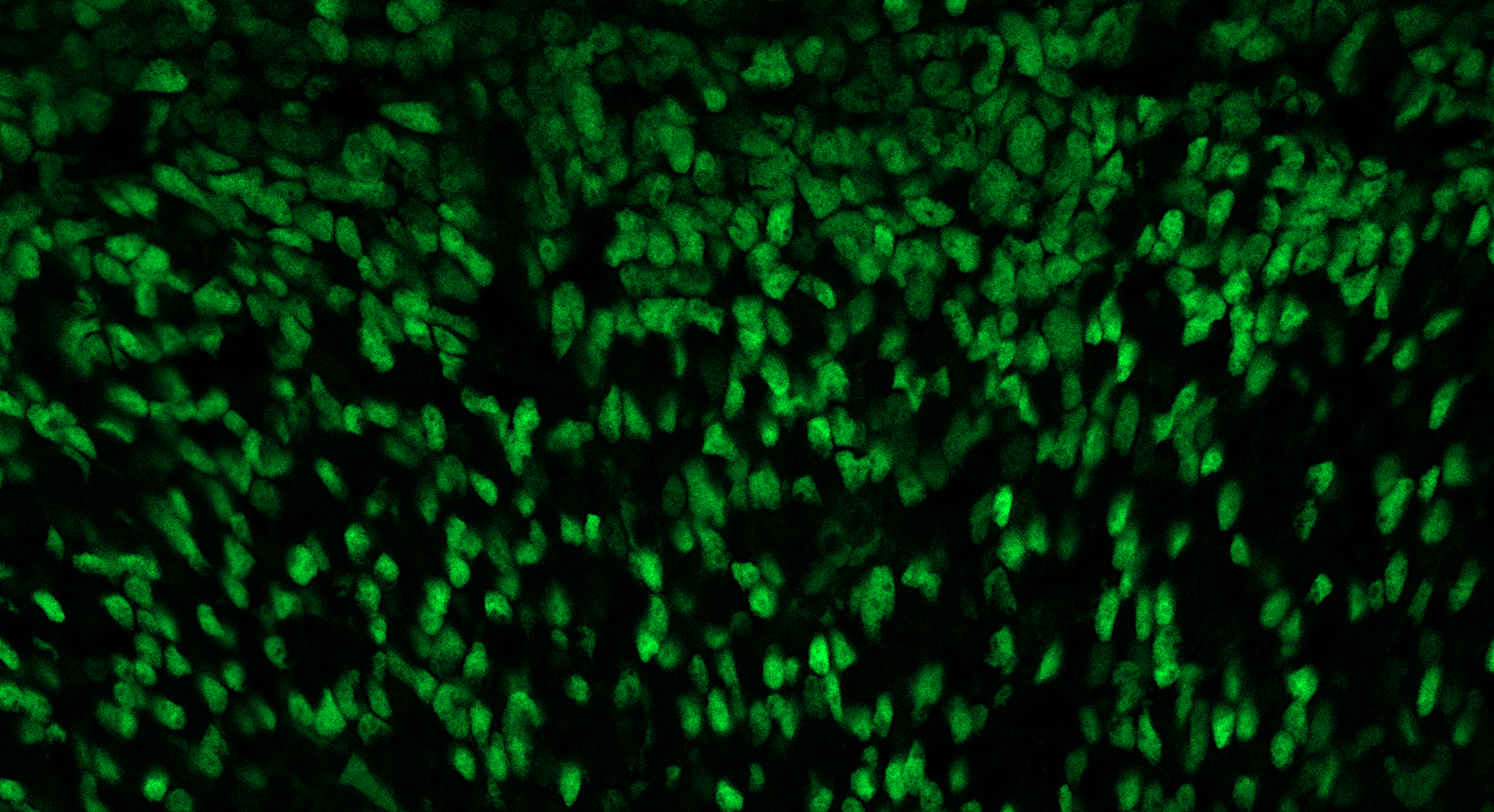

Supplement: Supplementary file 11 — Source Data Fig. 7 [file 44319_2024_82_MOESM11_ESM.zip › Figure 7/7F/7F WT ROI/wt ROI TBR2.tif]

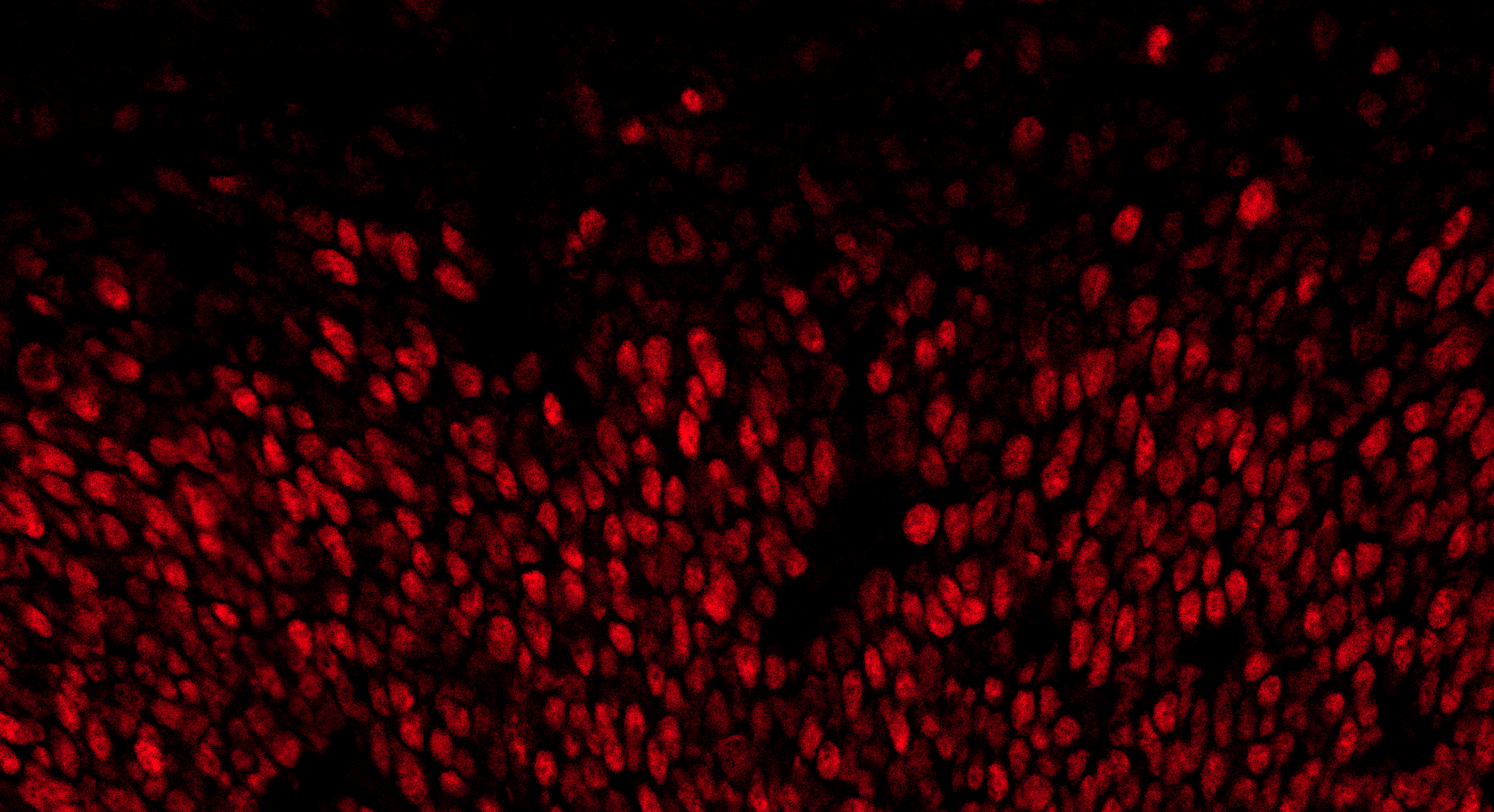

Supplement: Supplementary file 11 — Source Data Fig. 7 [file 44319_2024_82_MOESM11_ESM.zip › Figure 7/7F/7F WT ROI/wt ROI SOX2.tif]

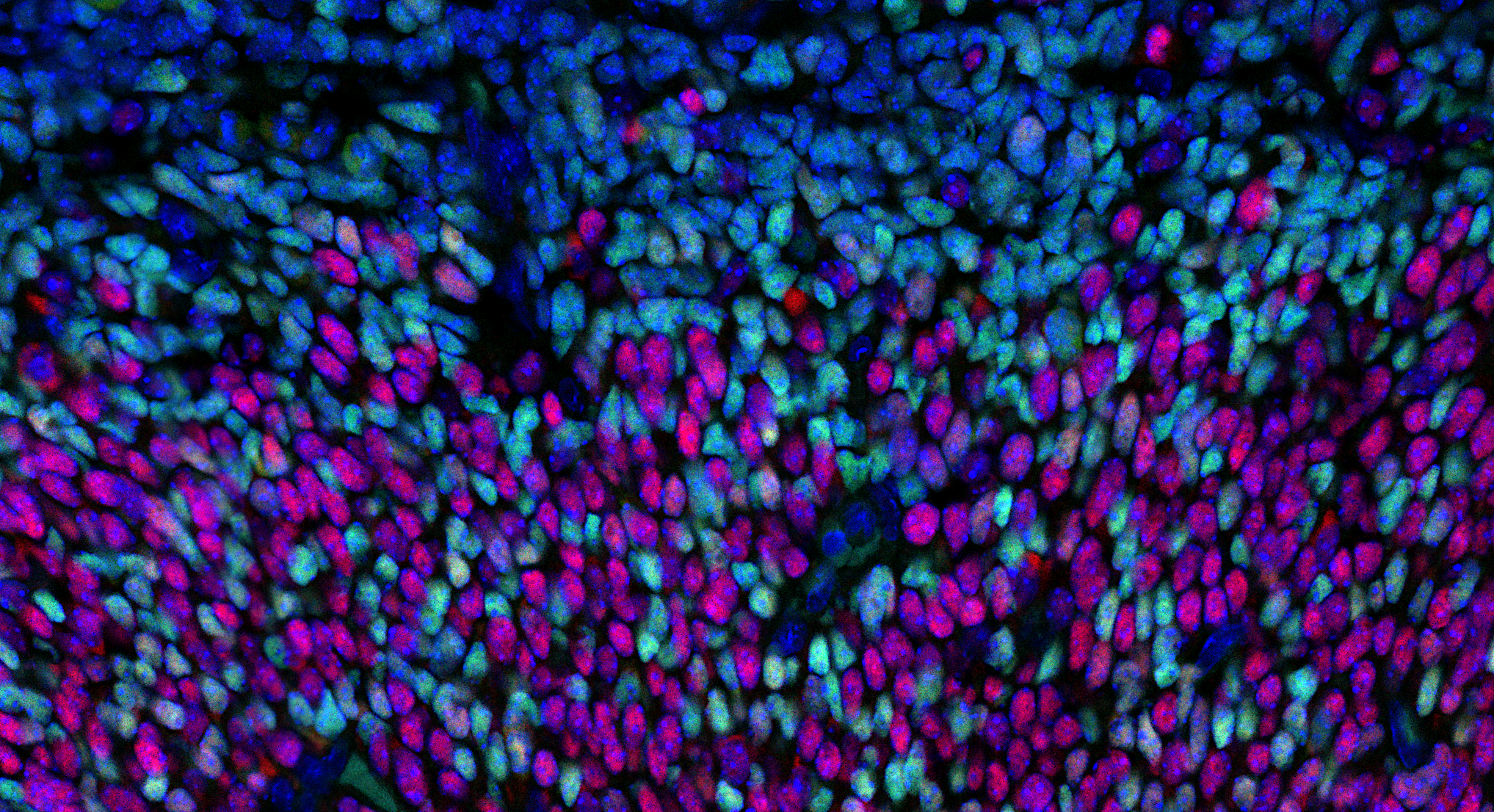

Supplement: Supplementary file 11 — Source Data Fig. 7 [file 44319_2024_82_MOESM11_ESM.zip › Figure 7/7F/7F WT ROI/wt ROI merged.tif]

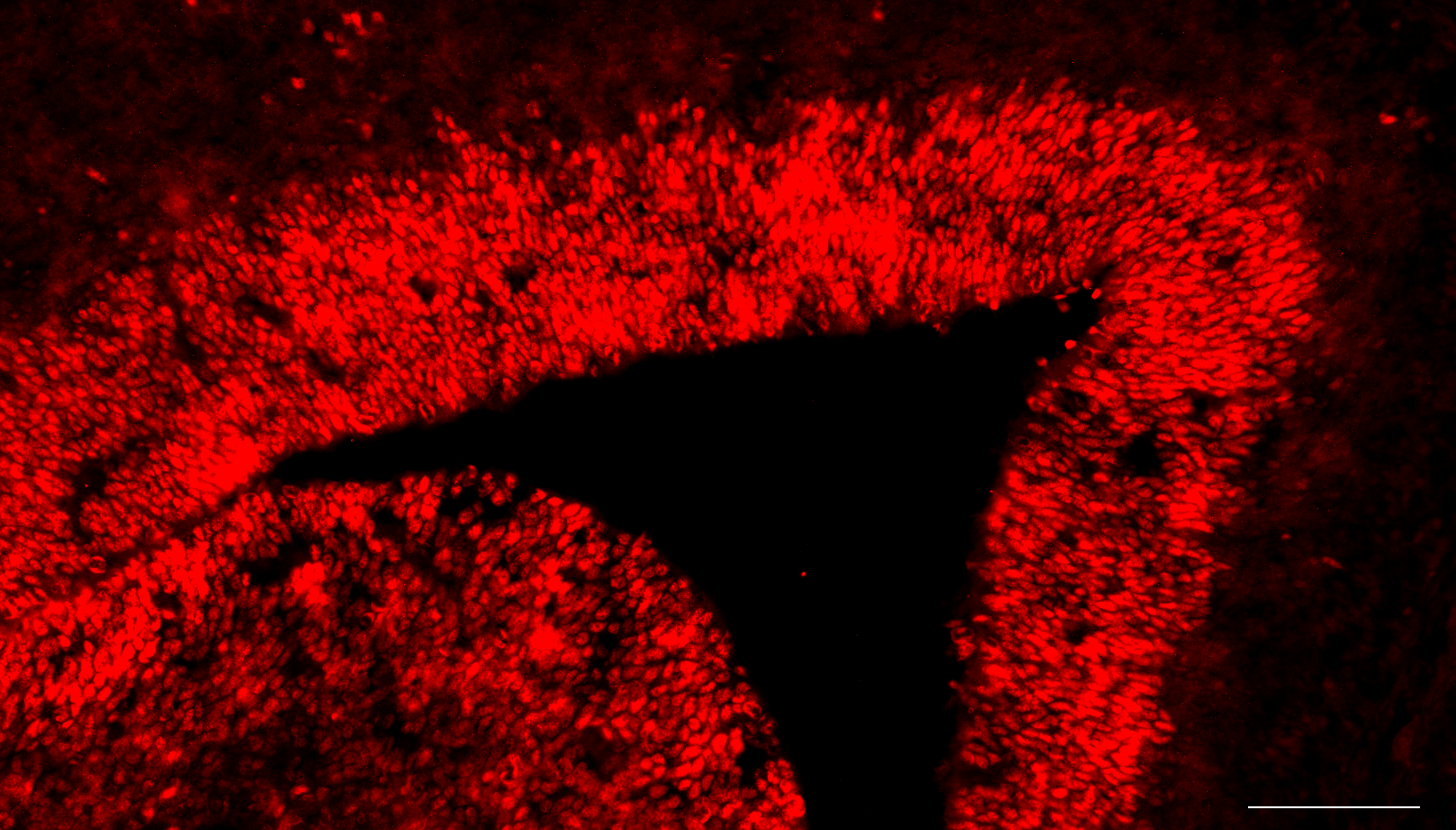

Supplement: Supplementary file 11 — Source Data Fig. 7 [file 44319_2024_82_MOESM11_ESM.zip › Figure 7/7F/7F R342X full structure/r342x sox2.tif]

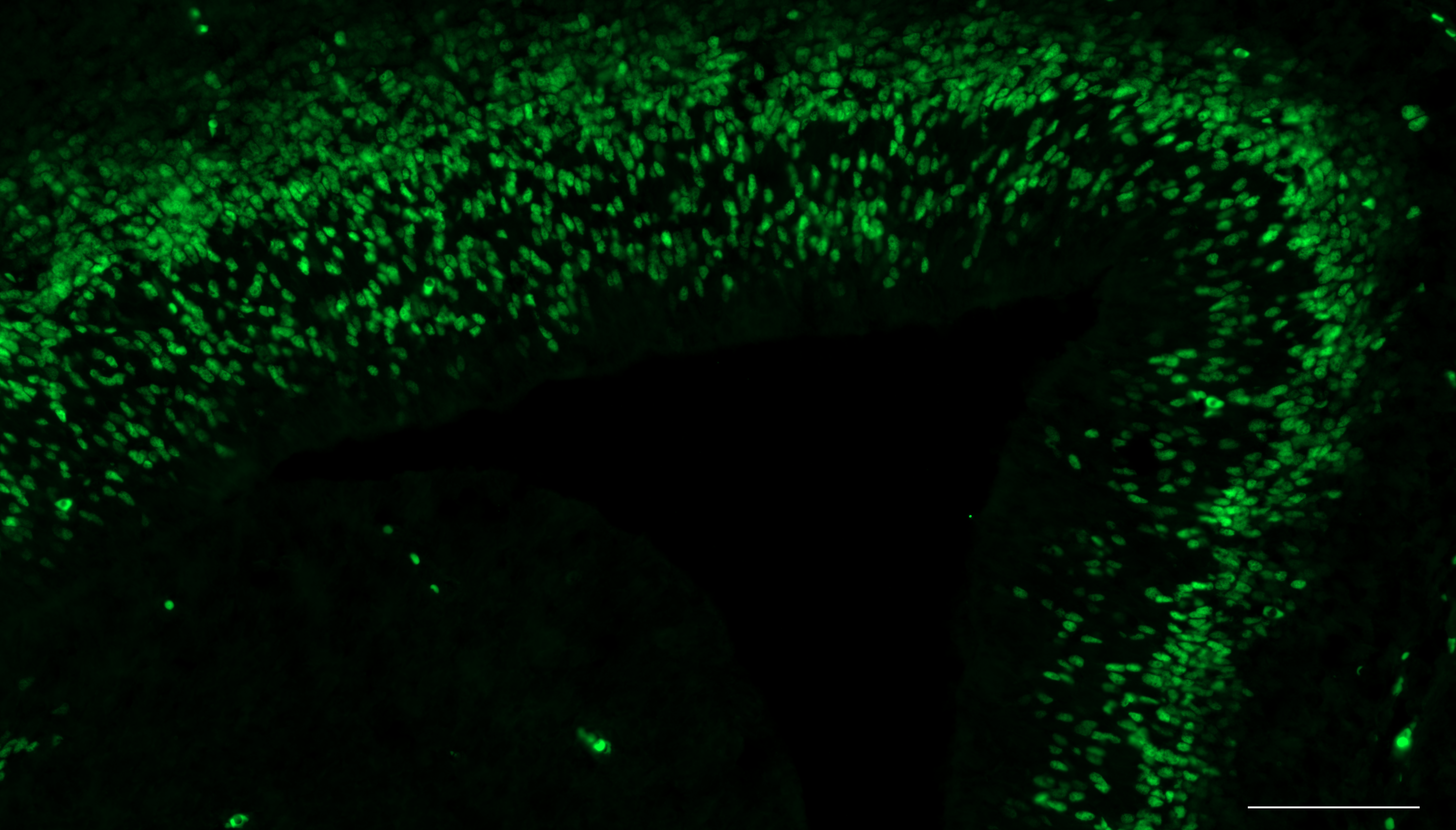

Supplement: Supplementary file 11 — Source Data Fig. 7 [file 44319_2024_82_MOESM11_ESM.zip › Figure 7/7F/7F R342X full structure/r342x tbr2.tif]

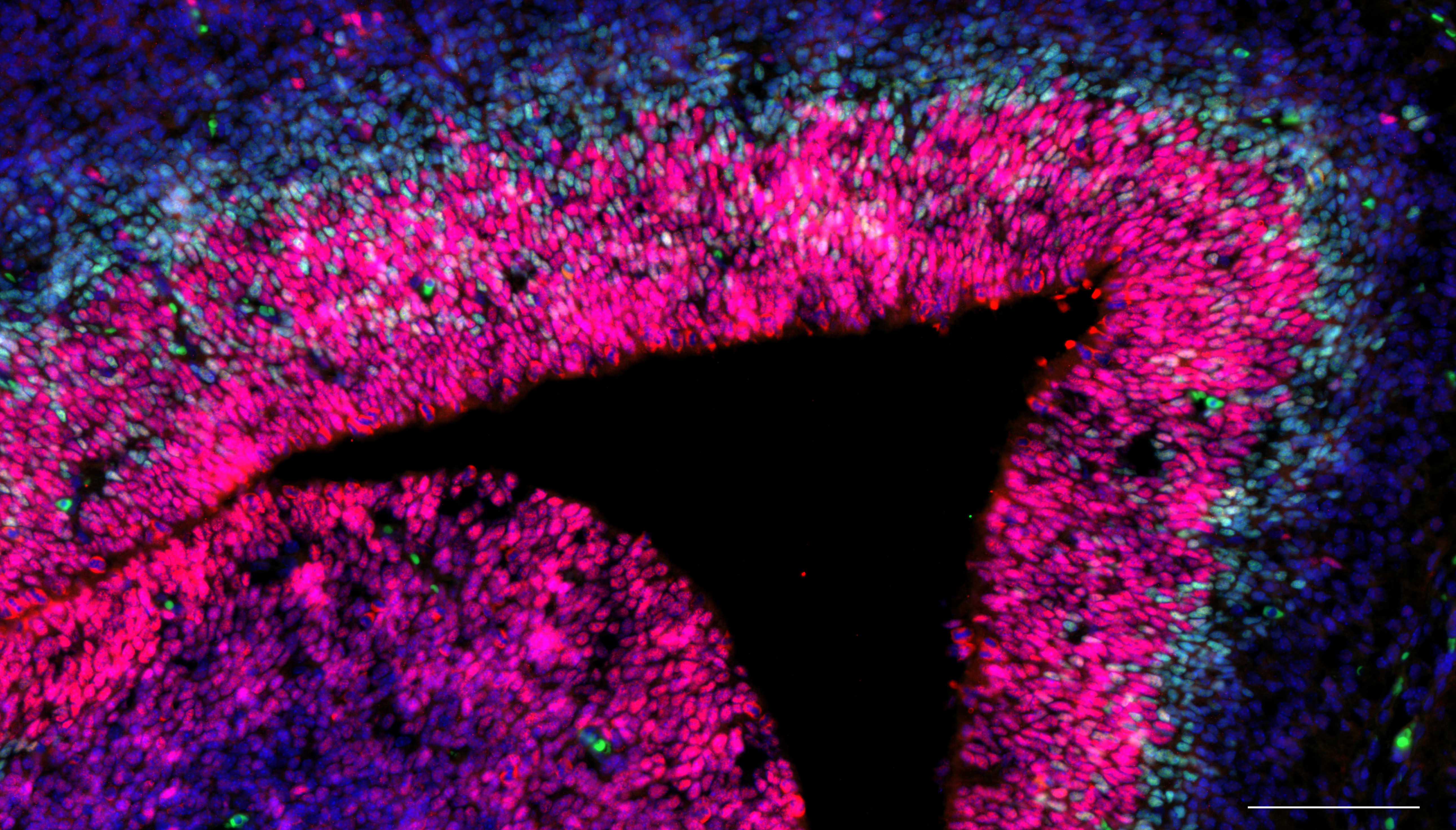

Supplement: Supplementary file 11 — Source Data Fig. 7 [file 44319_2024_82_MOESM11_ESM.zip › Figure 7/7F/7F R342X full structure/r342x merged.tif]

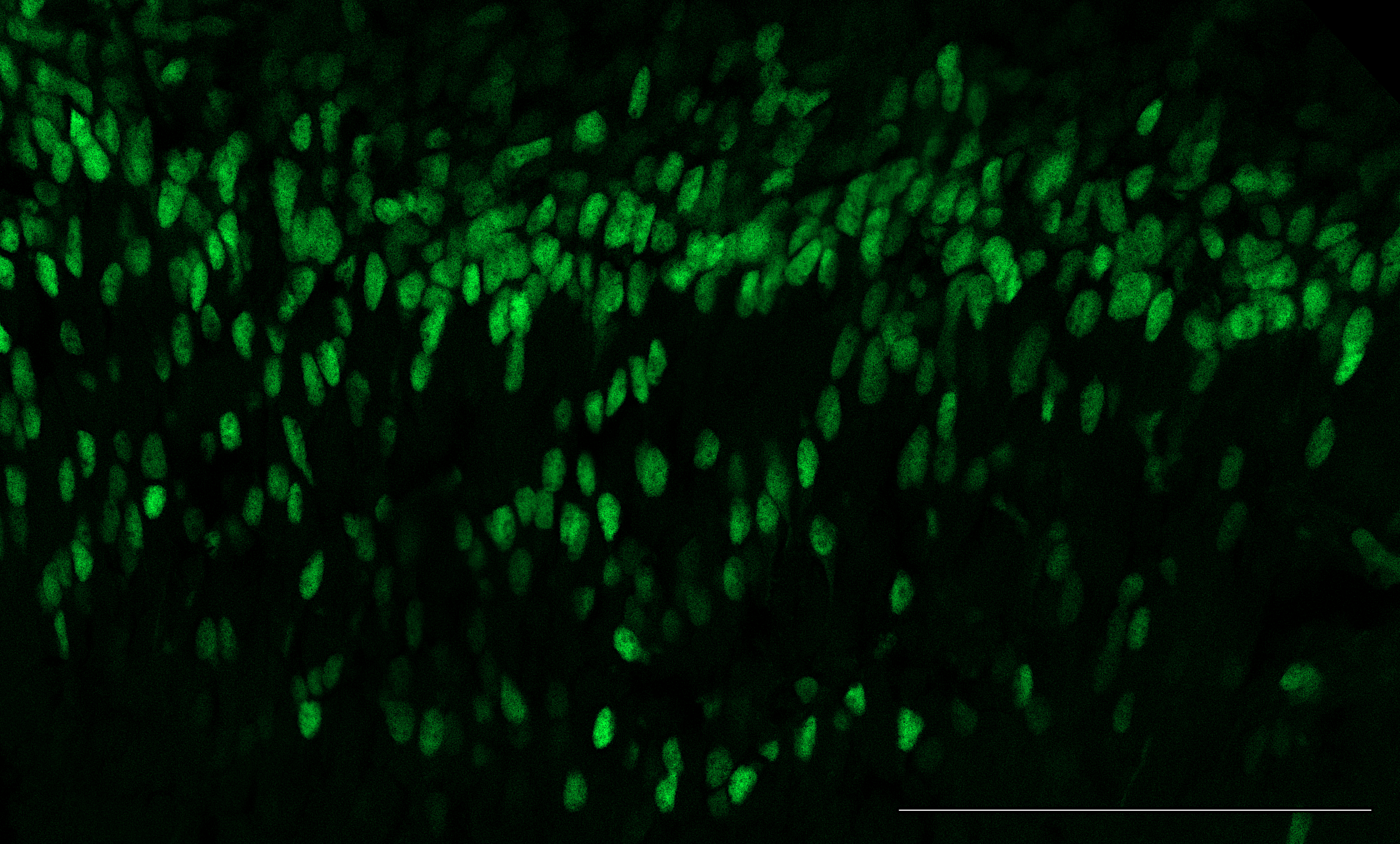

Supplement: Supplementary file 11 — Source Data Fig. 7 [file 44319_2024_82_MOESM11_ESM.zip › Figure 7/7F/7F R342X ROI/r342x tbr2 roi.tif]

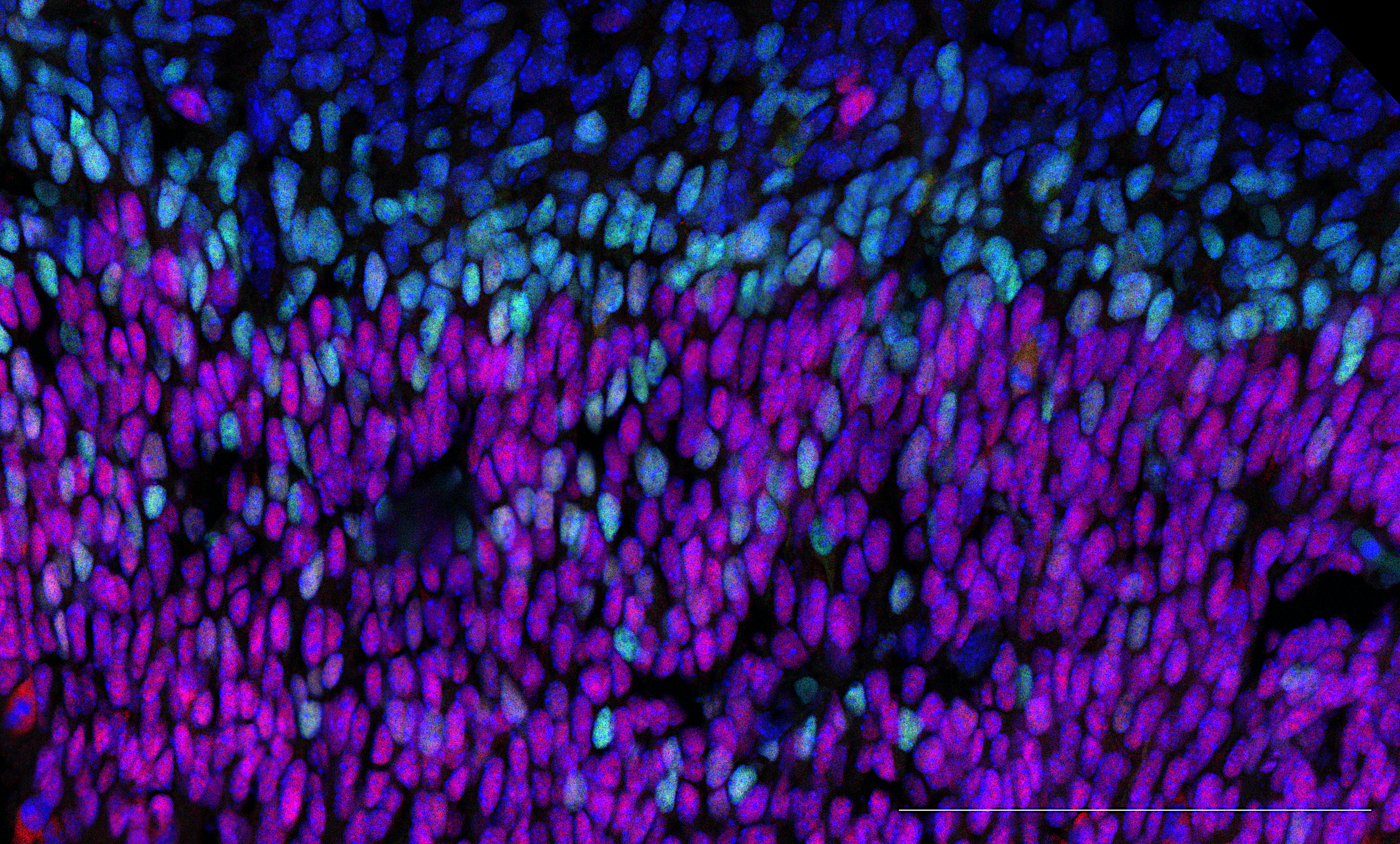

Supplement: Supplementary file 11 — Source Data Fig. 7 [file 44319_2024_82_MOESM11_ESM.zip › Figure 7/7F/7F R342X ROI/r342x merged roi.tif]

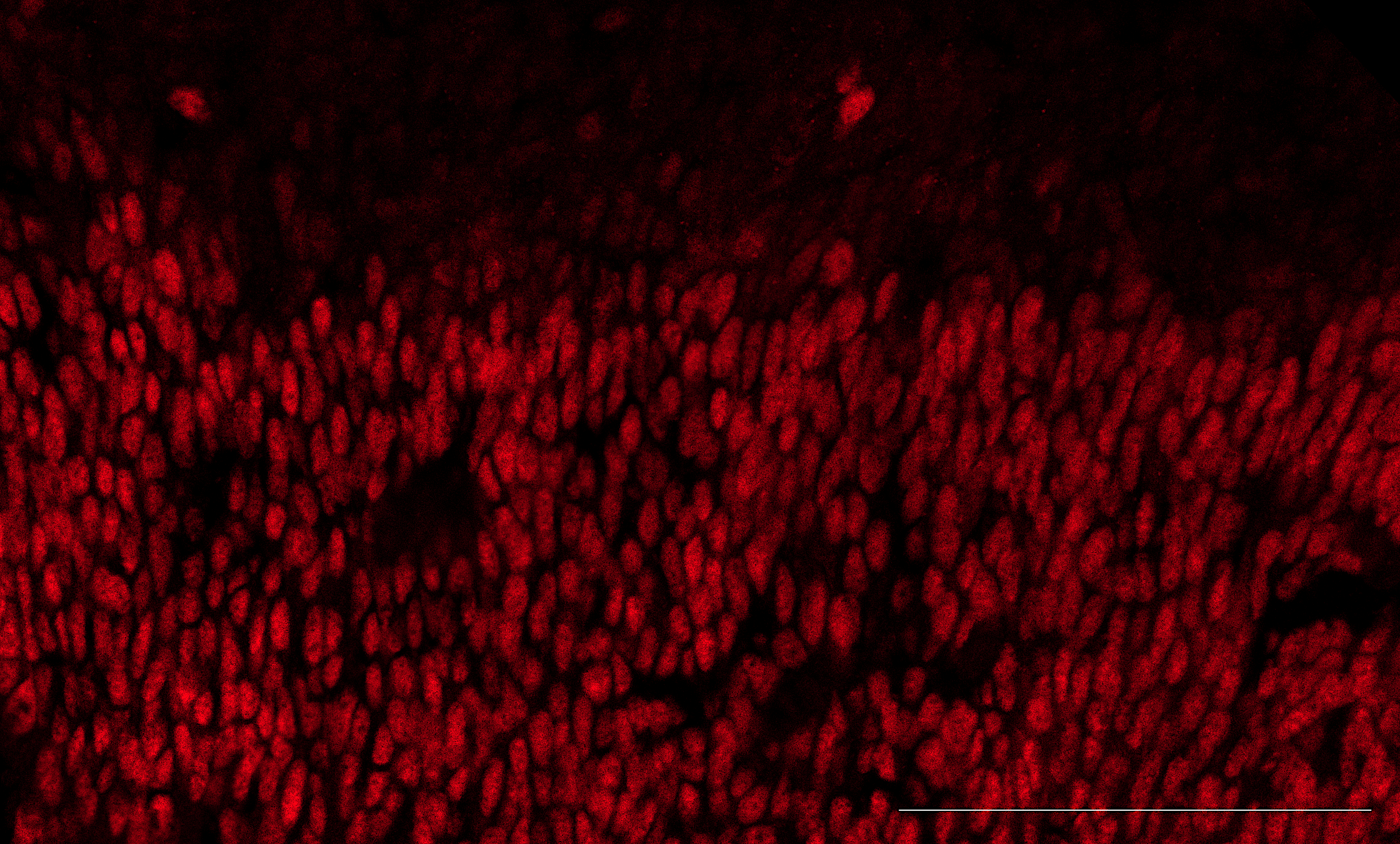

Supplement: Supplementary file 11 — Source Data Fig. 7 [file 44319_2024_82_MOESM11_ESM.zip › Figure 7/7F/7F R342X ROI/r342x sox2 roi.tif]

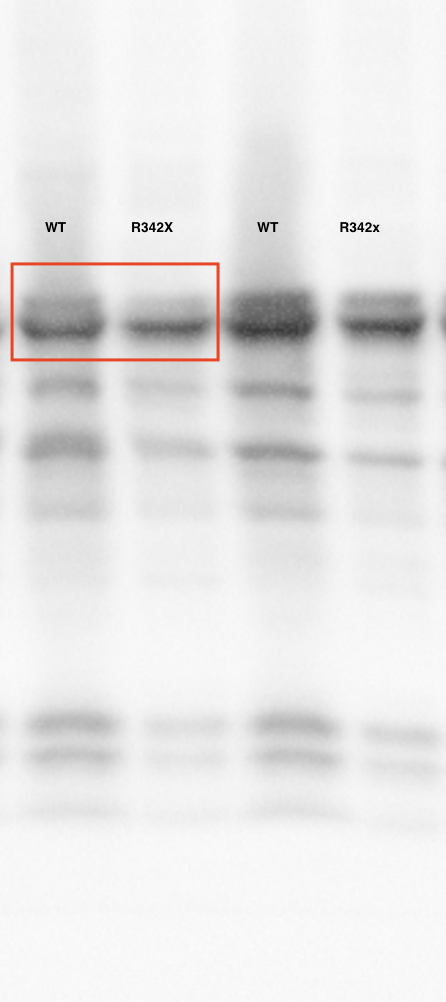

Supplement: Supplementary file 12 — Figure EV3 Source Data [file 44319_2024_82_MOESM12_ESM.zip › Figure EV3/EV3I/western DCX.png]

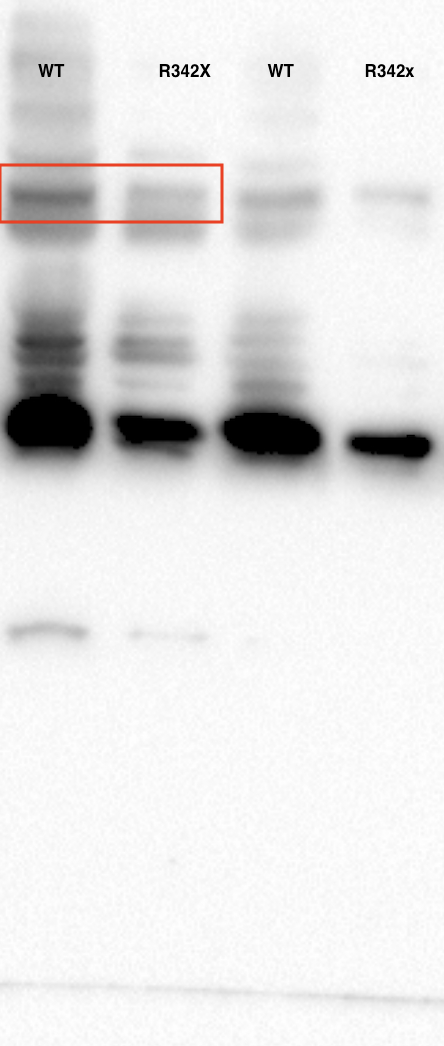

Supplement: Supplementary file 12 — Figure EV3 Source Data [file 44319_2024_82_MOESM12_ESM.zip › Figure EV3/EV3I/western GFAP.png]

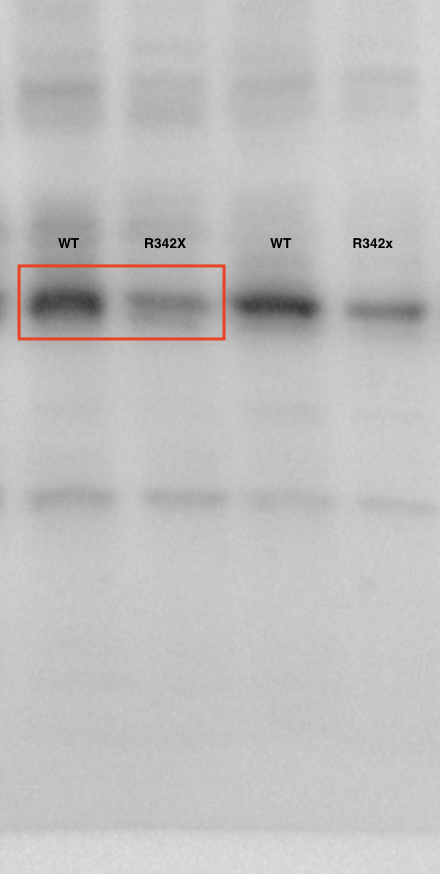

Supplement: Supplementary file 12 — Figure EV3 Source Data [file 44319_2024_82_MOESM12_ESM.zip › Figure EV3/EV3I/western OLIG2.png]

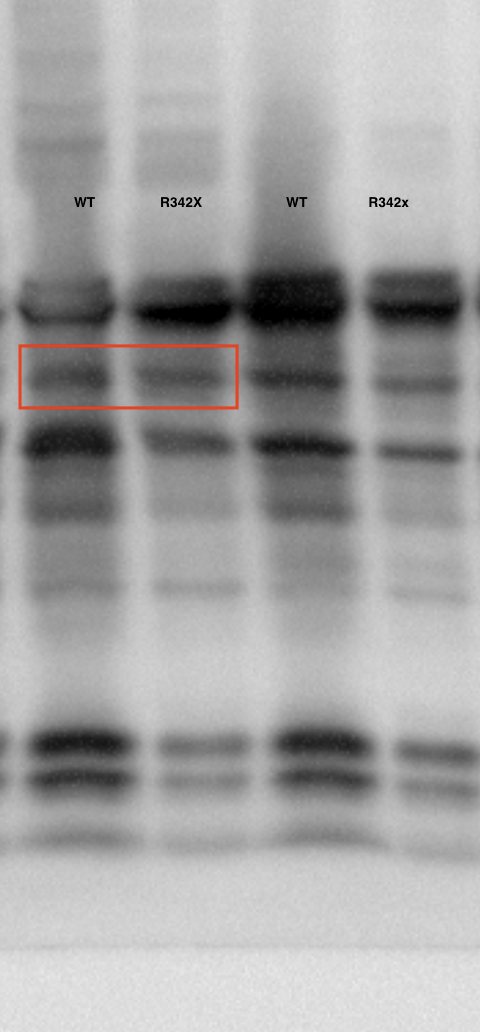

Supplement: Supplementary file 12 — Figure EV3 Source Data [file 44319_2024_82_MOESM12_ESM.zip › Figure EV3/EV3I/western GAPDH.png]

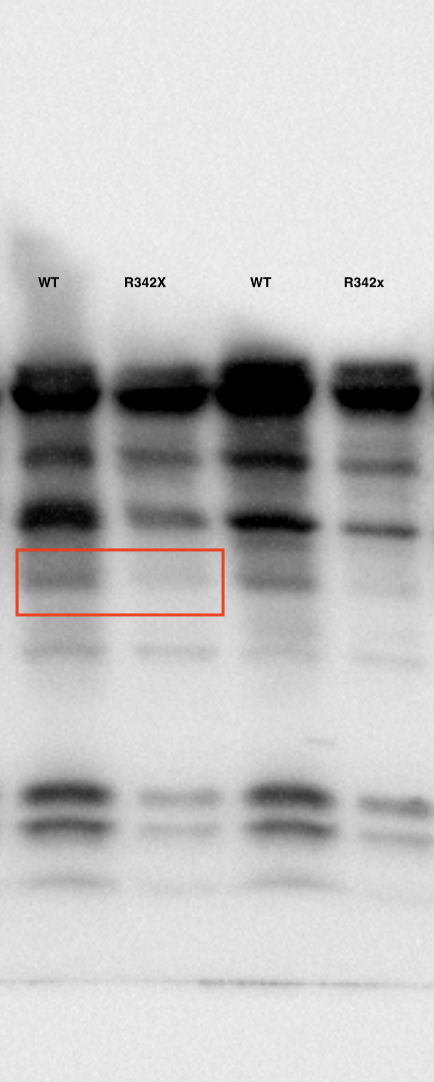

Supplement: Supplementary file 12 — Figure EV3 Source Data [file 44319_2024_82_MOESM12_ESM.zip › Figure EV3/EV3I/western ASCL1.png]

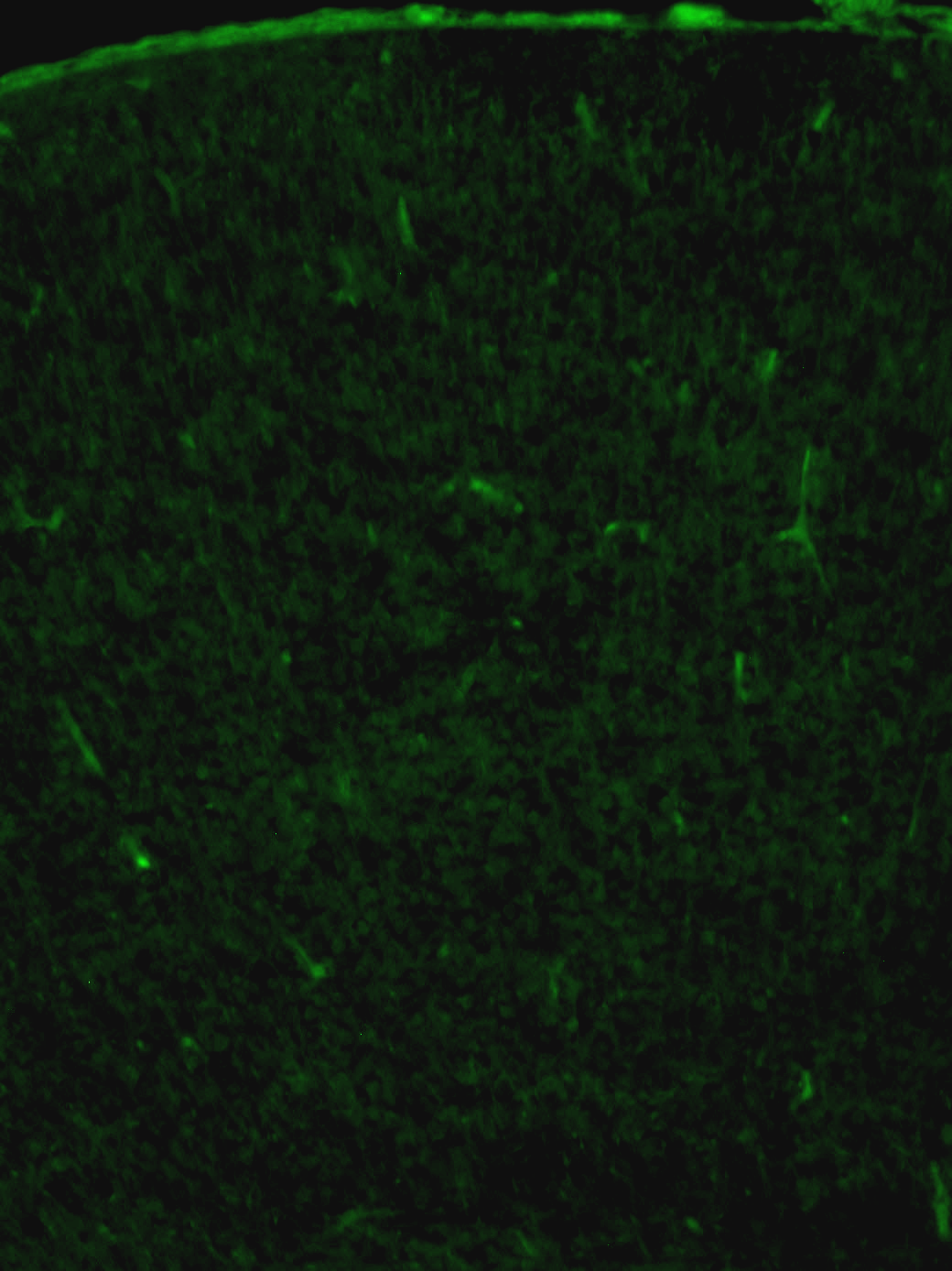

Supplement: Supplementary file 12 — Figure EV3 Source Data [file 44319_2024_82_MOESM12_ESM.zip › Figure EV3/EV3A/Phf6 KO/PHF6.tif]

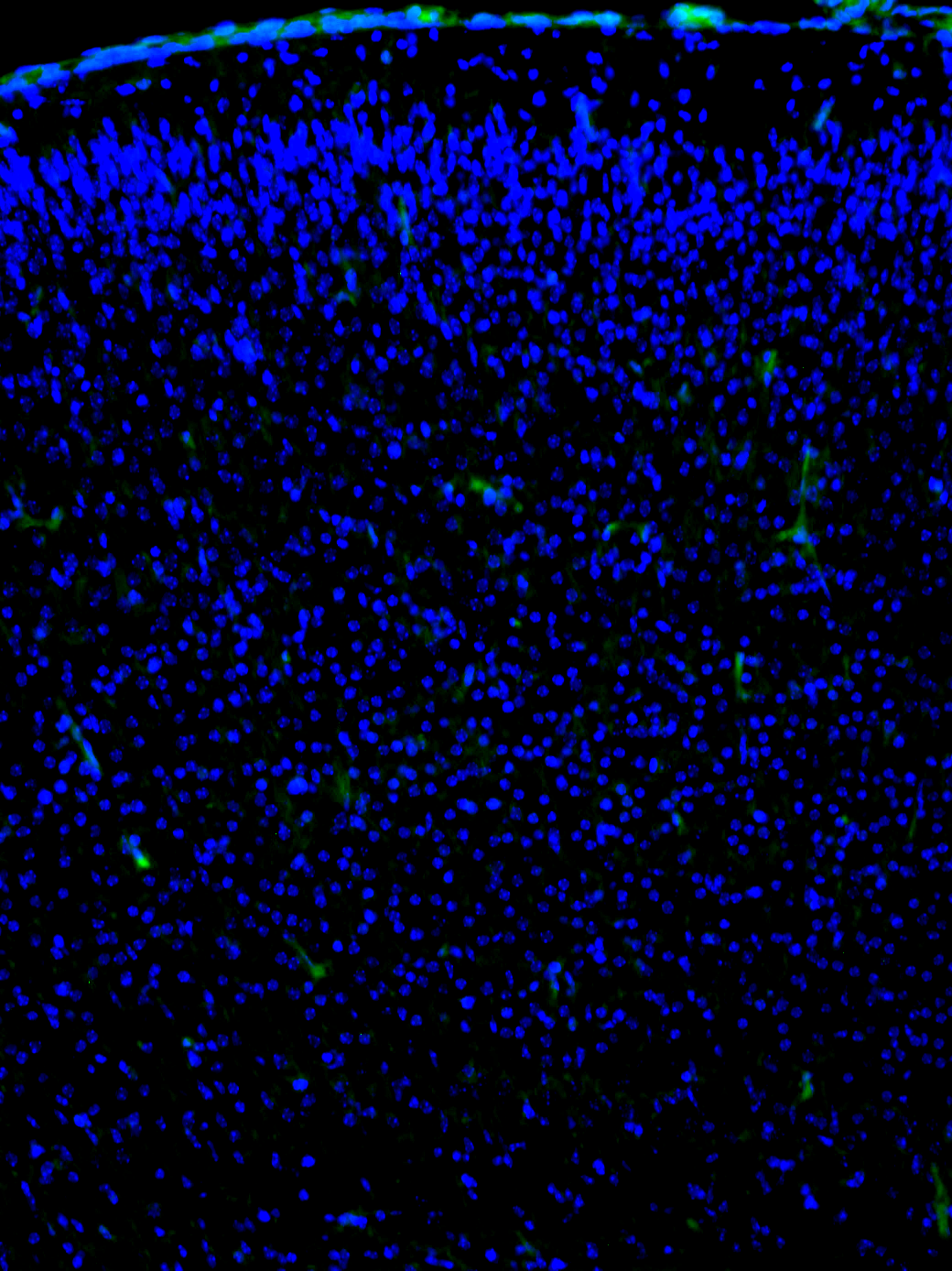

Supplement: Supplementary file 12 — Figure EV3 Source Data [file 44319_2024_82_MOESM12_ESM.zip › Figure EV3/EV3A/Phf6 KO/Merged.tif]

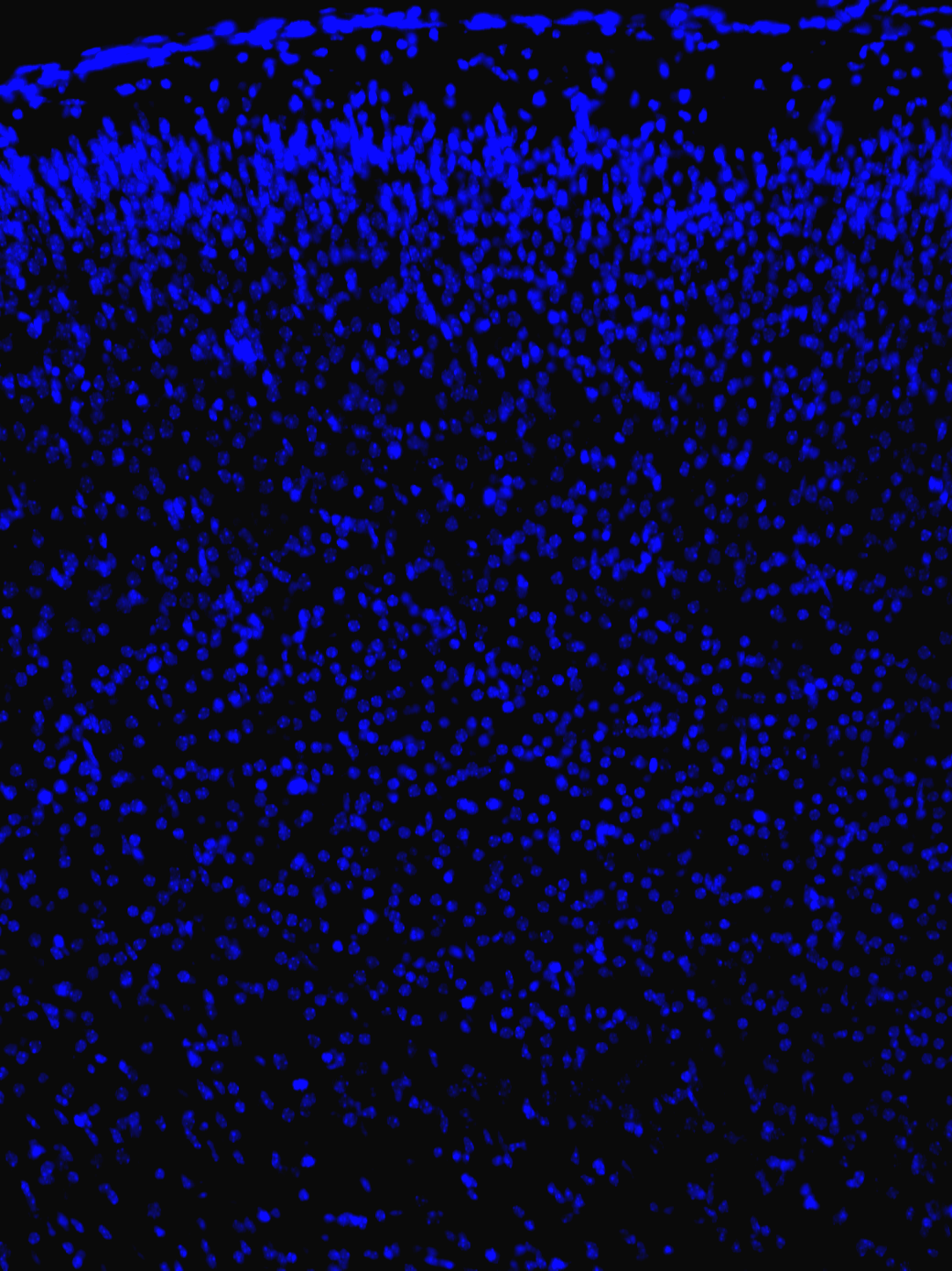

Supplement: Supplementary file 12 — Figure EV3 Source Data [file 44319_2024_82_MOESM12_ESM.zip › Figure EV3/EV3A/Phf6 KO/Hoechst.tif]

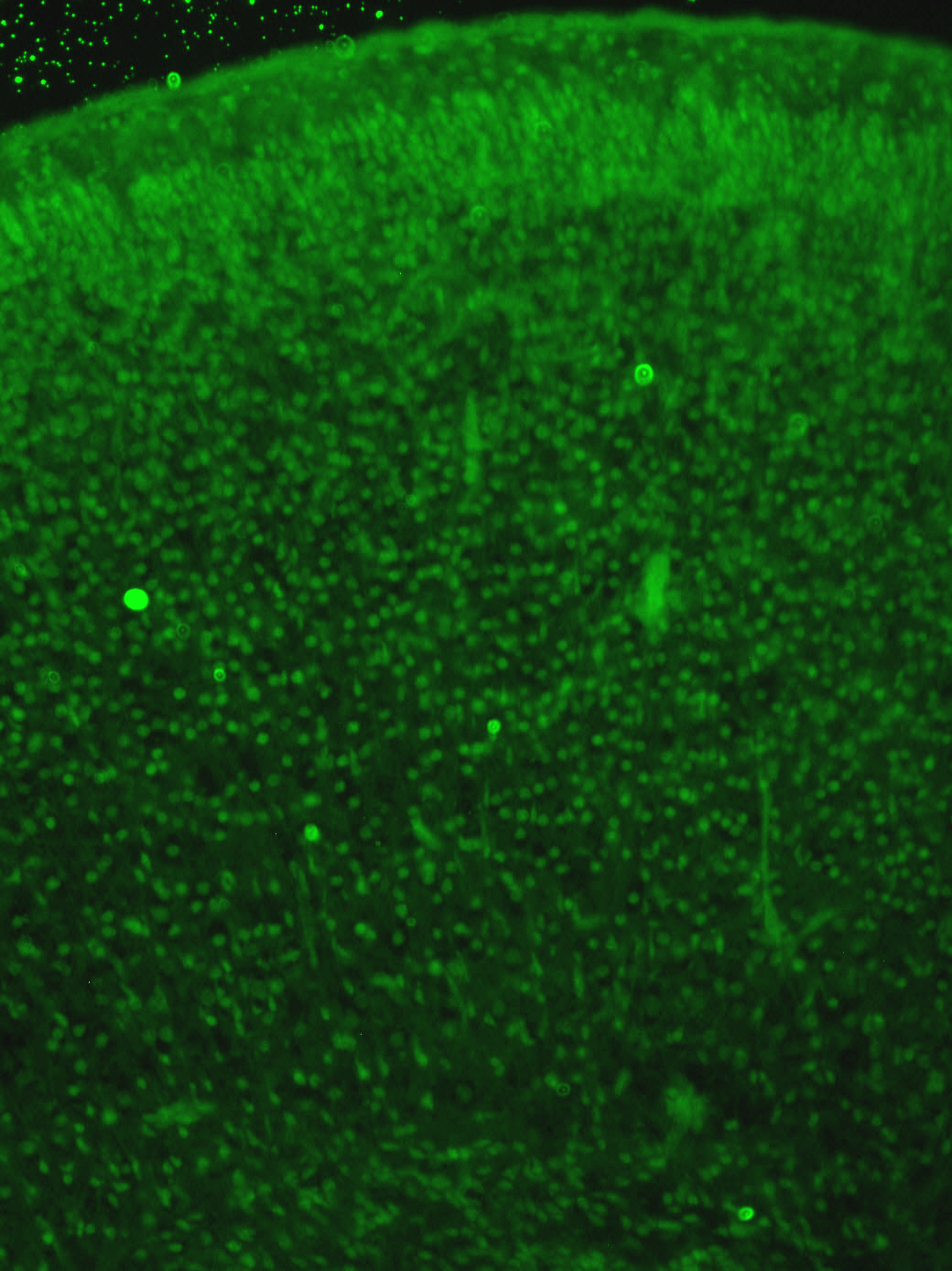

Supplement: Supplementary file 12 — Figure EV3 Source Data [file 44319_2024_82_MOESM12_ESM.zip › Figure EV3/EV3A/Ctl/PHF6.tif]

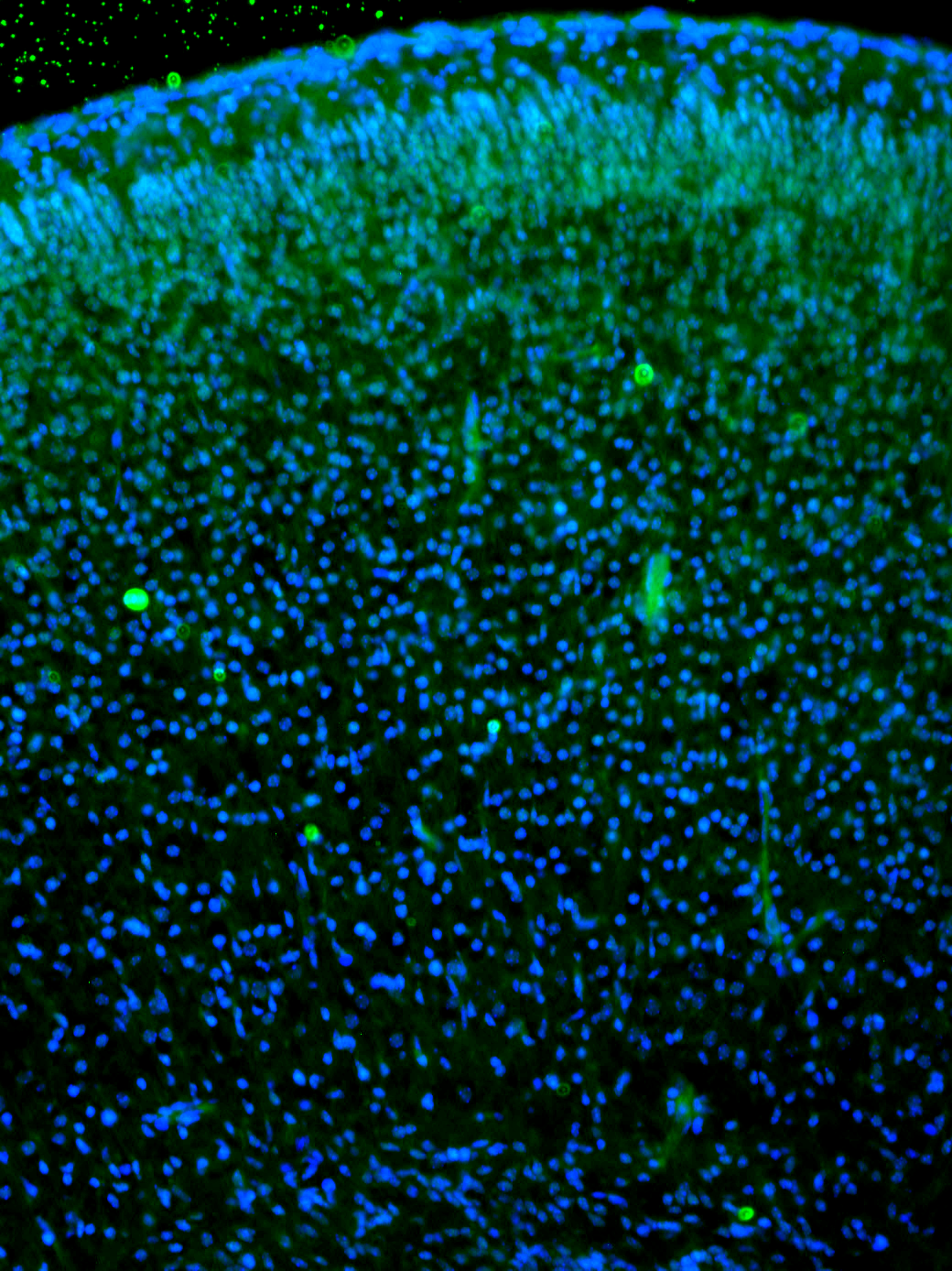

Supplement: Supplementary file 12 — Figure EV3 Source Data [file 44319_2024_82_MOESM12_ESM.zip › Figure EV3/EV3A/Ctl/merged.tif]

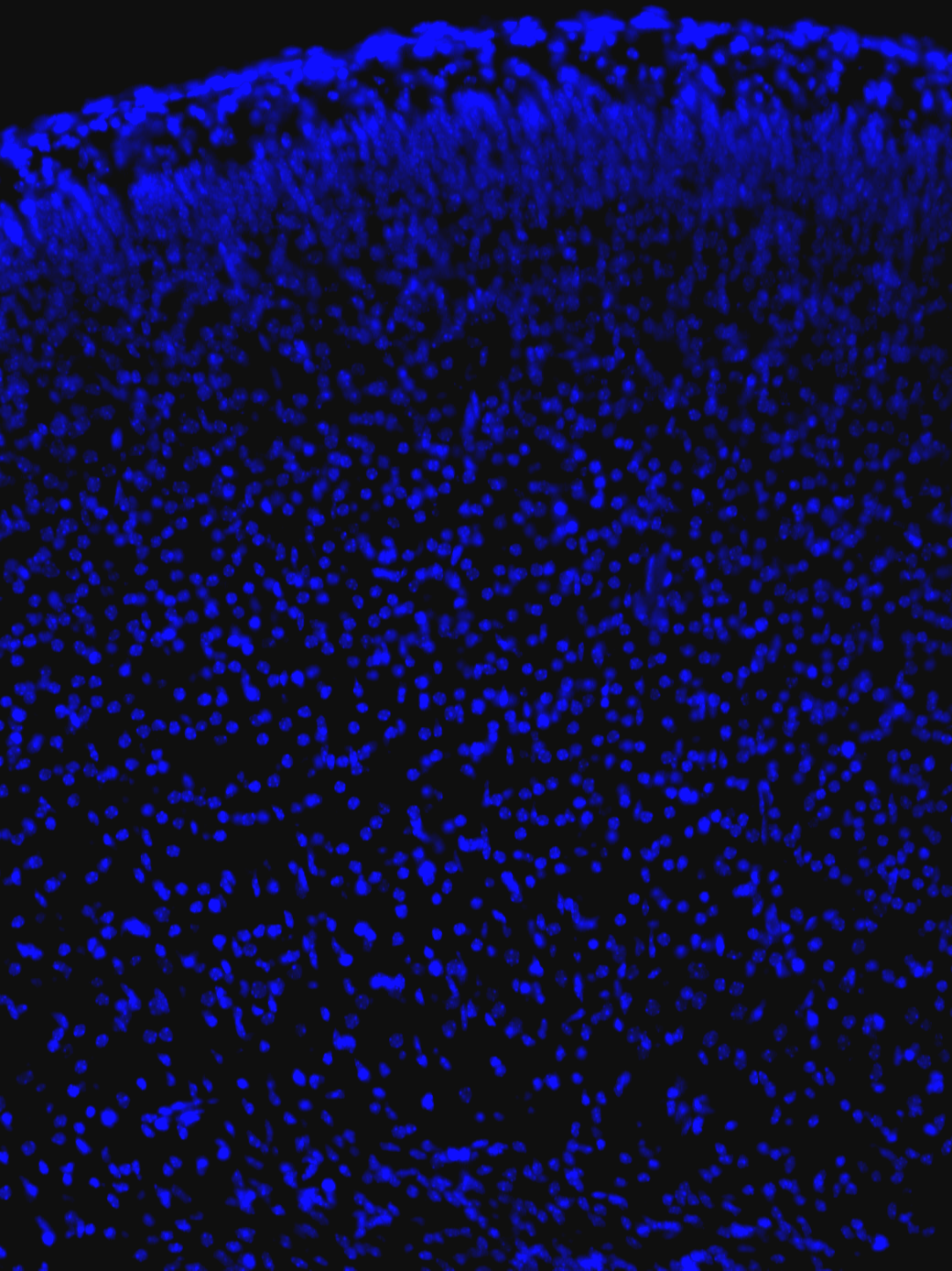

Supplement: Supplementary file 12 — Figure EV3 Source Data [file 44319_2024_82_MOESM12_ESM.zip › Figure EV3/EV3A/Ctl/Hoechst.tif]

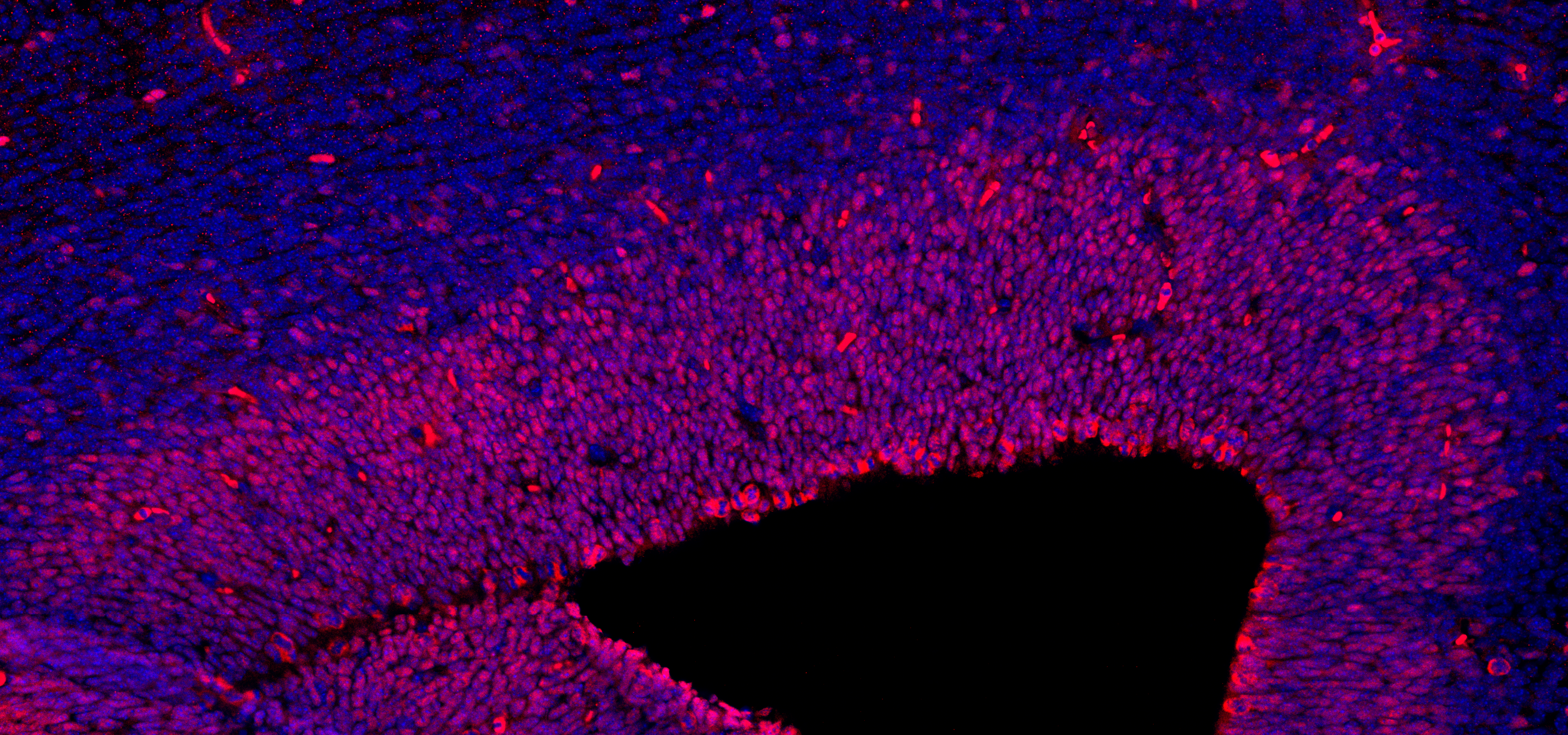

Supplement: Supplementary file 12 — Figure EV3 Source Data [file 44319_2024_82_MOESM12_ESM.zip › Figure EV3/EV3D/Phf6 ko/merged.tif]

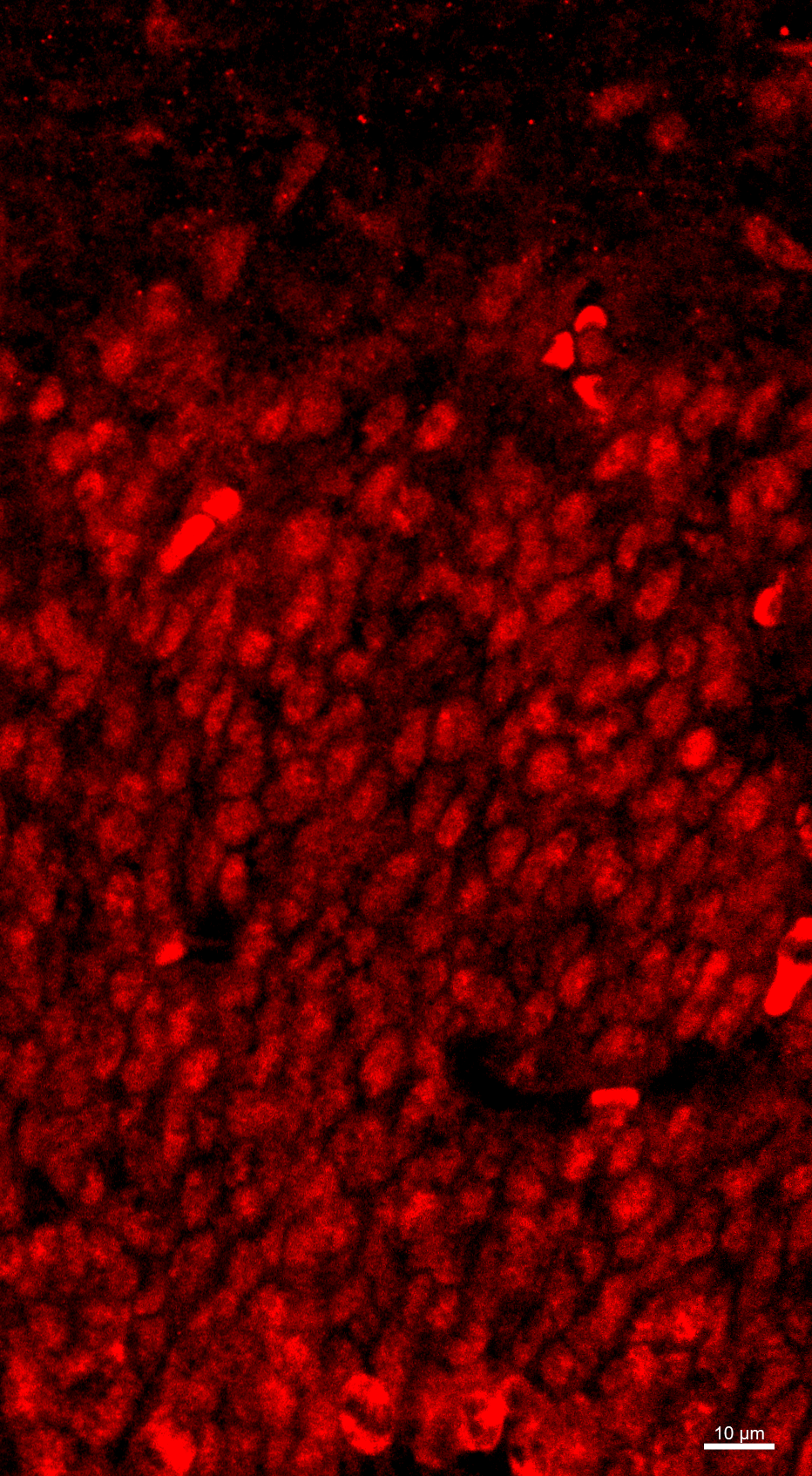

Supplement: Supplementary file 12 — Figure EV3 Source Data [file 44319_2024_82_MOESM12_ESM.zip › Figure EV3/EV3D/Phf6 ko/sox2 inset.tif]

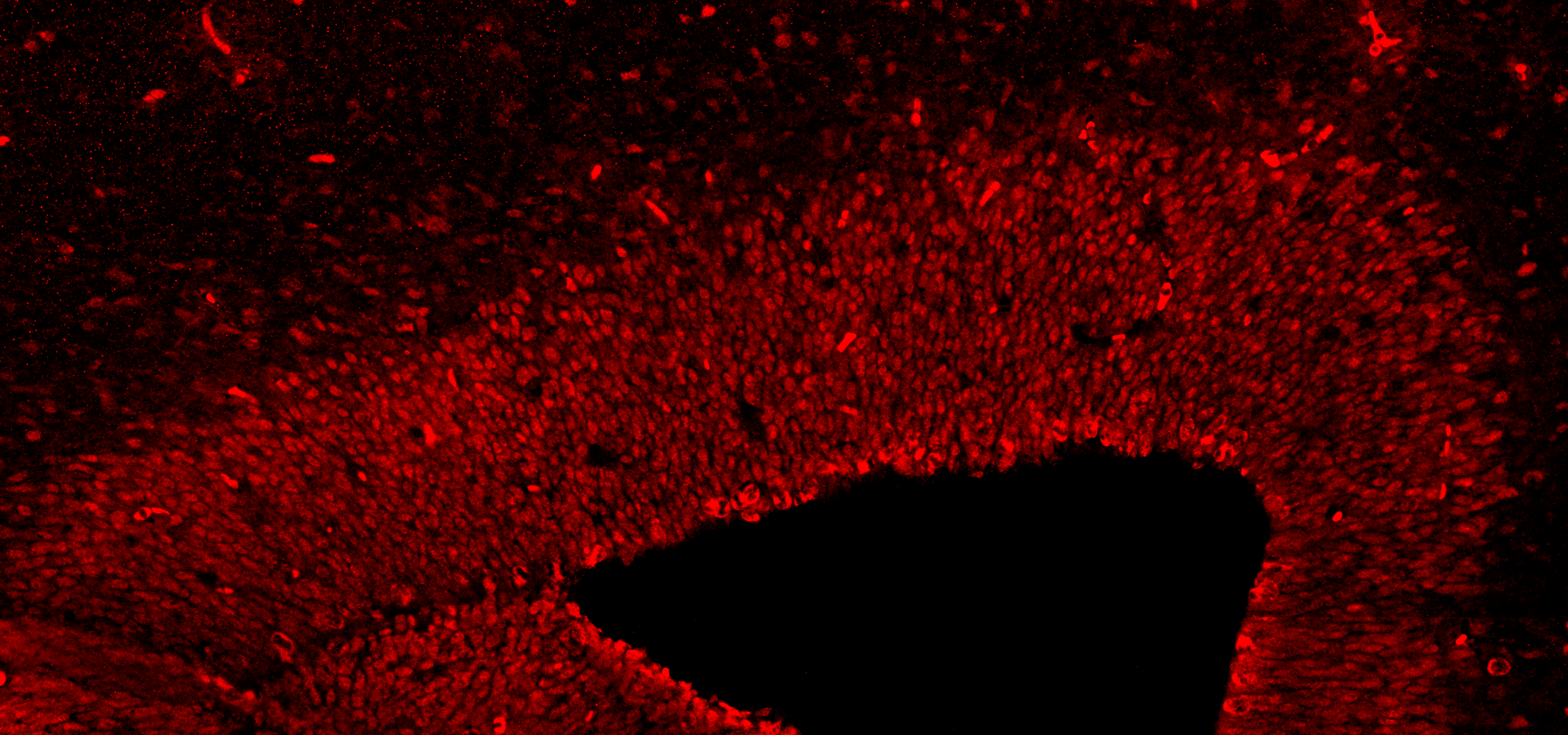

Supplement: Supplementary file 12 — Figure EV3 Source Data [file 44319_2024_82_MOESM12_ESM.zip › Figure EV3/EV3D/Phf6 ko/sox2.tif]

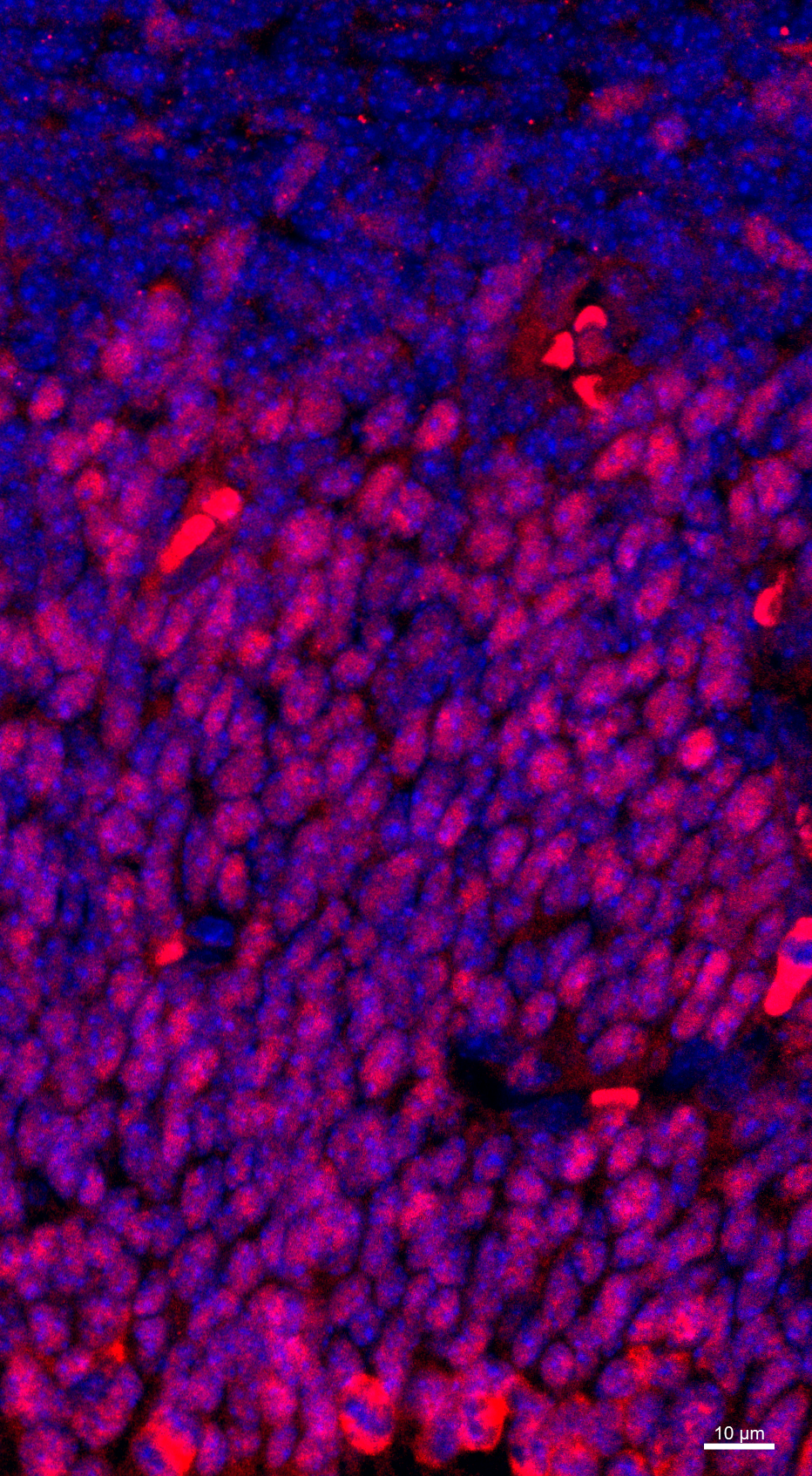

Supplement: Supplementary file 12 — Figure EV3 Source Data [file 44319_2024_82_MOESM12_ESM.zip › Figure EV3/EV3D/Phf6 ko/merged inset.tif]

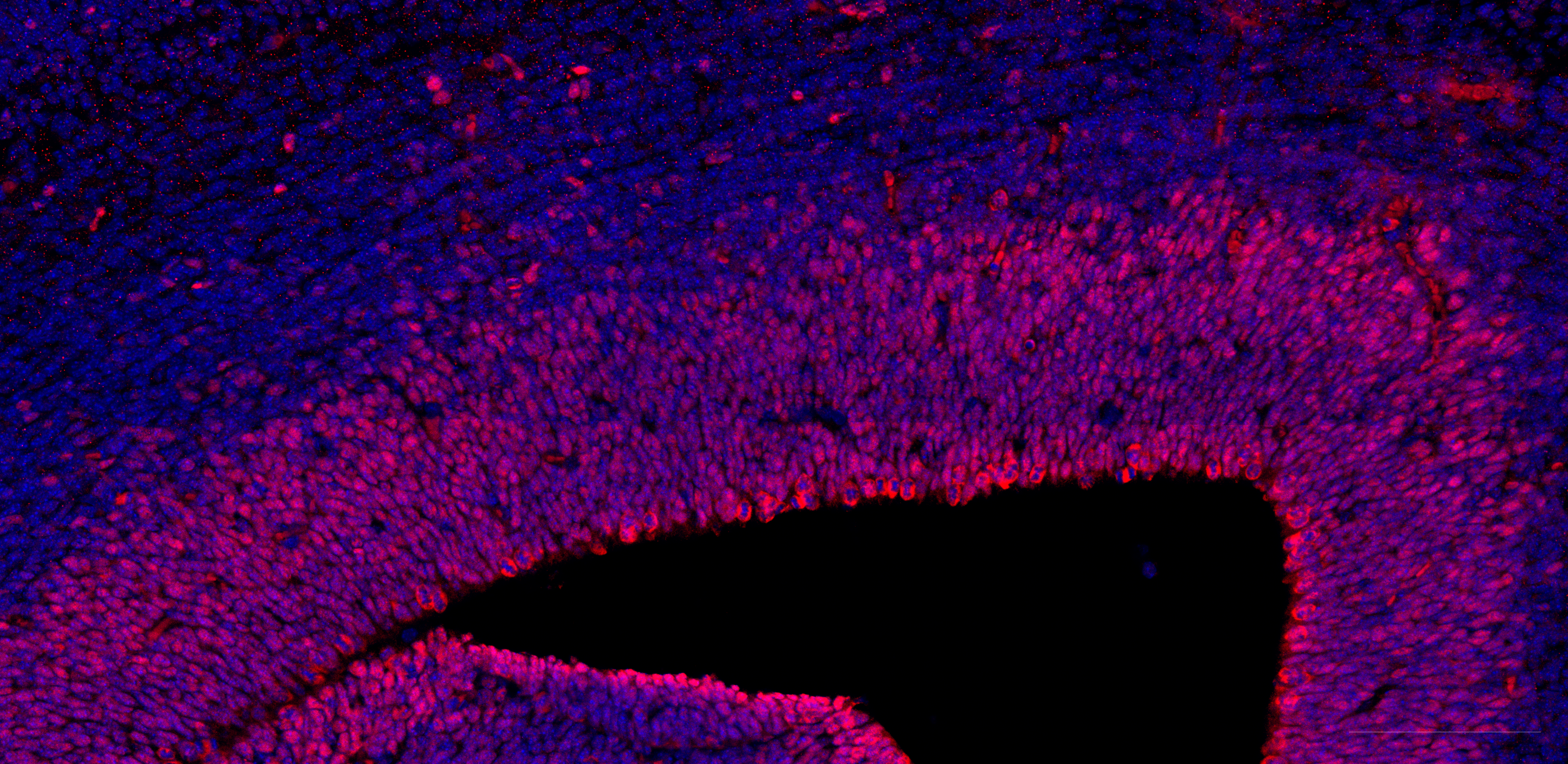

Supplement: Supplementary file 12 — Figure EV3 Source Data [file 44319_2024_82_MOESM12_ESM.zip › Figure EV3/EV3D/Phf6 ctl/merged.tif]

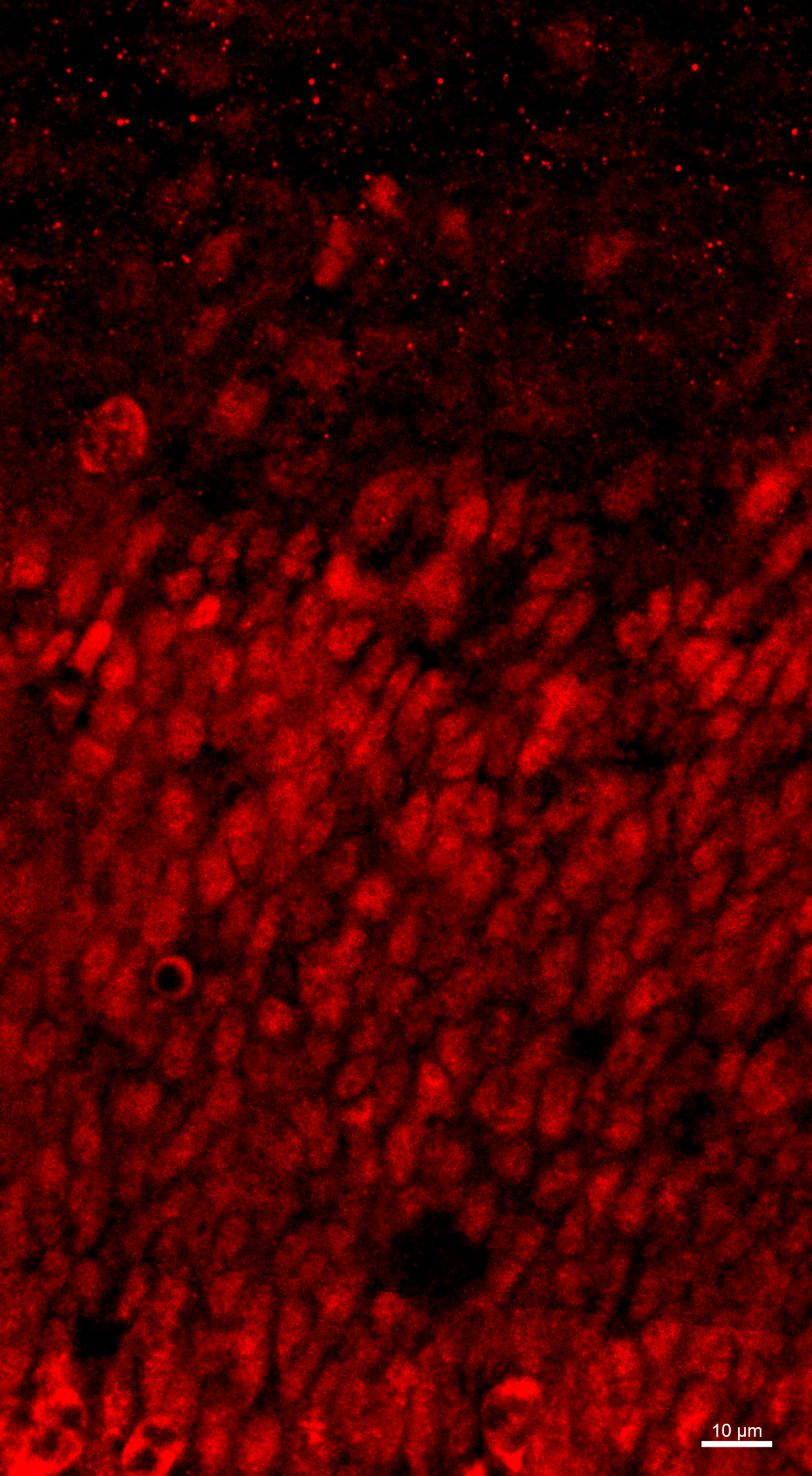

Supplement: Supplementary file 12 — Figure EV3 Source Data [file 44319_2024_82_MOESM12_ESM.zip › Figure EV3/EV3D/Phf6 ctl/sox2 inset.tif]

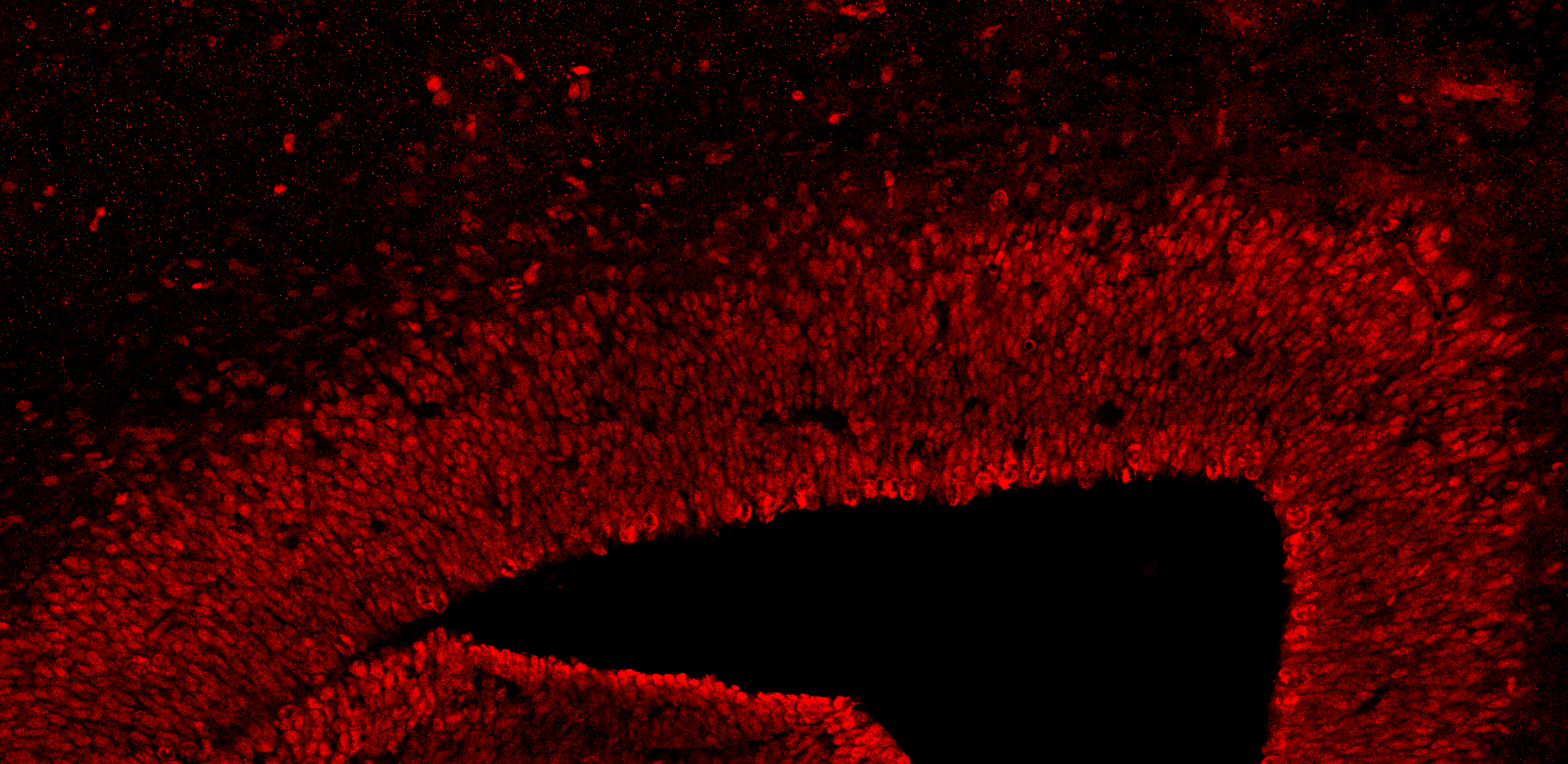

Supplement: Supplementary file 12 — Figure EV3 Source Data [file 44319_2024_82_MOESM12_ESM.zip › Figure EV3/EV3D/Phf6 ctl/sox2.tif]

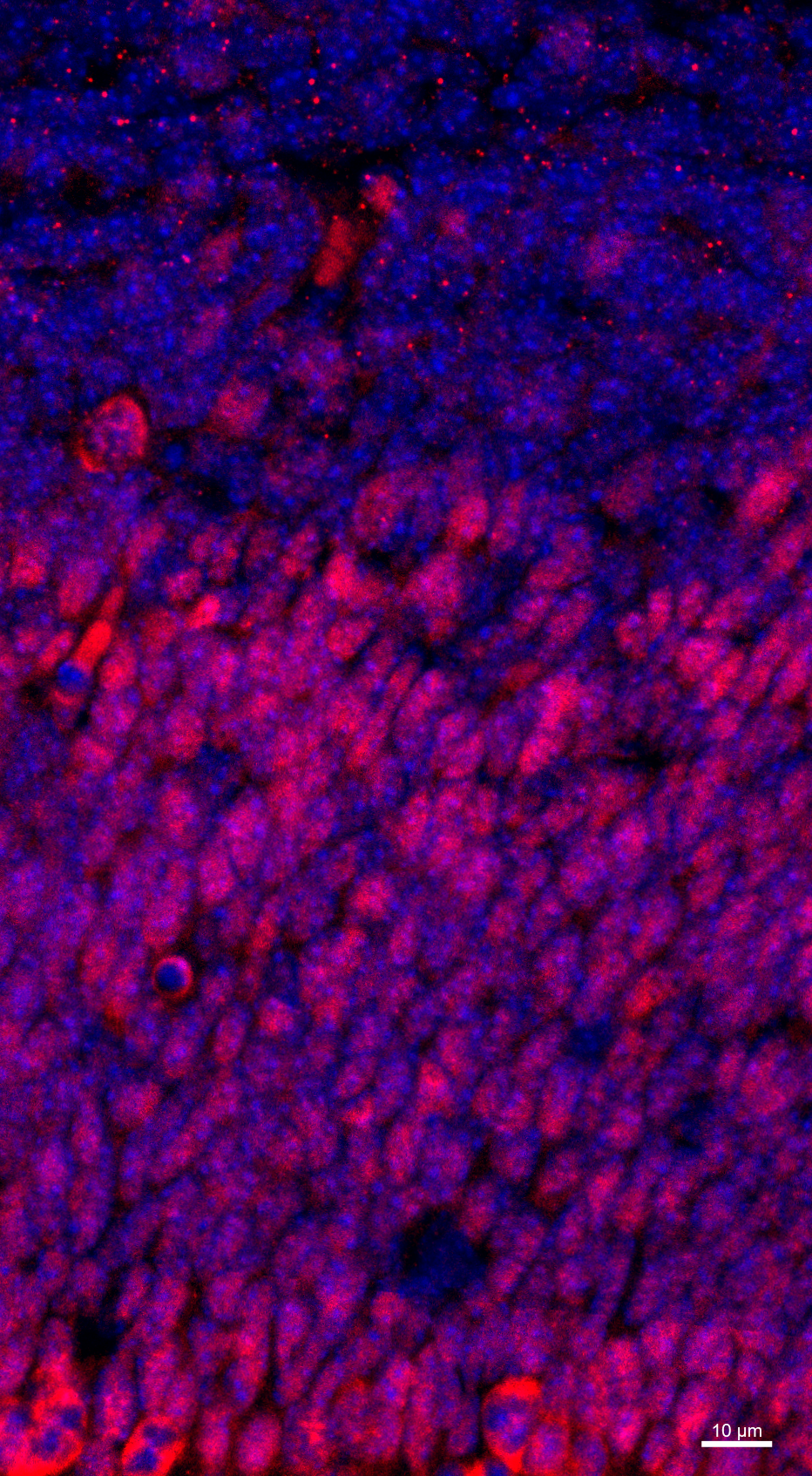

Supplement: Supplementary file 12 — Figure EV3 Source Data [file 44319_2024_82_MOESM12_ESM.zip › Figure EV3/EV3D/Phf6 ctl/merged inset.tif]

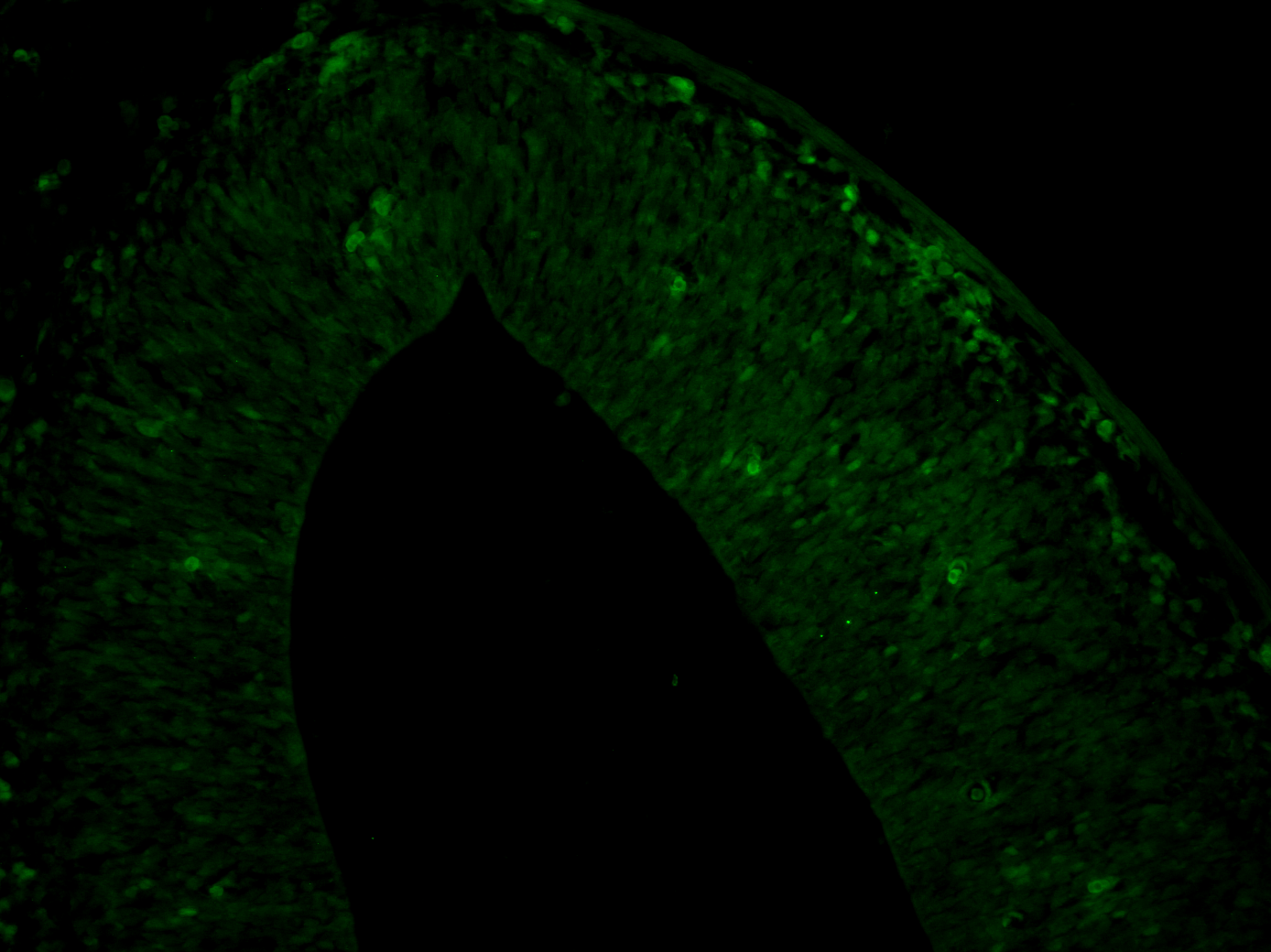

Supplement: Supplementary file 12 — Figure EV3 Source Data [file 44319_2024_82_MOESM12_ESM.zip › Figure EV3/EV3B/Phf6 KO/PHF6.tif]

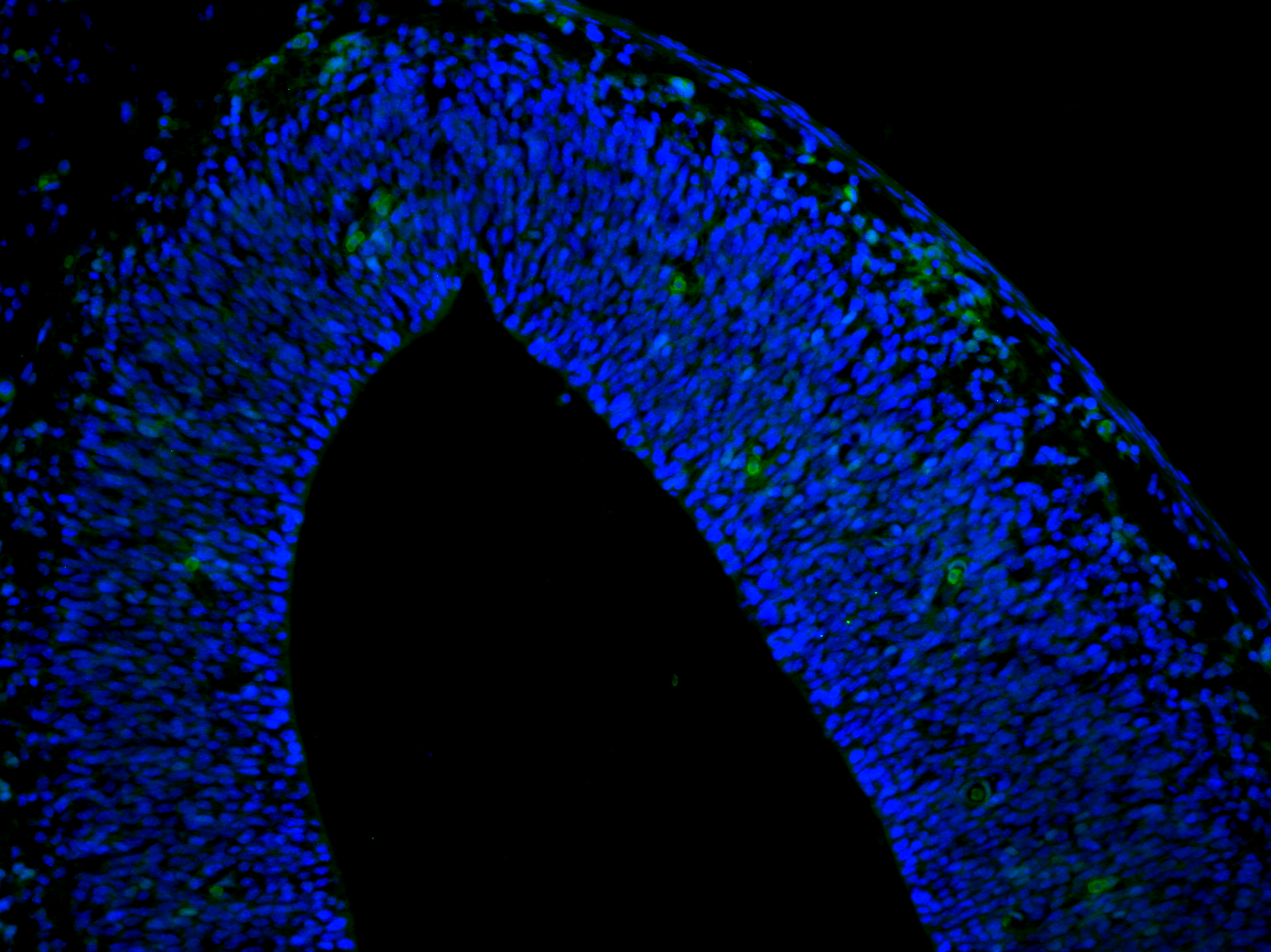

Supplement: Supplementary file 12 — Figure EV3 Source Data [file 44319_2024_82_MOESM12_ESM.zip › Figure EV3/EV3B/Phf6 KO/Merged.tif]

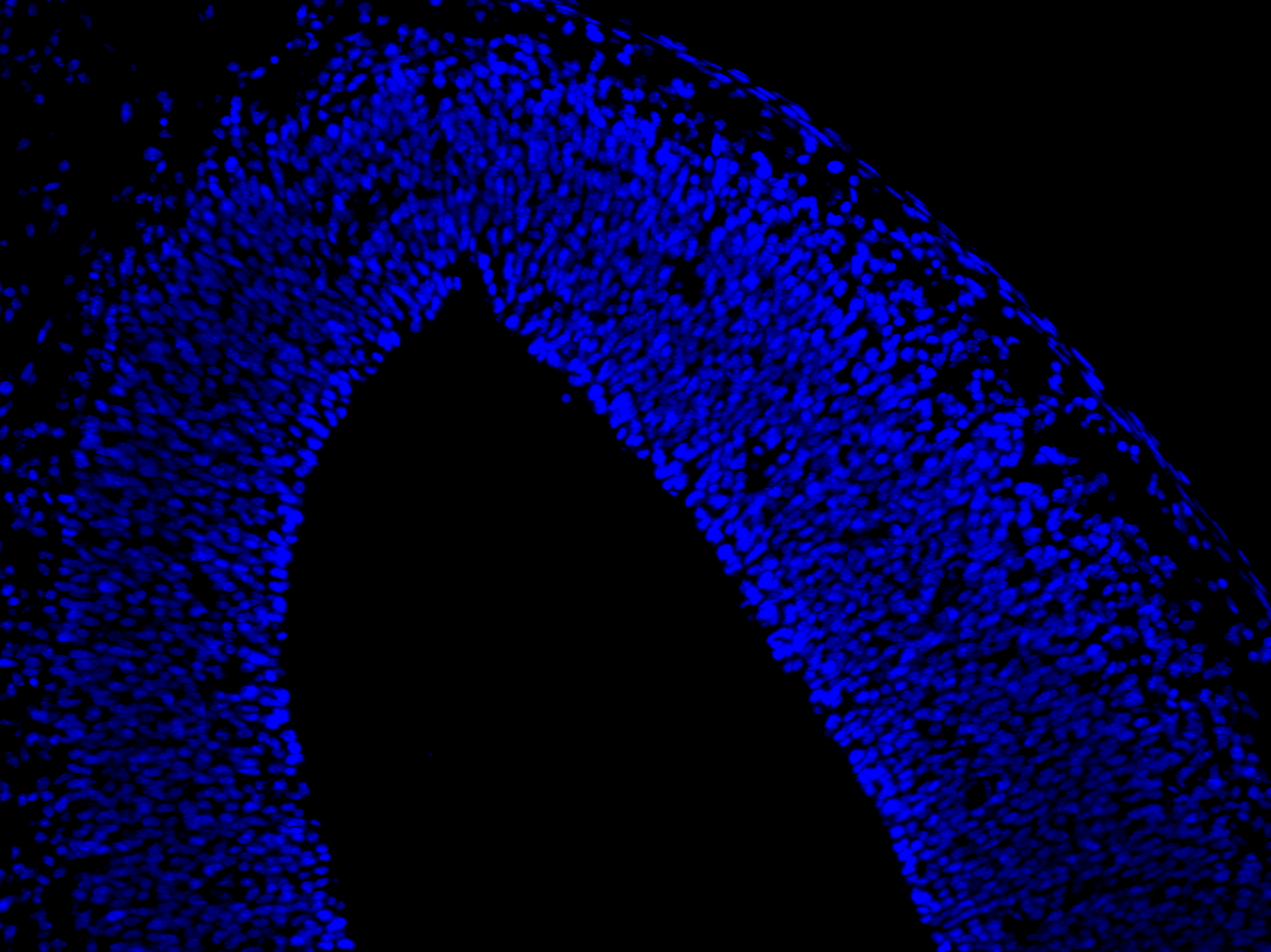

Supplement: Supplementary file 12 — Figure EV3 Source Data [file 44319_2024_82_MOESM12_ESM.zip › Figure EV3/EV3B/Phf6 KO/Hoechst.tif]

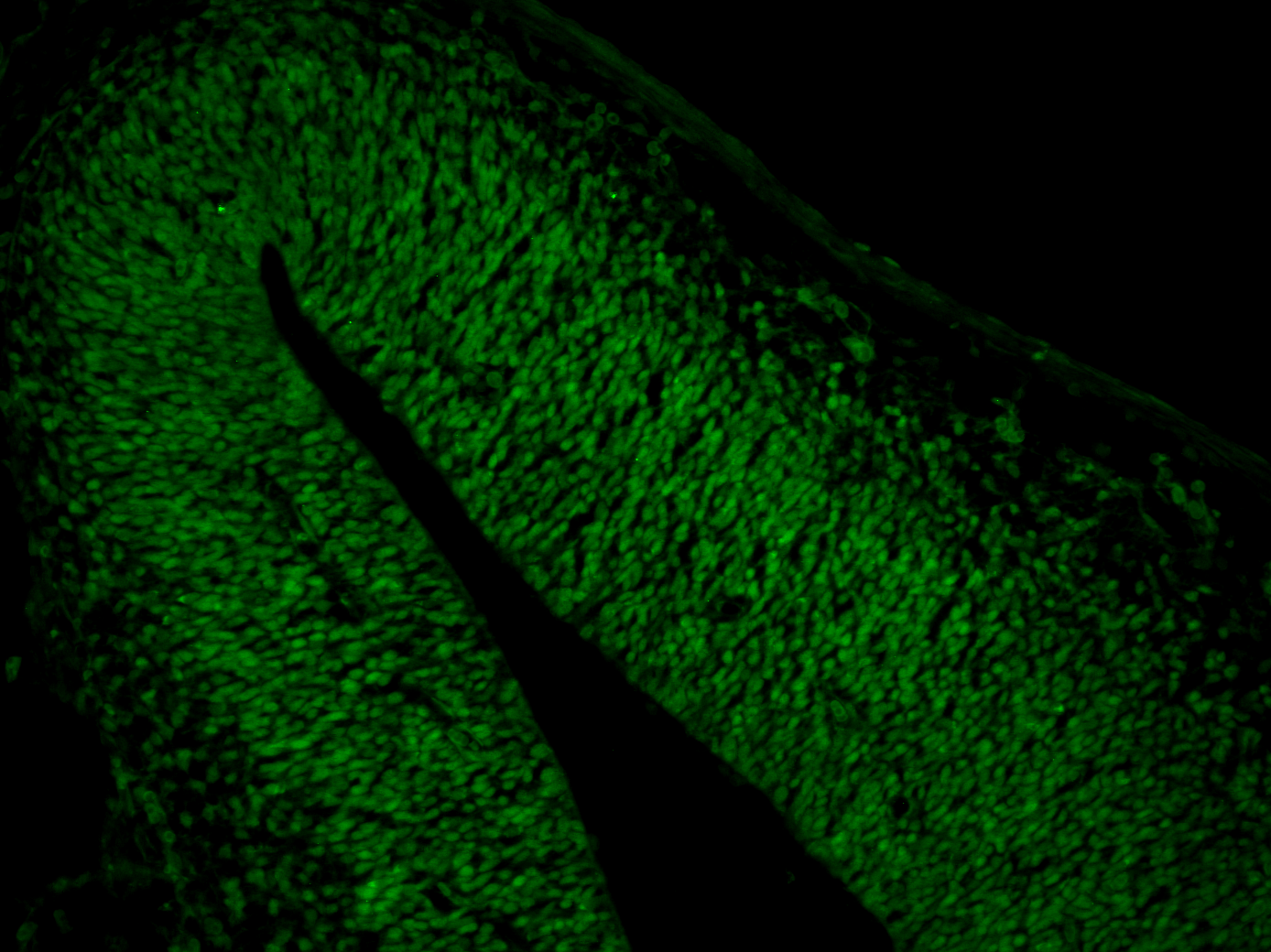

Supplement: Supplementary file 12 — Figure EV3 Source Data [file 44319_2024_82_MOESM12_ESM.zip › Figure EV3/EV3B/Ctl/PHF6.tif]

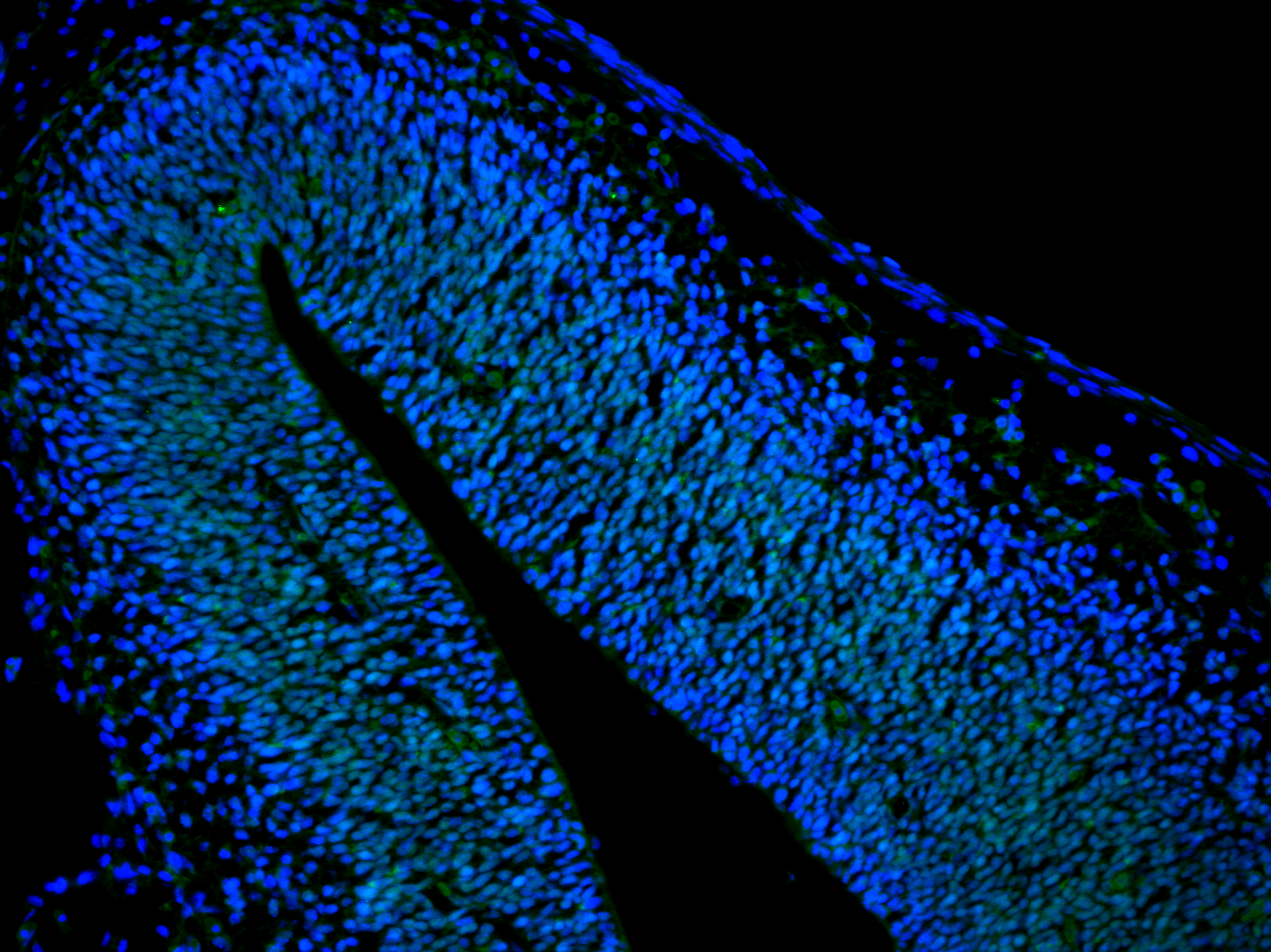

Supplement: Supplementary file 12 — Figure EV3 Source Data [file 44319_2024_82_MOESM12_ESM.zip › Figure EV3/EV3B/Ctl/Merged.tif]

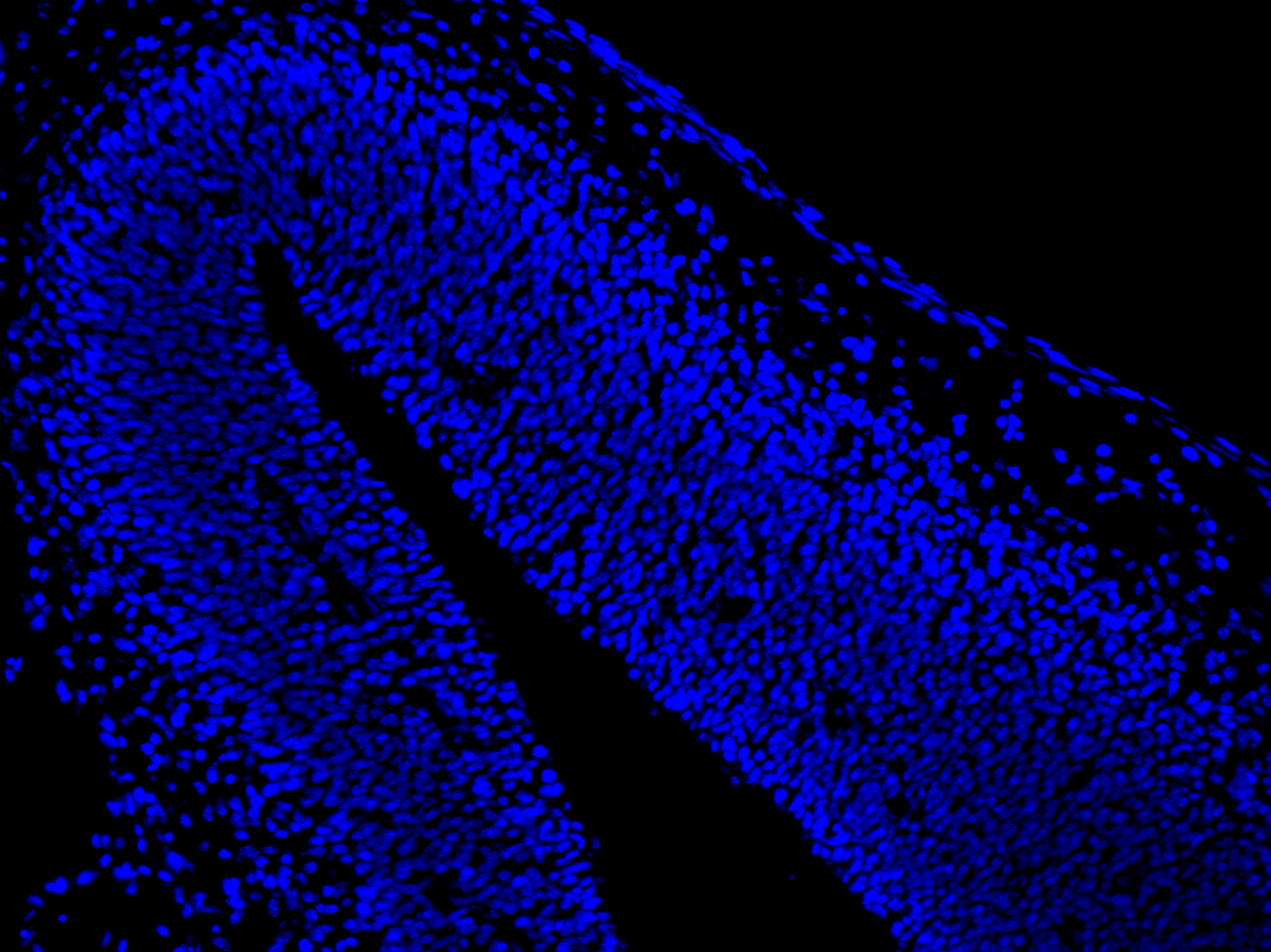

Supplement: Supplementary file 12 — Figure EV3 Source Data [file 44319_2024_82_MOESM12_ESM.zip › Figure EV3/EV3B/Ctl/Hoechst.tif]

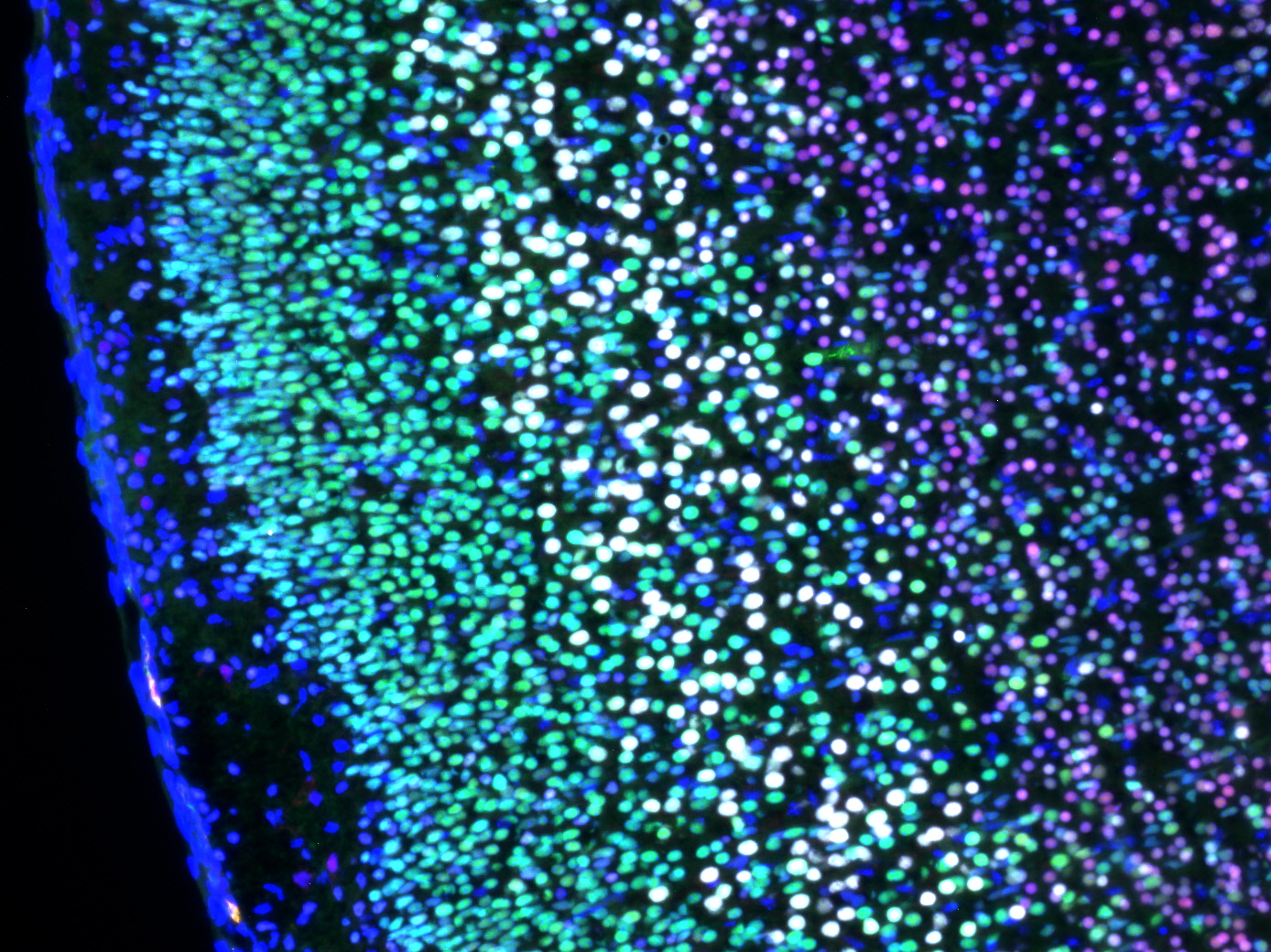

Supplement: Supplementary file 12 — Figure EV3 Source Data [file 44319_2024_82_MOESM12_ESM.zip › Figure EV3/EV3E/Phf6 KO/Merged top.JPG]

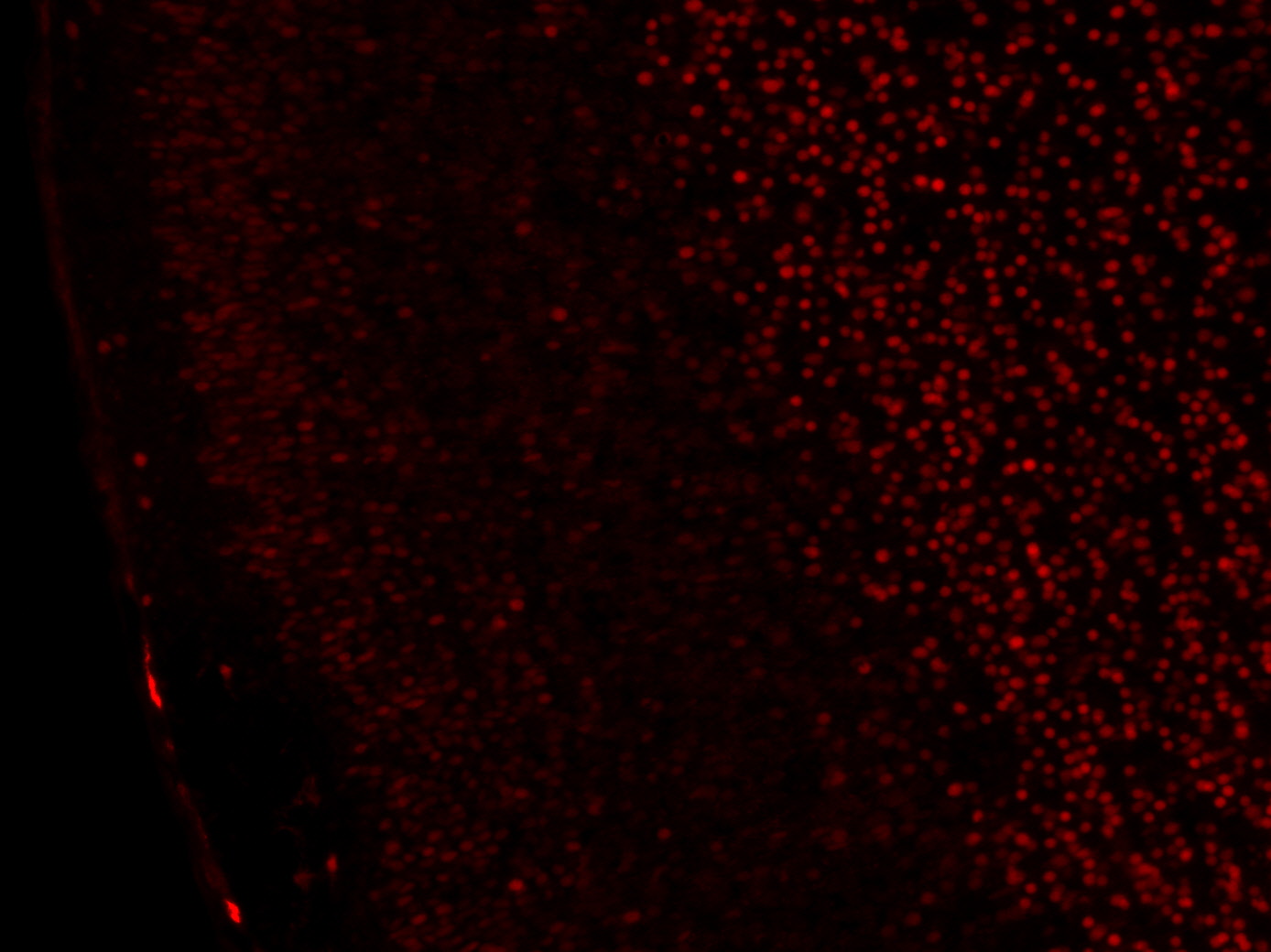

Supplement: Supplementary file 12 — Figure EV3 Source Data [file 44319_2024_82_MOESM12_ESM.zip › Figure EV3/EV3E/Phf6 KO/Tbr1 top.JPG]

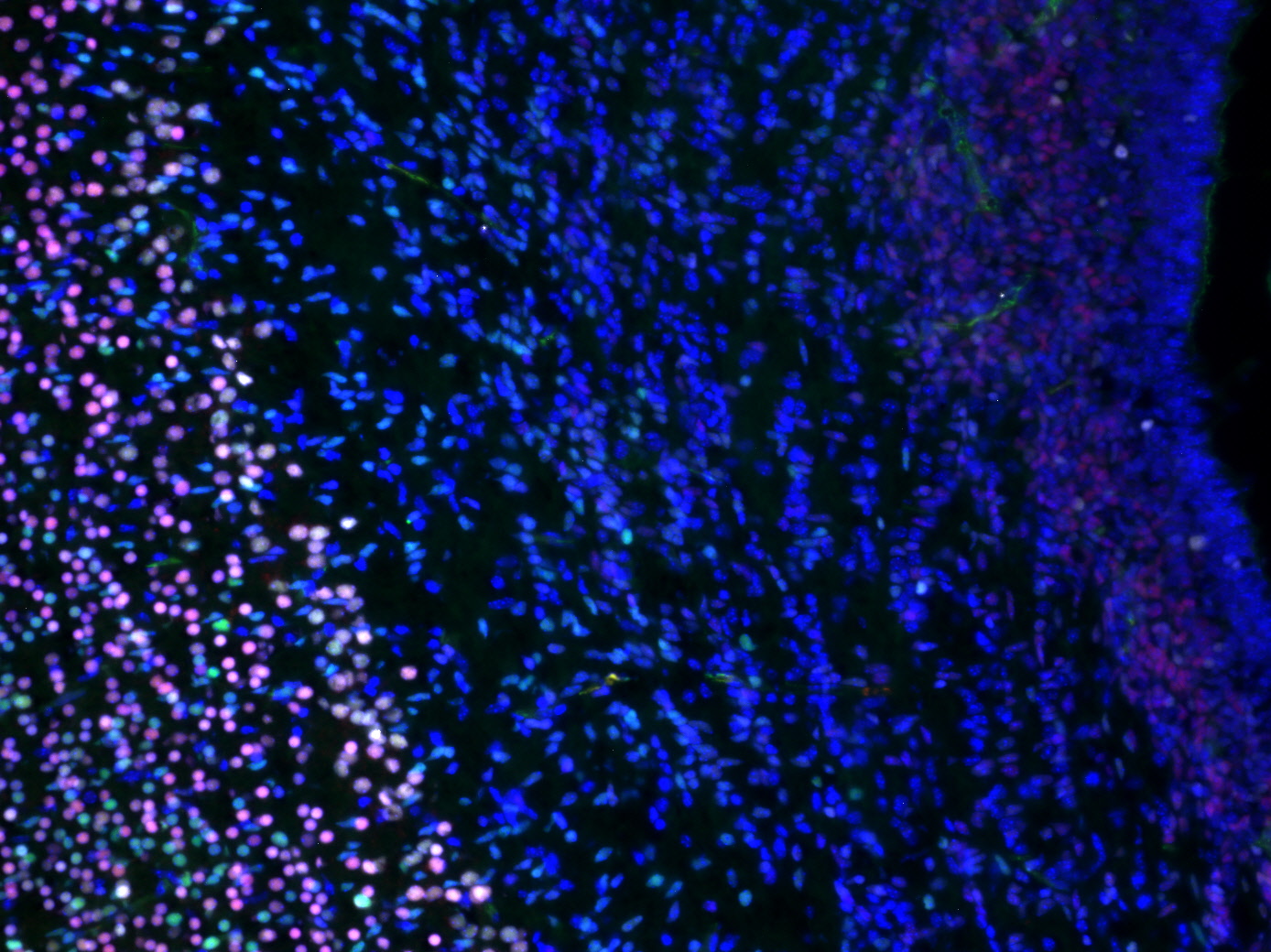

Supplement: Supplementary file 12 — Figure EV3 Source Data [file 44319_2024_82_MOESM12_ESM.zip › Figure EV3/EV3E/Phf6 KO/Merged bottom.JPG]

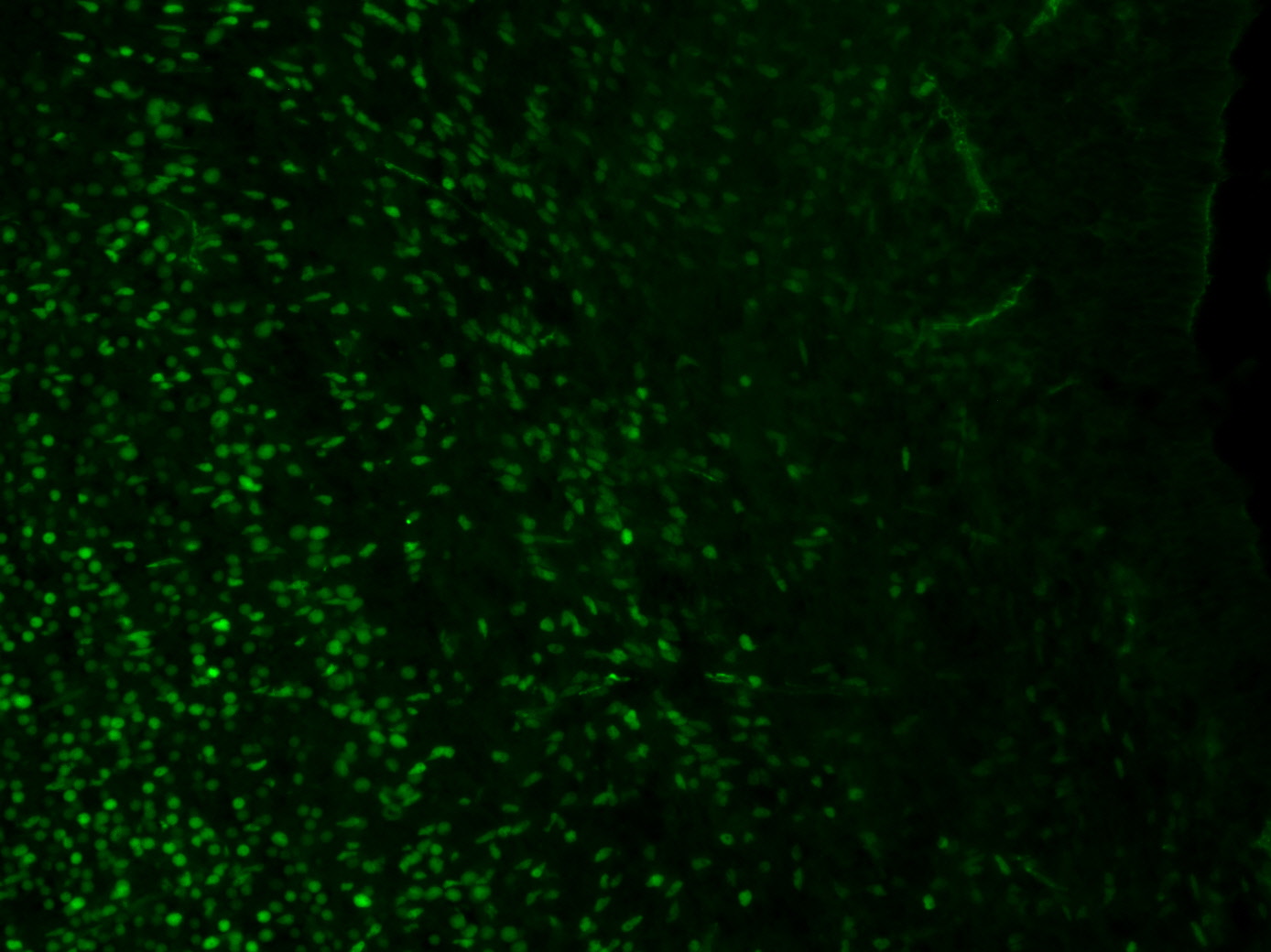

Supplement: Supplementary file 12 — Figure EV3 Source Data [file 44319_2024_82_MOESM12_ESM.zip › Figure EV3/EV3E/Phf6 KO/Satb2 bottom.JPG]

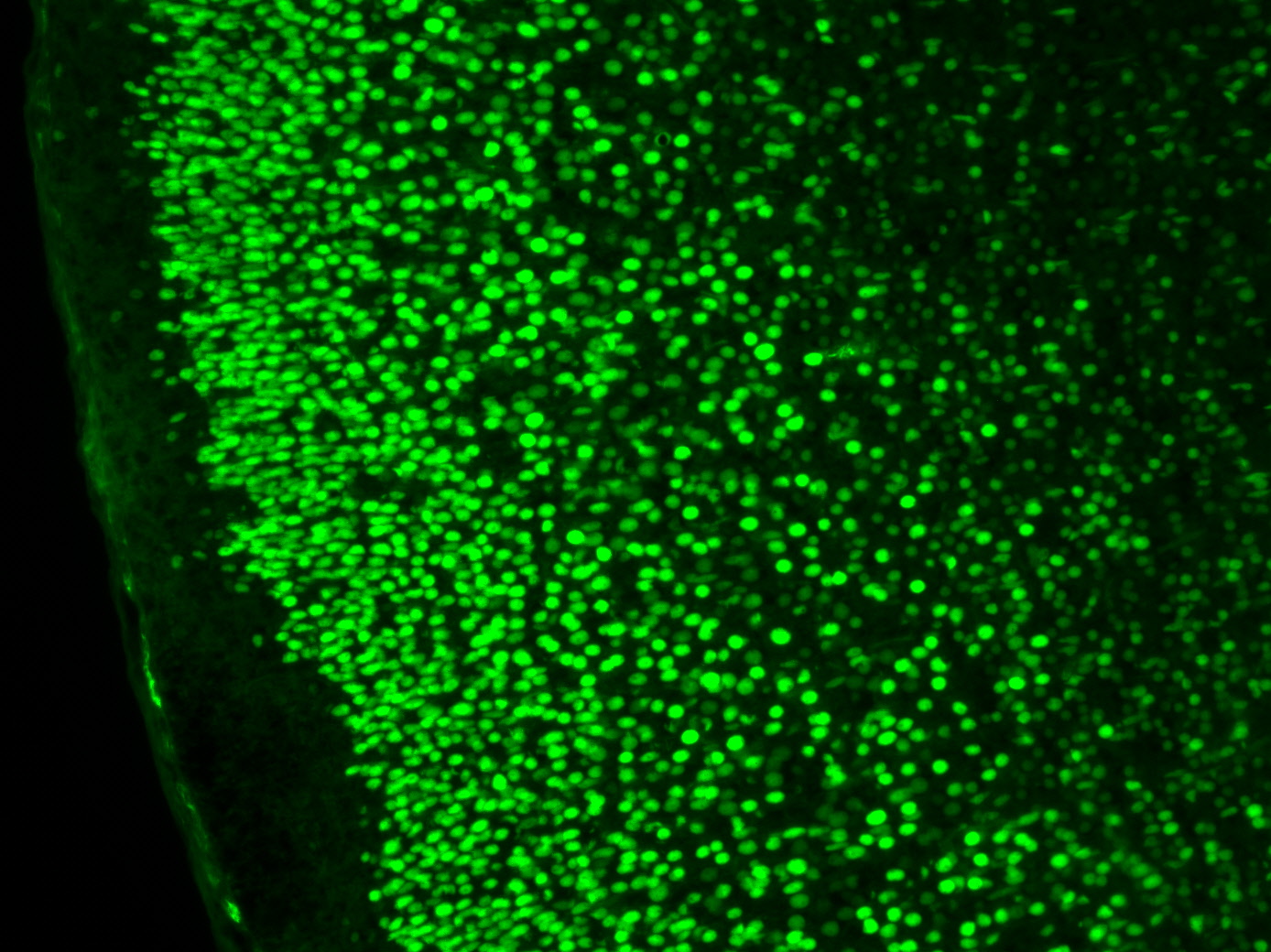

Supplement: Supplementary file 12 — Figure EV3 Source Data [file 44319_2024_82_MOESM12_ESM.zip › Figure EV3/EV3E/Phf6 KO/Satb2 top.JPG]

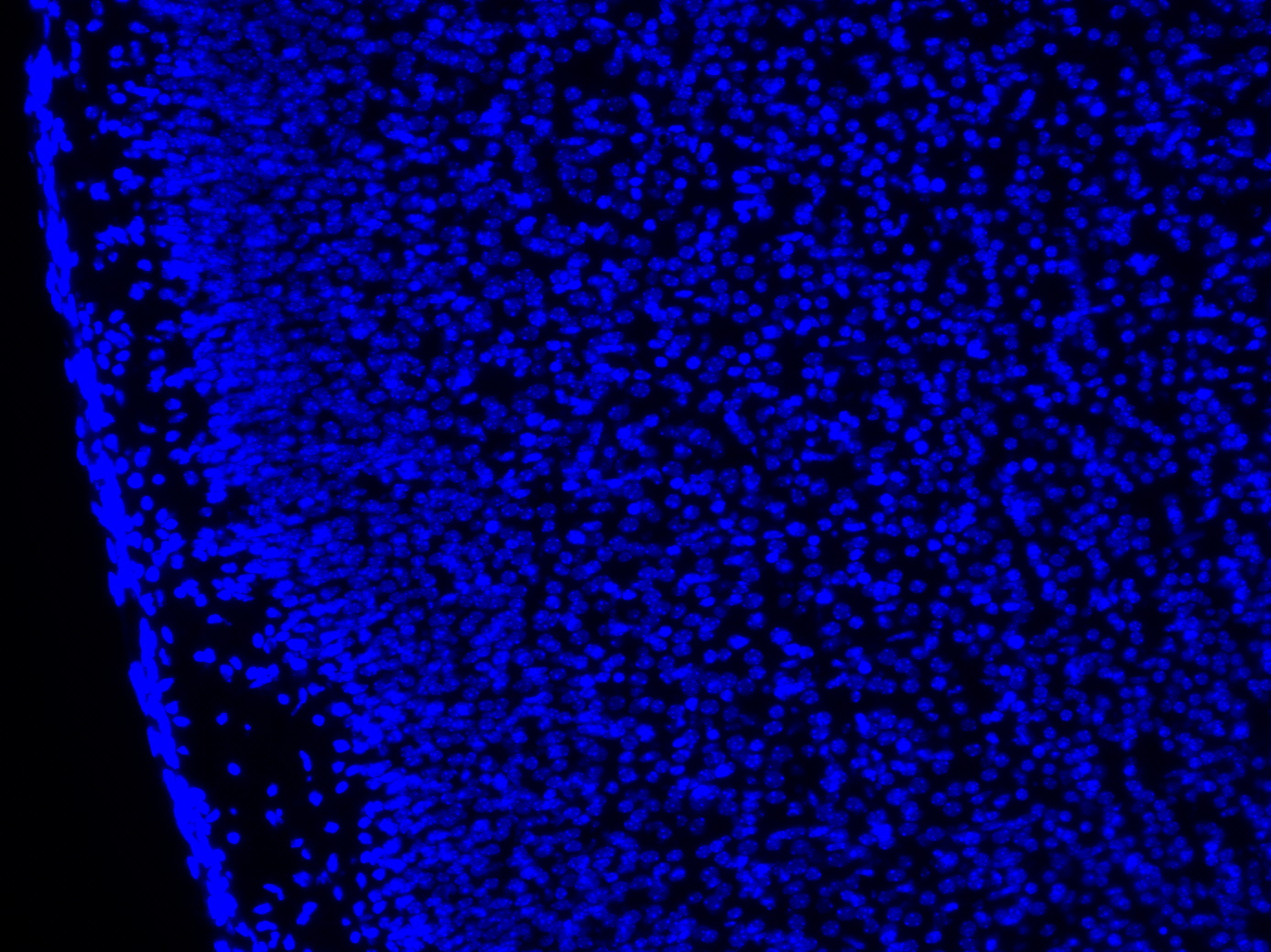

Supplement: Supplementary file 12 — Figure EV3 Source Data [file 44319_2024_82_MOESM12_ESM.zip › Figure EV3/EV3E/Phf6 KO/Hoechst top.JPG]

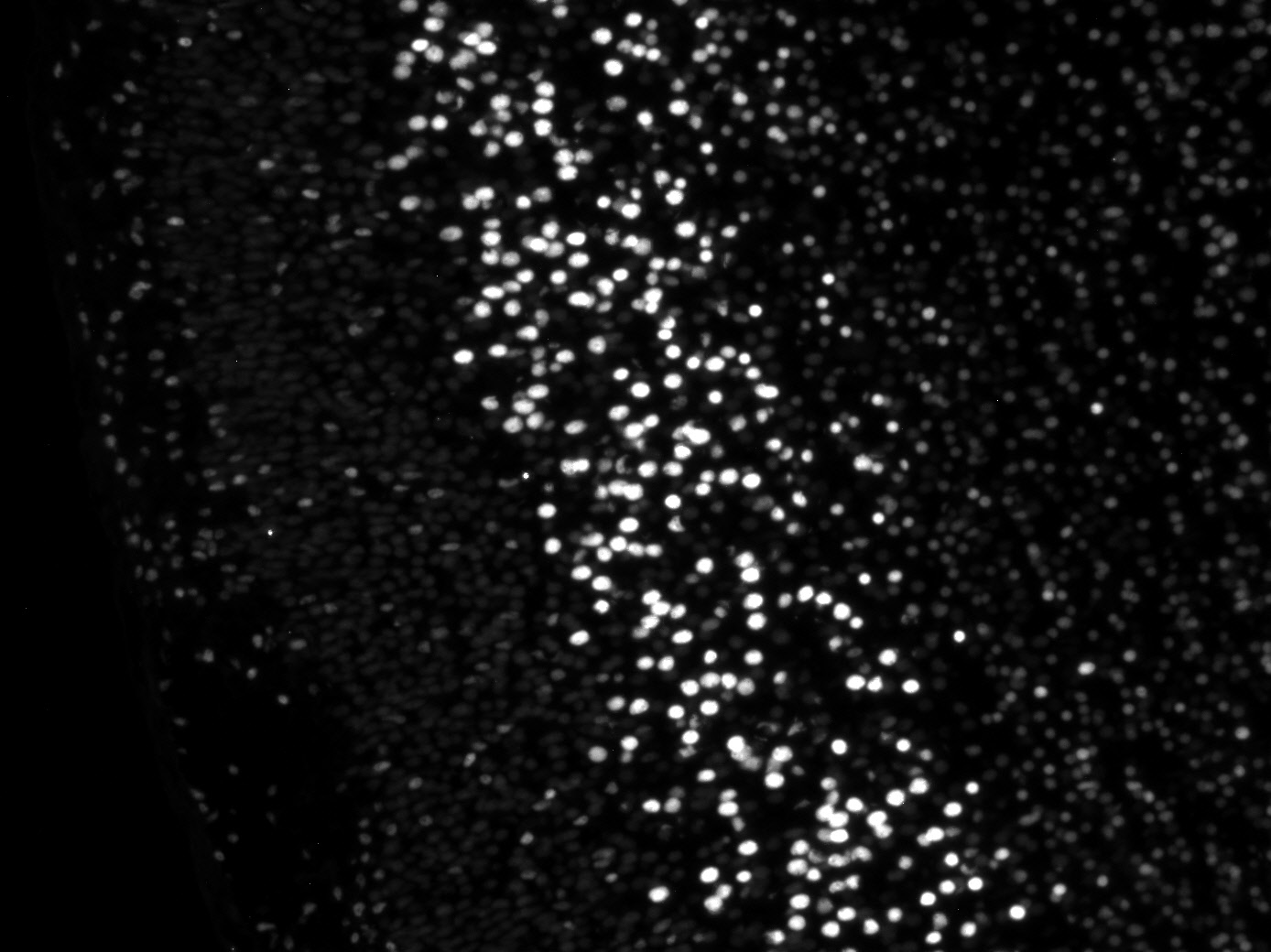

Supplement: Supplementary file 12 — Figure EV3 Source Data [file 44319_2024_82_MOESM12_ESM.zip › Figure EV3/EV3E/Phf6 KO/Ctip2 top.JPG]

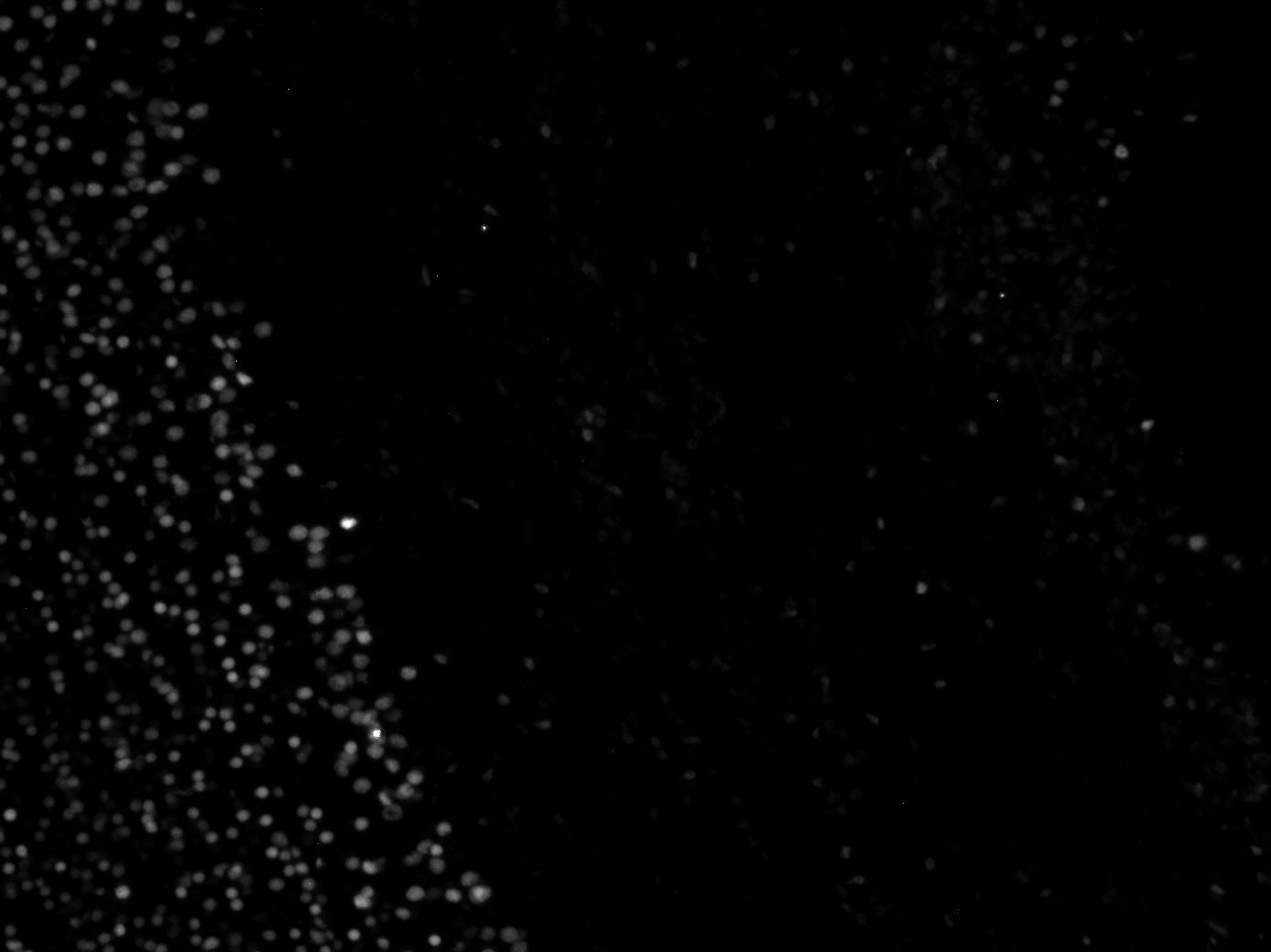

Supplement: Supplementary file 12 — Figure EV3 Source Data [file 44319_2024_82_MOESM12_ESM.zip › Figure EV3/EV3E/Phf6 KO/Ctip2 bottom.JPG]

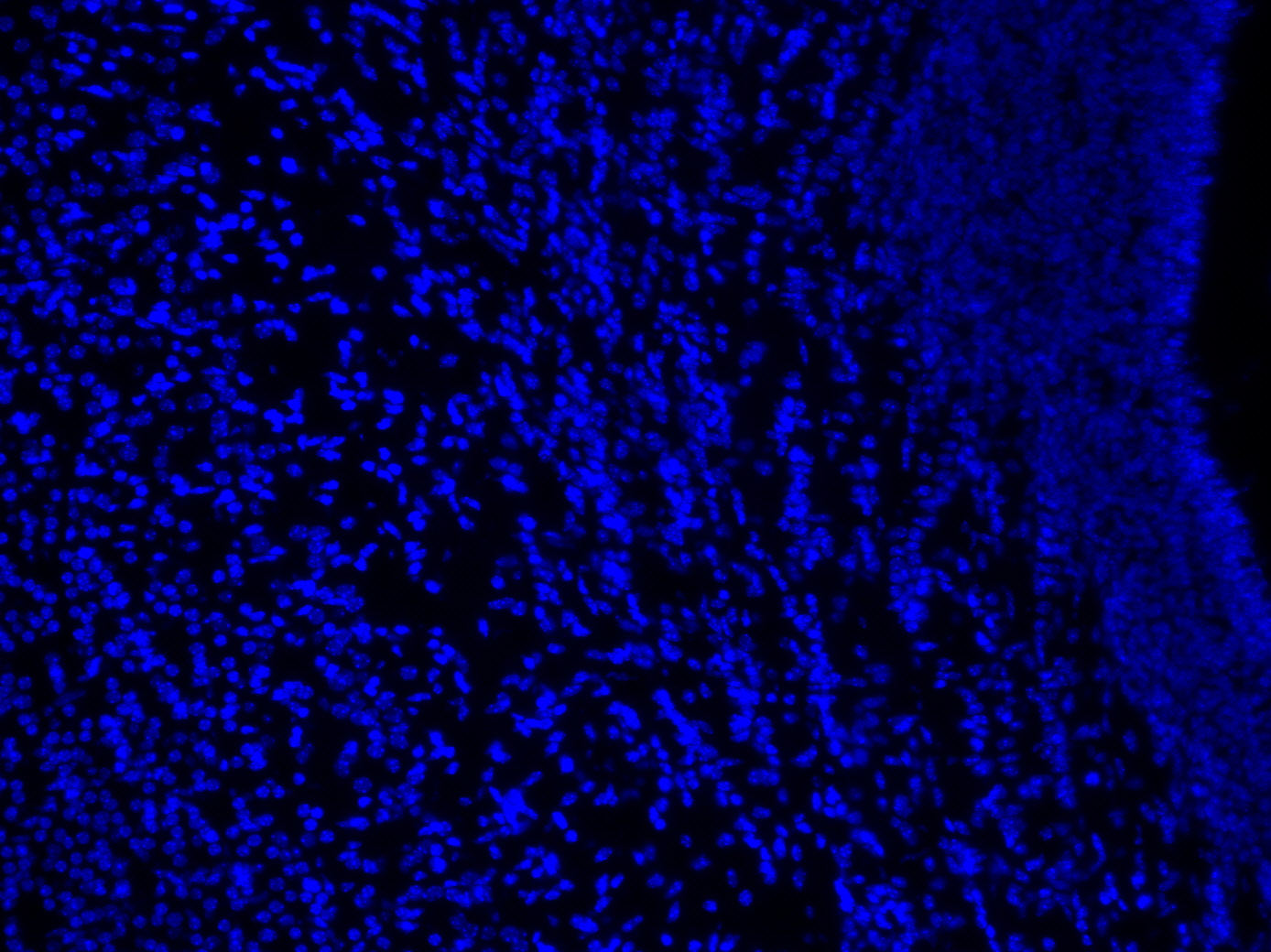

Supplement: Supplementary file 12 — Figure EV3 Source Data [file 44319_2024_82_MOESM12_ESM.zip › Figure EV3/EV3E/Phf6 KO/Hoechst bottom.JPG]
