# Supplementary figures and images for: PHF6-mediated transcriptional control of NSC via Ephrin receptors is impaired in the intellectual disability syndrome BFLS (part 2 of 2)
Source: EMBO Rep. 2024 Mar 1;25(3):20. doi: 10.1038/s44319-024-00082-0 (PMC10933485; doi:10.1038/s44319-024-00082-0)

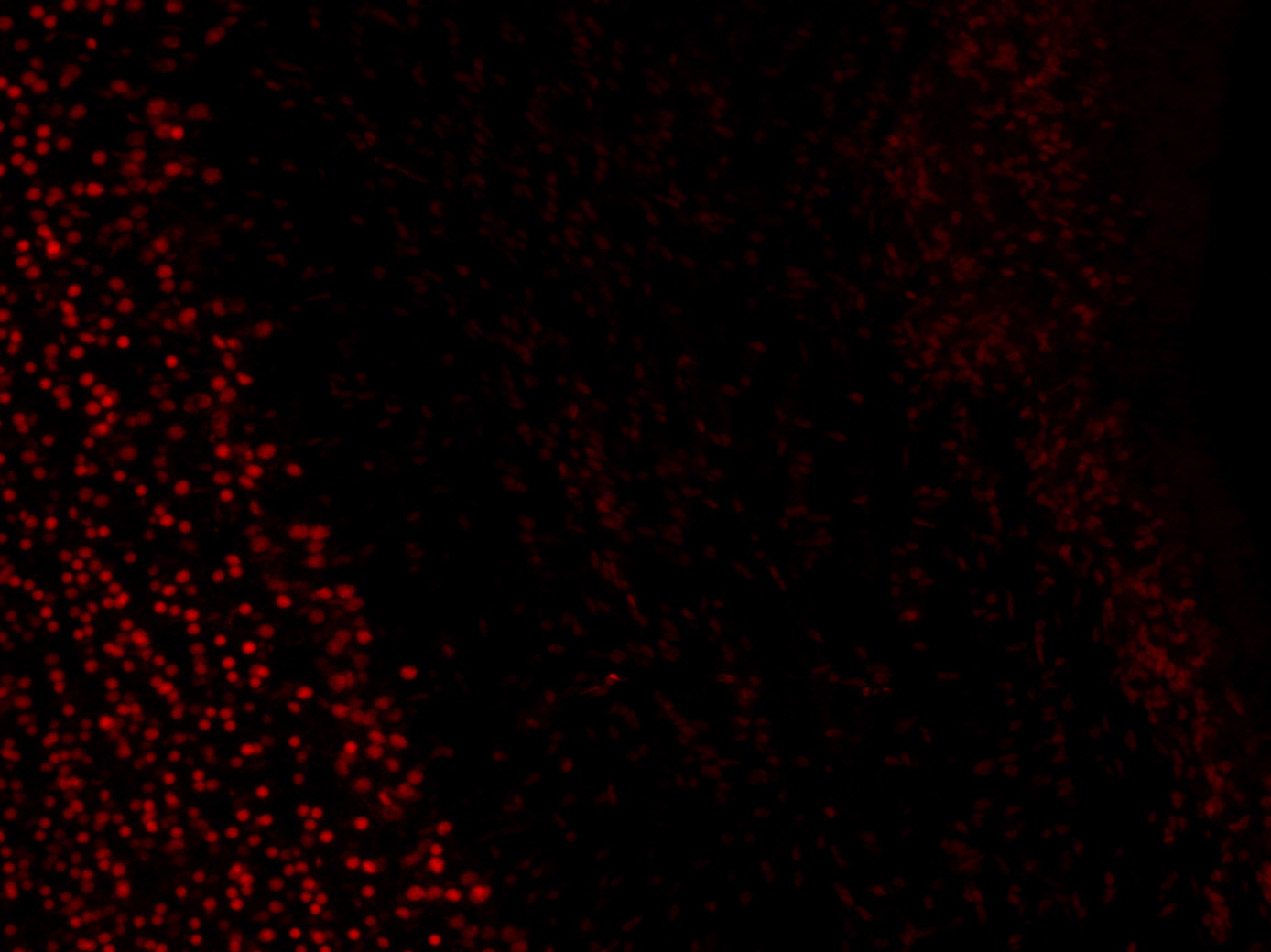

Supplement: Supplementary file 12 — Figure EV3 Source Data [file 44319_2024_82_MOESM12_ESM.zip › Figure EV3/EV3E/Phf6 KO/Tbr1 bottom.JPG]

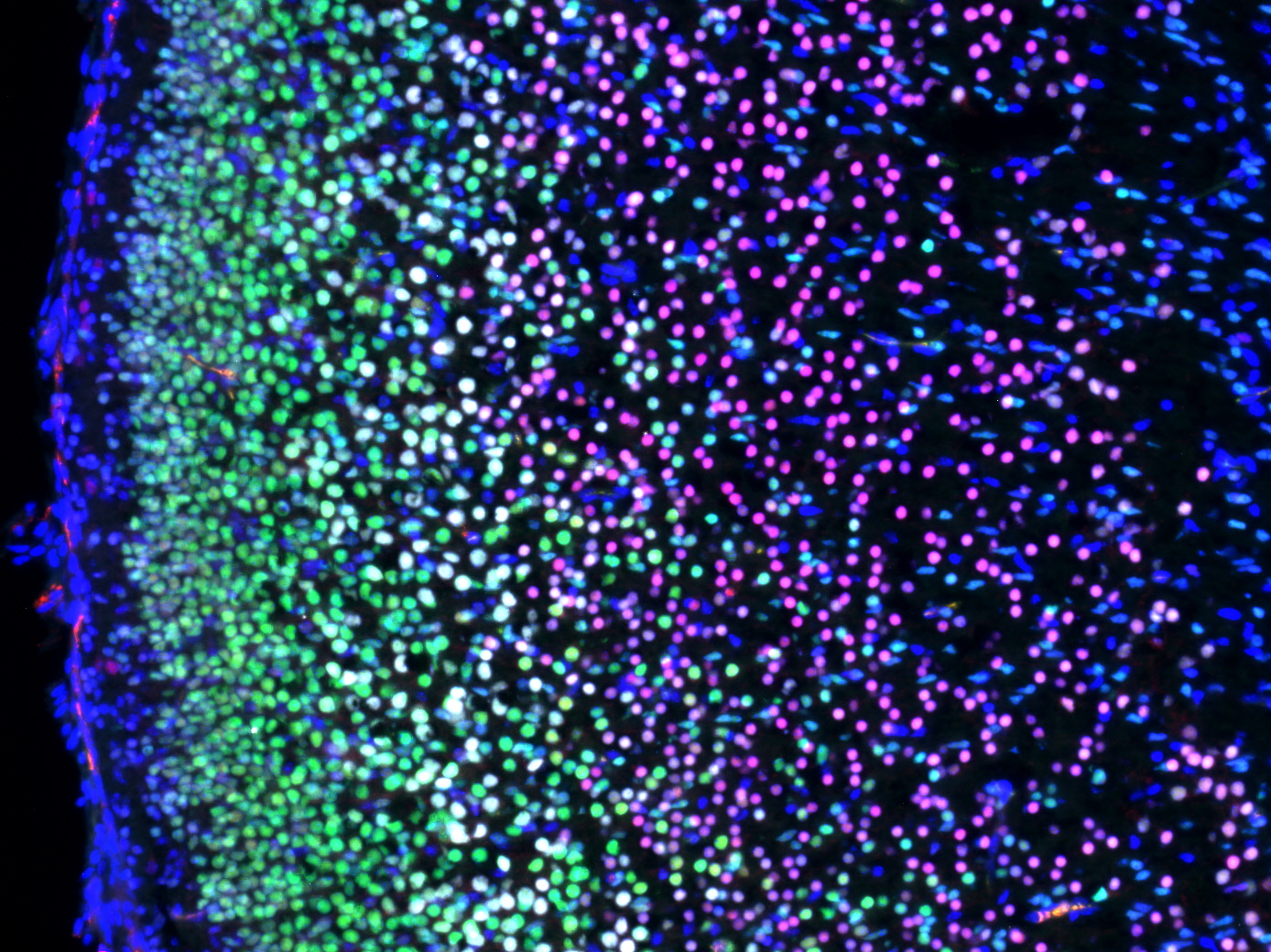

Supplement: Supplementary file 12 — Figure EV3 Source Data [file 44319_2024_82_MOESM12_ESM.zip › Figure EV3/EV3E/Ctl/Merged top.JPG]

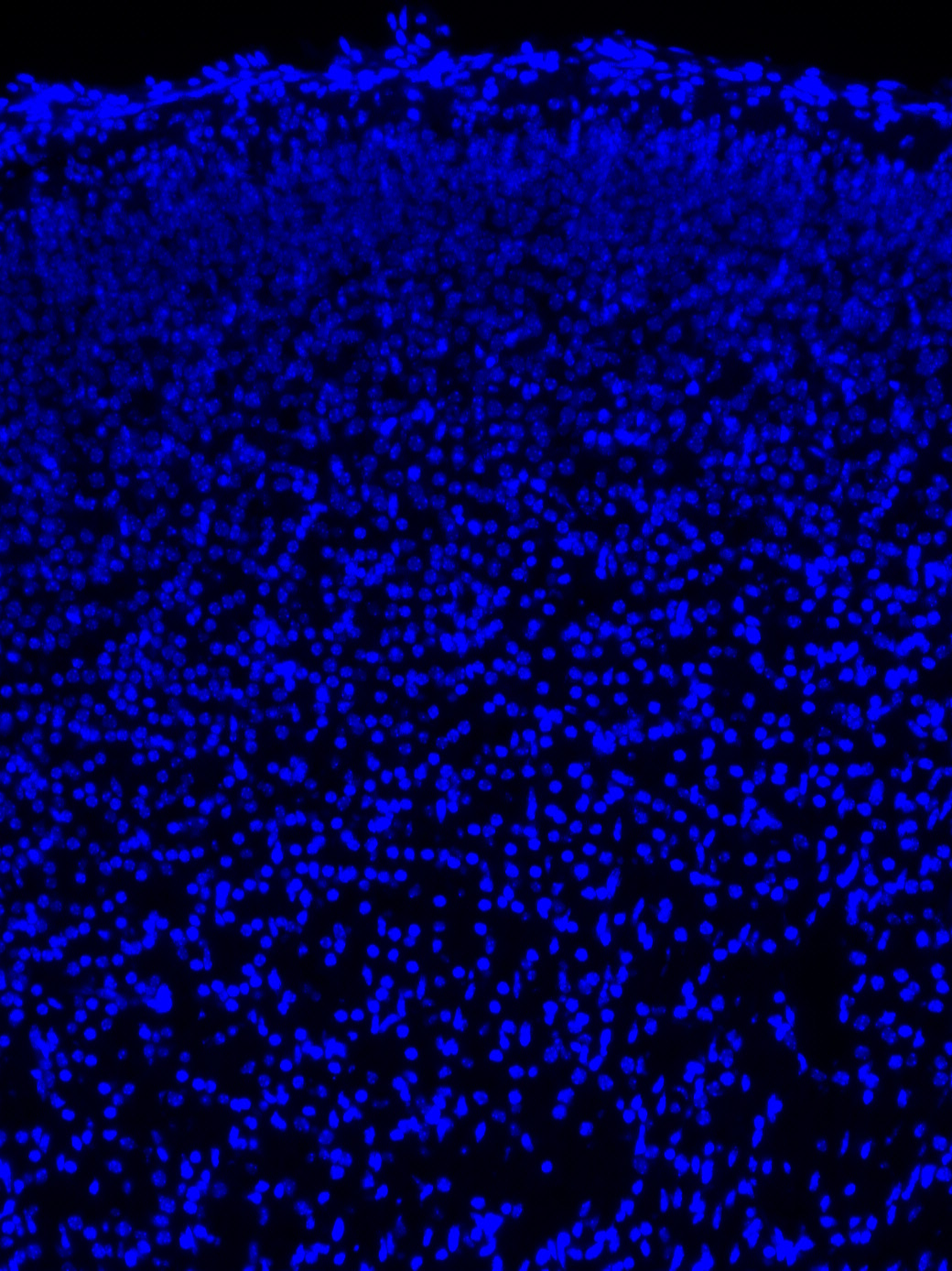

Supplement: Supplementary file 12 — Figure EV3 Source Data [file 44319_2024_82_MOESM12_ESM.zip › Figure EV3/EV3E/Ctl/Hoechst top.tif]

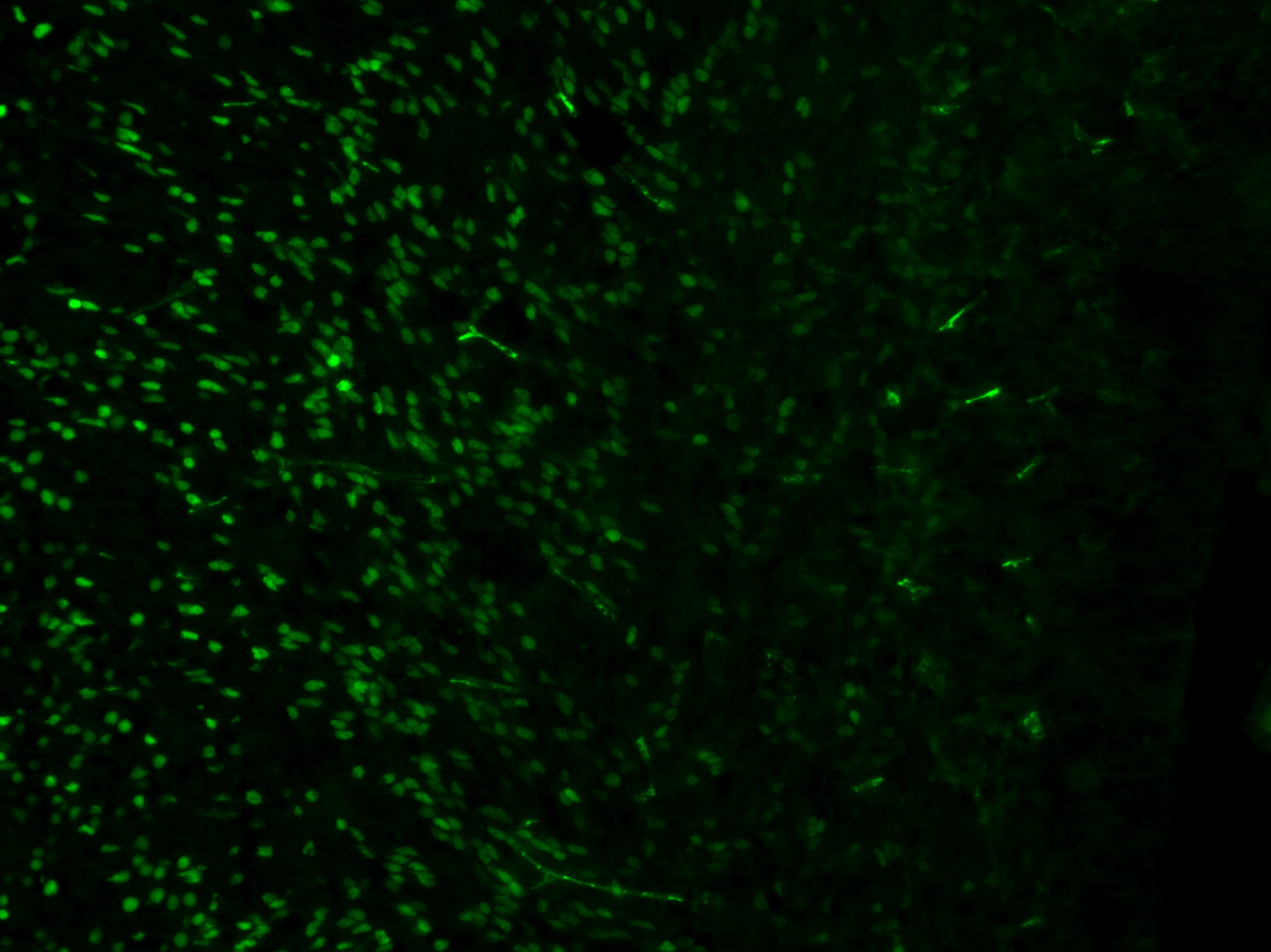

Supplement: Supplementary file 12 — Figure EV3 Source Data [file 44319_2024_82_MOESM12_ESM.zip › Figure EV3/EV3E/Ctl/Satb2 bottom.JPG]

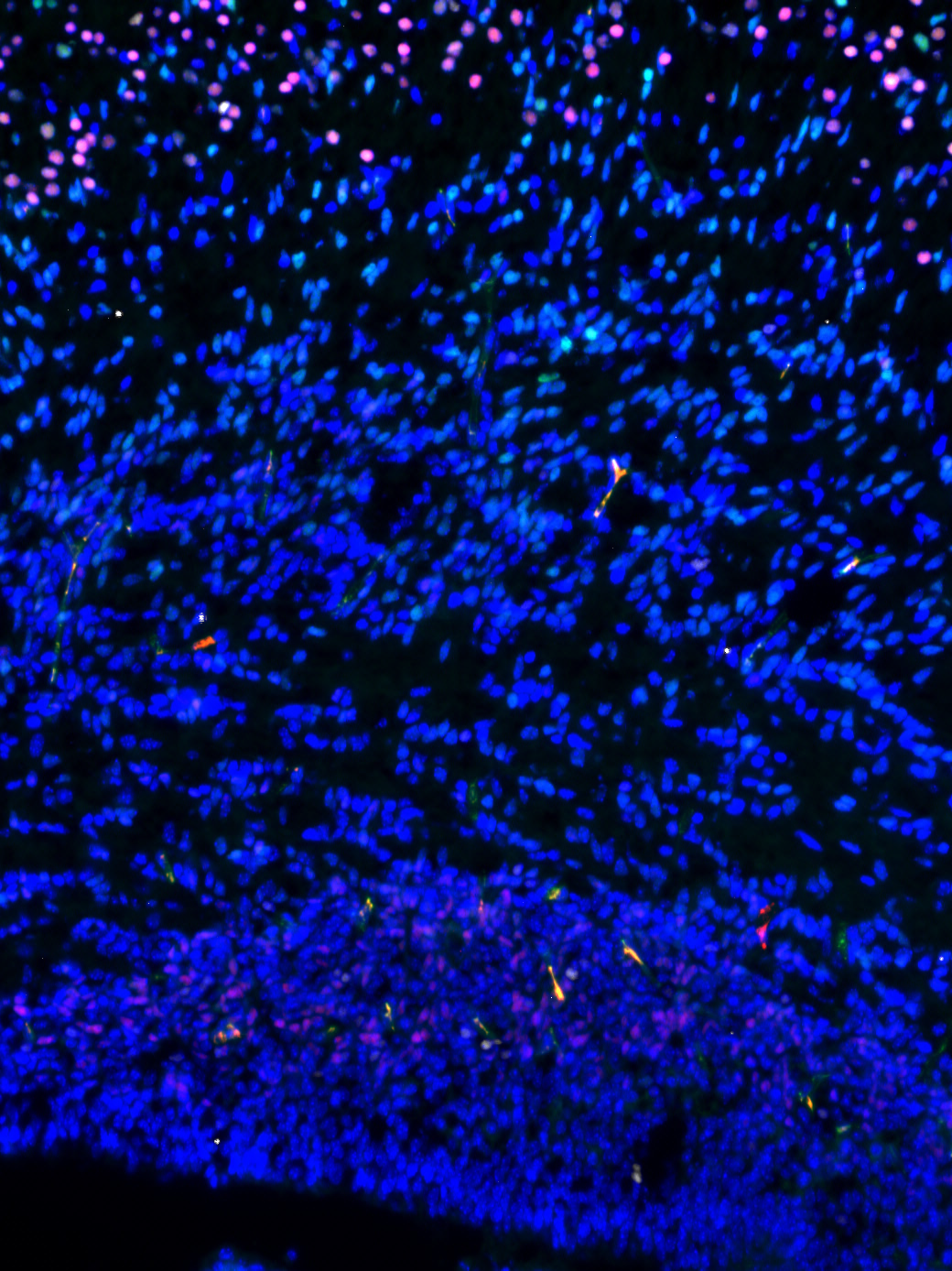

Supplement: Supplementary file 12 — Figure EV3 Source Data [file 44319_2024_82_MOESM12_ESM.zip › Figure EV3/EV3E/Ctl/Merged bottom.tif]

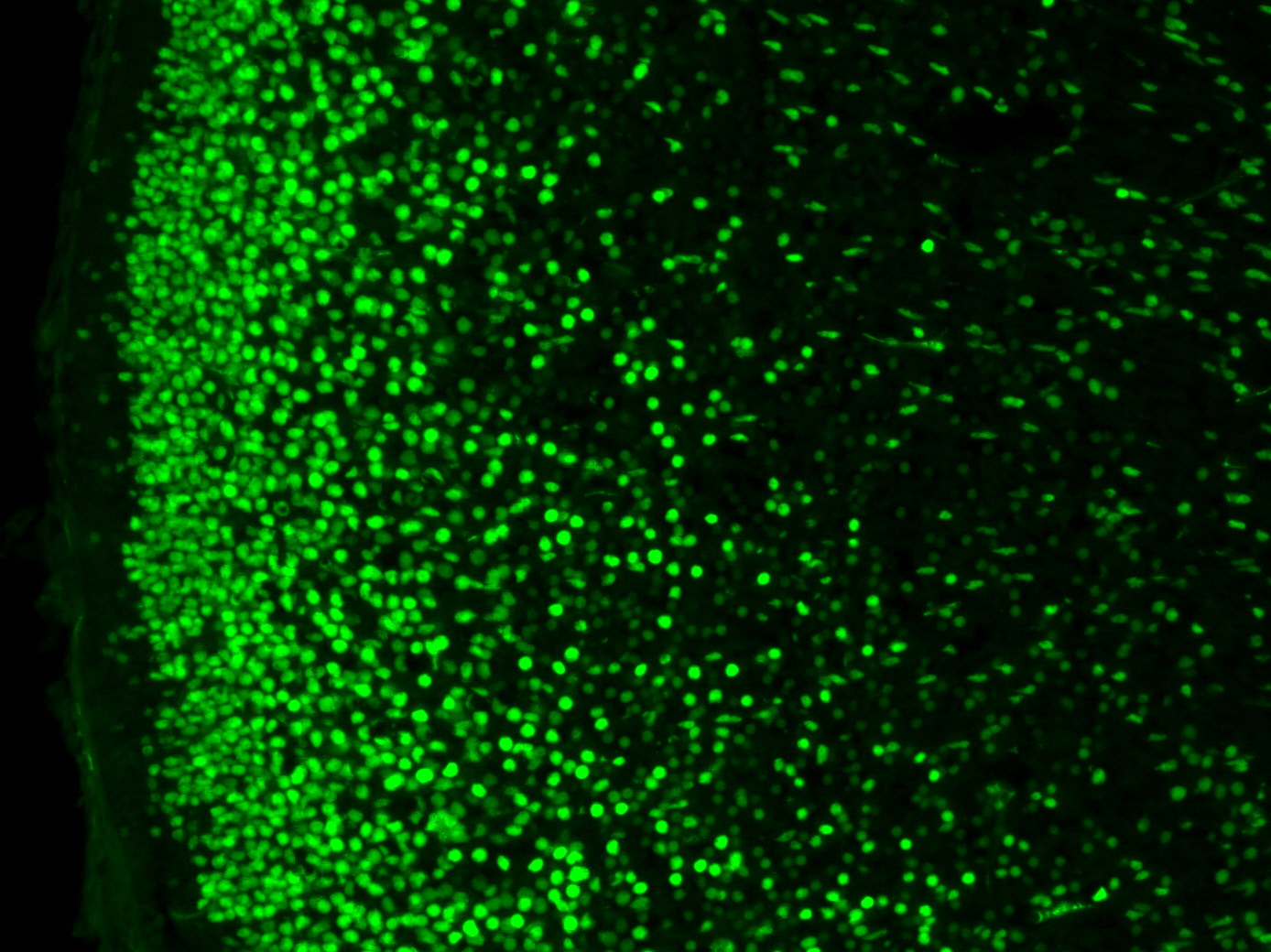

Supplement: Supplementary file 12 — Figure EV3 Source Data [file 44319_2024_82_MOESM12_ESM.zip › Figure EV3/EV3E/Ctl/Satb2 top.JPG]

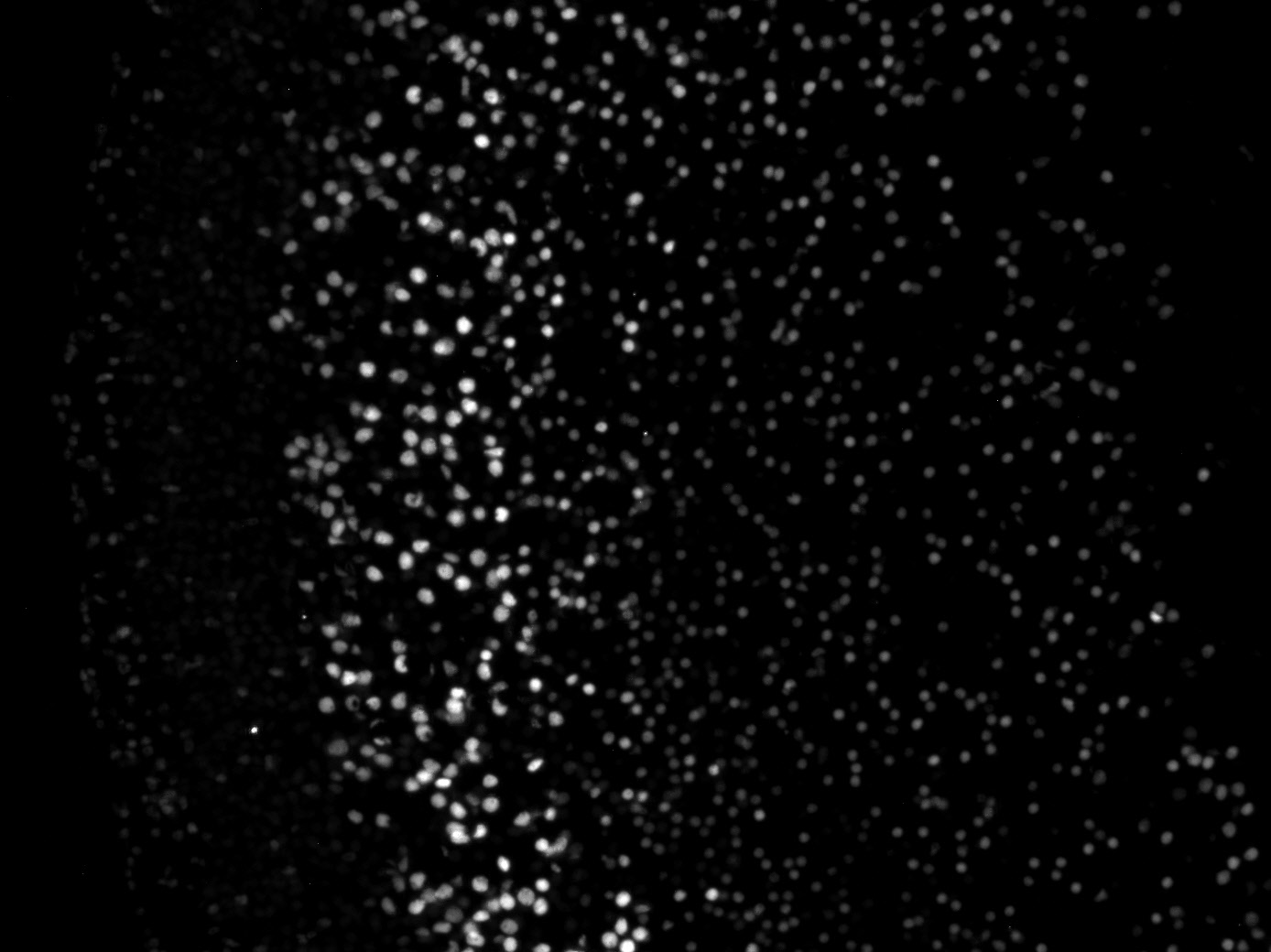

Supplement: Supplementary file 12 — Figure EV3 Source Data [file 44319_2024_82_MOESM12_ESM.zip › Figure EV3/EV3E/Ctl/CTIP2 top.JPG]

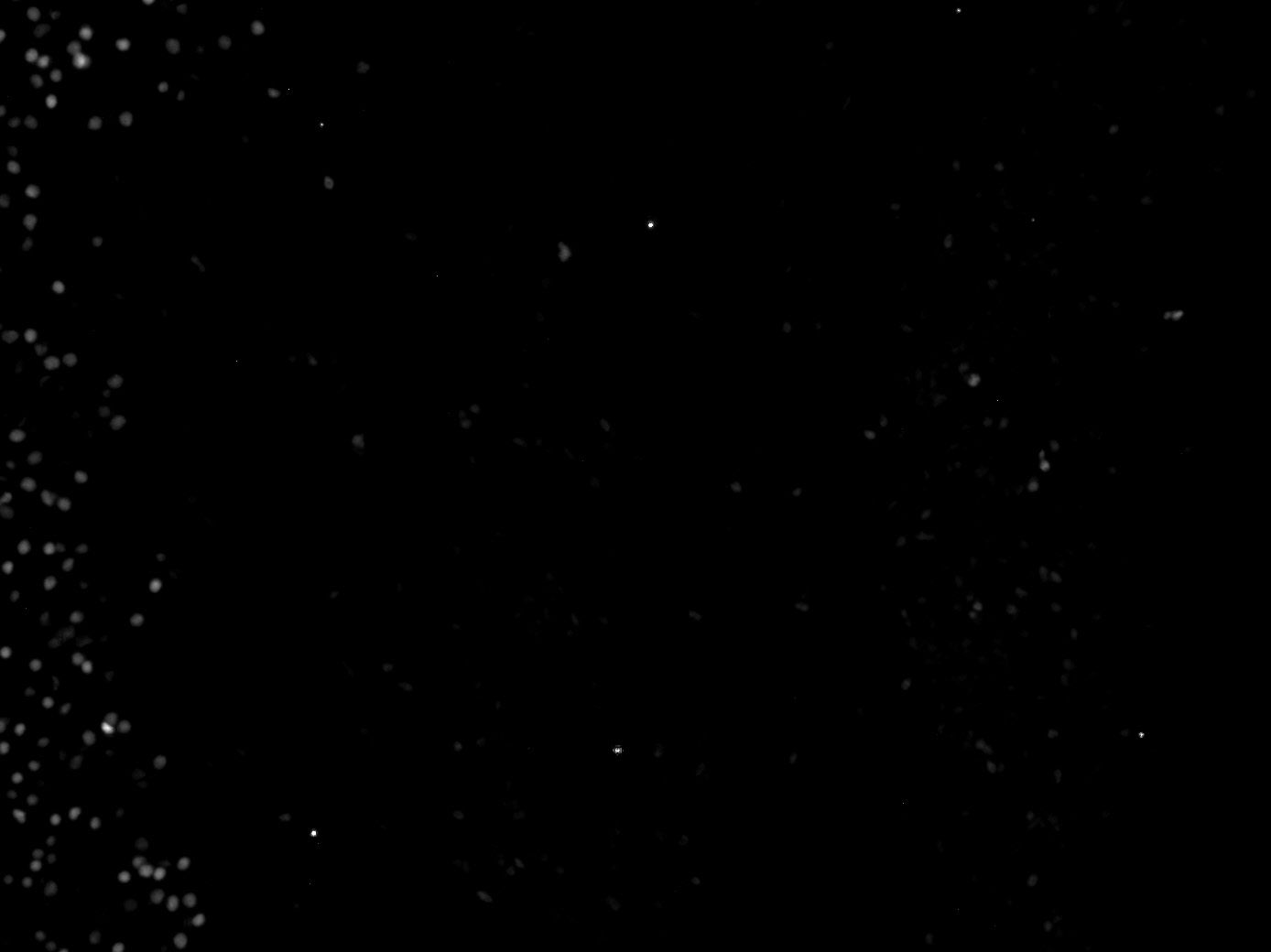

Supplement: Supplementary file 12 — Figure EV3 Source Data [file 44319_2024_82_MOESM12_ESM.zip › Figure EV3/EV3E/Ctl/CTIP2 bottom.JPG]

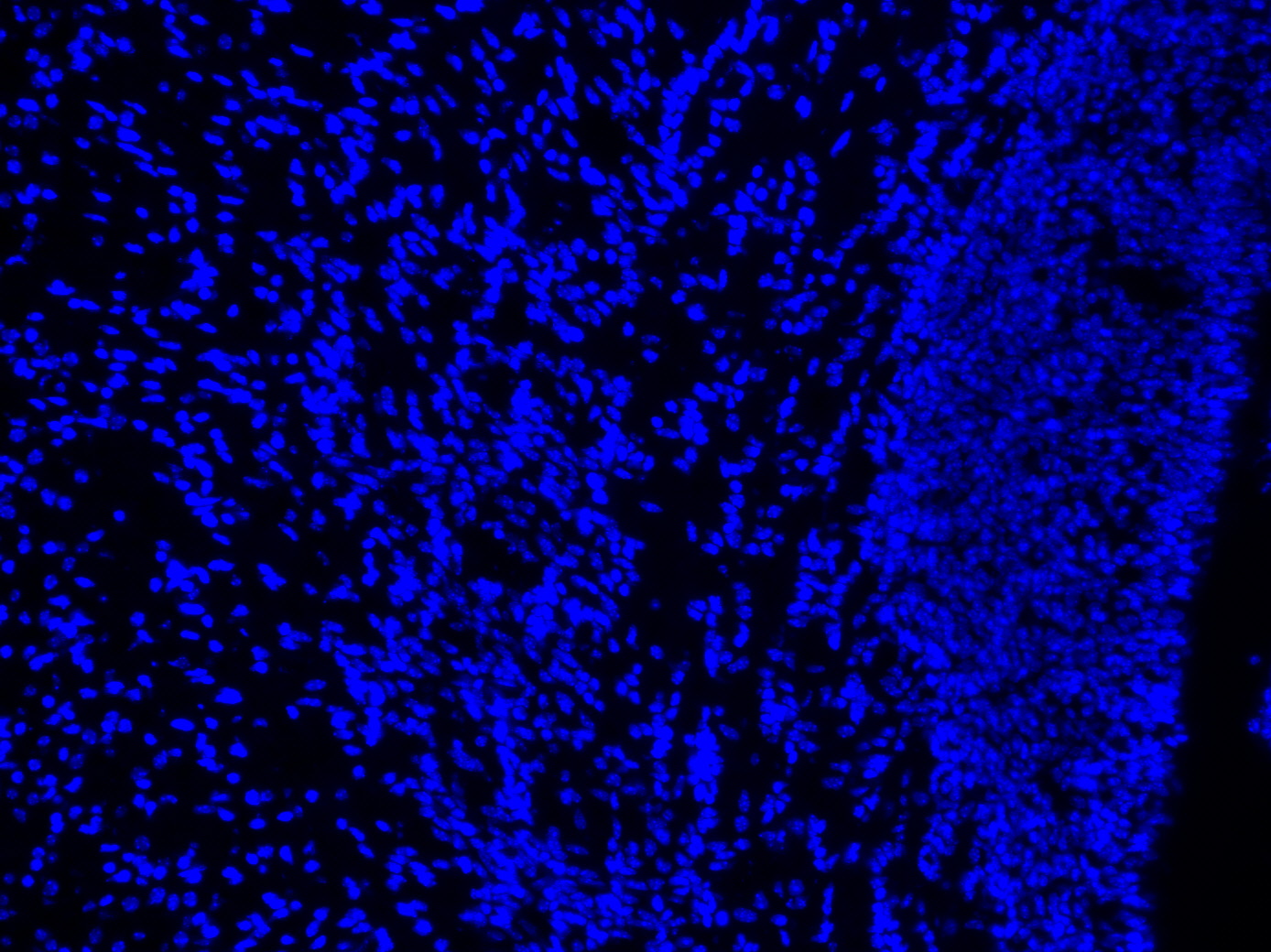

Supplement: Supplementary file 12 — Figure EV3 Source Data [file 44319_2024_82_MOESM12_ESM.zip › Figure EV3/EV3E/Ctl/Hoechst bottom.JPG]

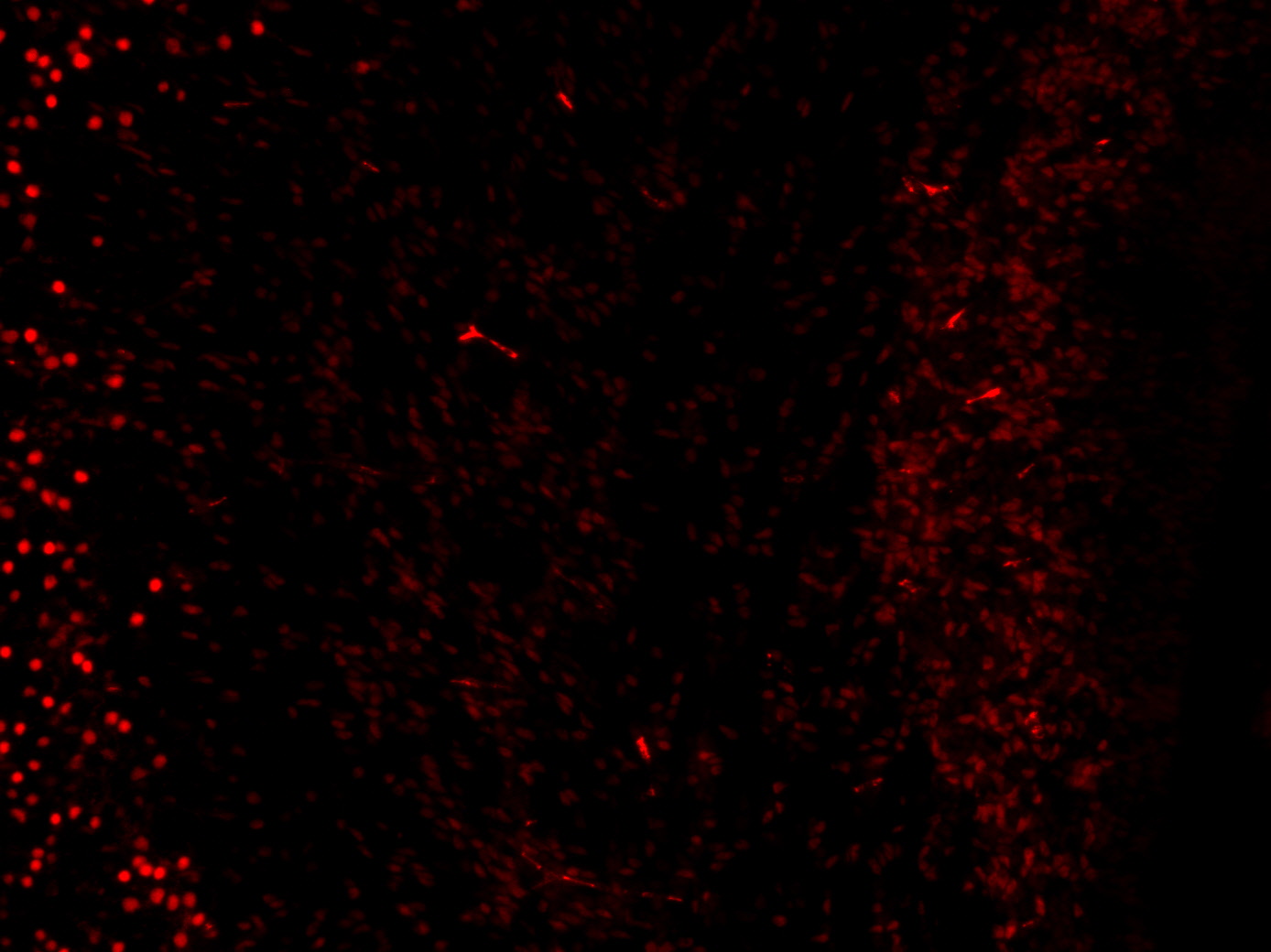

Supplement: Supplementary file 12 — Figure EV3 Source Data [file 44319_2024_82_MOESM12_ESM.zip › Figure EV3/EV3E/Ctl/Tbr1 2.JPG]

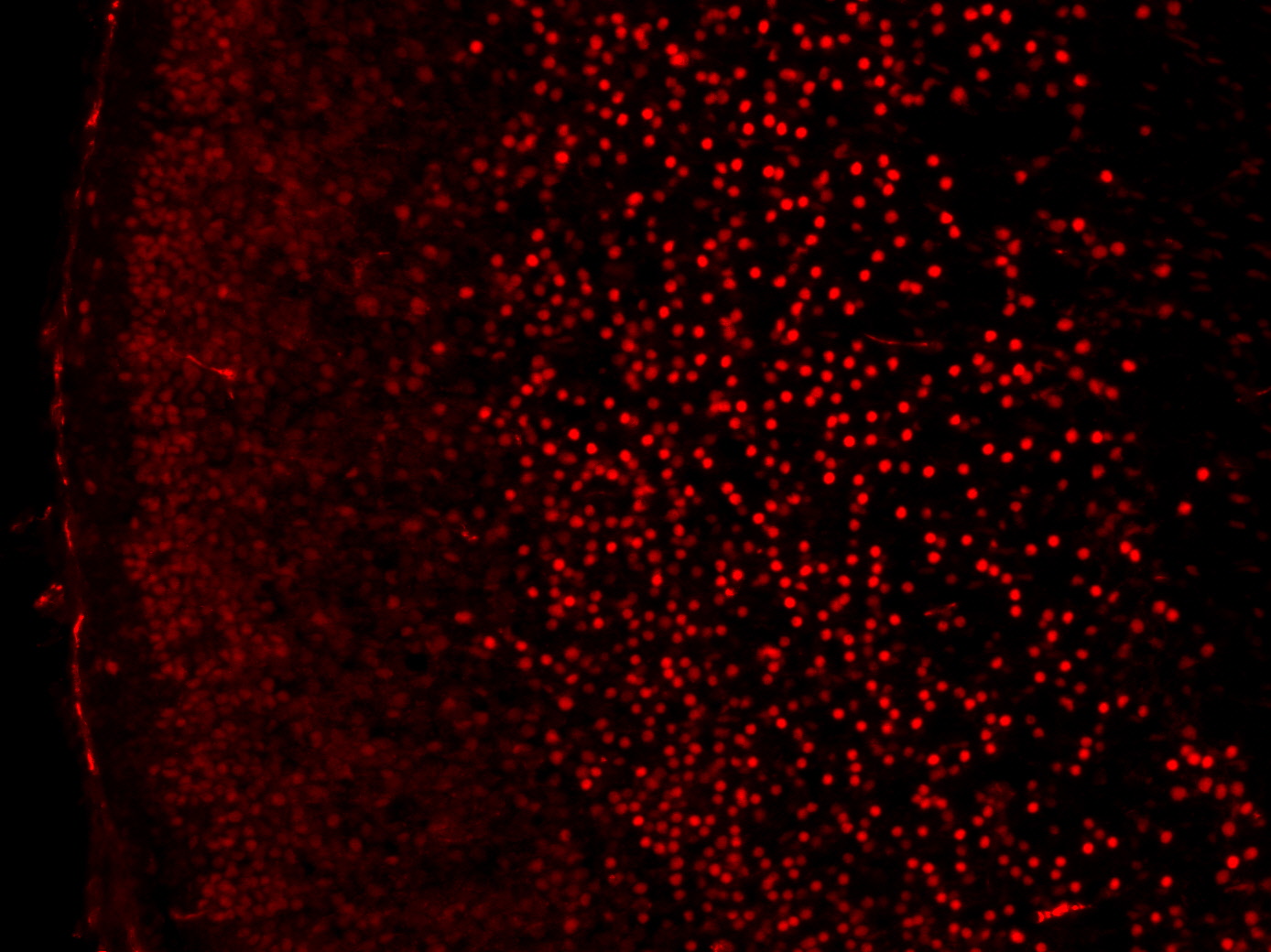

Supplement: Supplementary file 12 — Figure EV3 Source Data [file 44319_2024_82_MOESM12_ESM.zip › Figure EV3/EV3E/Ctl/Tbr1 1.JPG]
