# Supplementary material for: PHF6-mediated transcriptional control of NSC via Ephrin receptors is impaired in the intellectual disability syndrome BFLS
Source: EMBO Rep. 2024 Mar 1;25(3):20. doi: 10.1038/s44319-024-00082-0 (PMC10933485; doi:10.1038/s44319-024-00082-0)
Supplement: Supplementary file 14 — Expanded View Figures [file 44319_2024_82_MOESM14_ESM.pdf]

Expanded View Figures

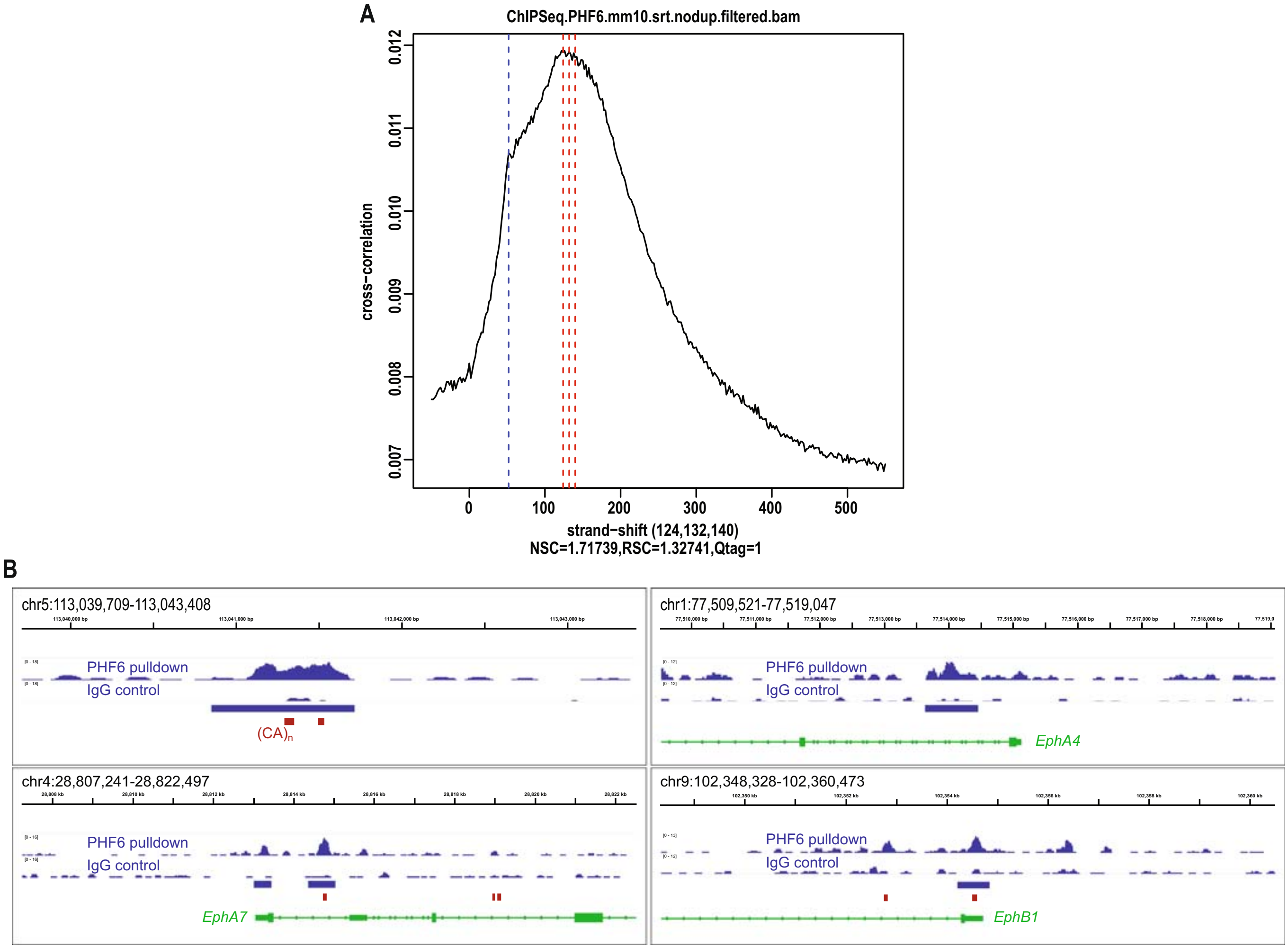

Figure EV1. PHF6 ChIP-Seq analysis.

(**A**) PHF6 ChIP-seq cross-correlation analysis was conducted using cross-correlation metrics as described in Landt et al, (Landt et al, 2012). (**B**) Example ChIP-seq tracks for PHF6 pull-down and IgG control. (CA)n repeats are demarcated with red boxes, while the blue boxes represent the identified PHF6 peak.

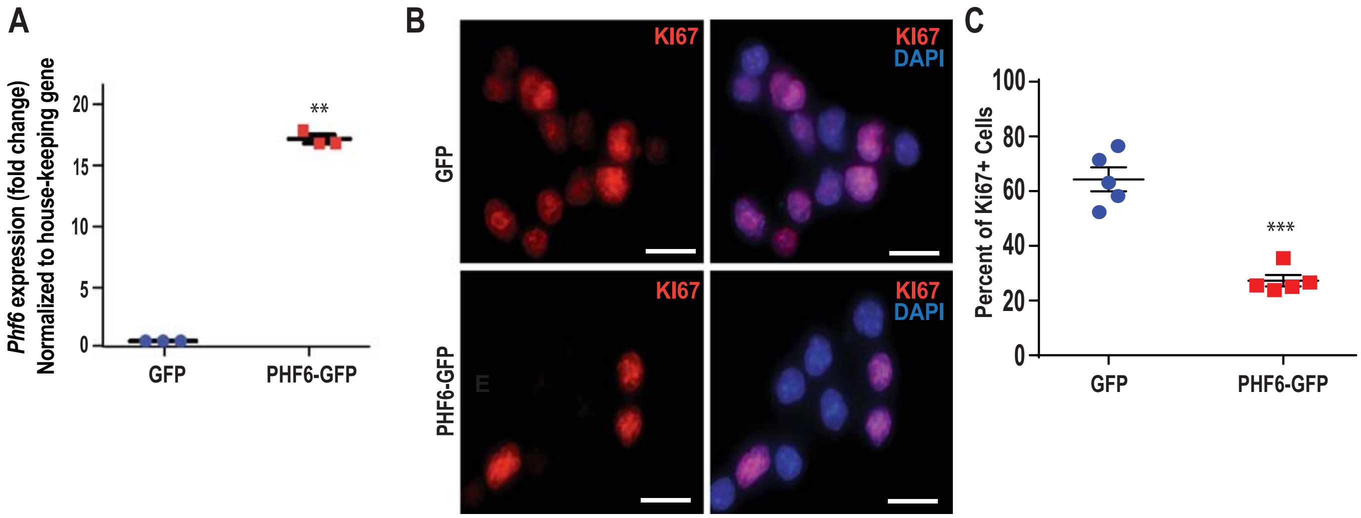

Figure EV2. PHF6 regulation of proliferation in neuroblastoma (N2A) cells.

(**A–C**) N2A cells were transfected with *Phf6* (PHF6-GFP) or GFP-expressing control (GFP) constructs. (**A**) Gene expression was assessed by RT-qPCR ($n = 3$). (**B**) Samples were subjected to KI67 staining for assessment of proliferation ($n > 3$, representative image shown). Scale bar represents 20 µm. (**C**) Quantification of percent KI67 positive cells are shown ($n > 3$). Data information: Data are presented as mean ± SEM. *$p < 0.05$, **$p < 0.01$, ***$p < 0.001$ (two-tailed unpaired student *t*-test). *n* represents an independent biological sample.

 © The Author(s)

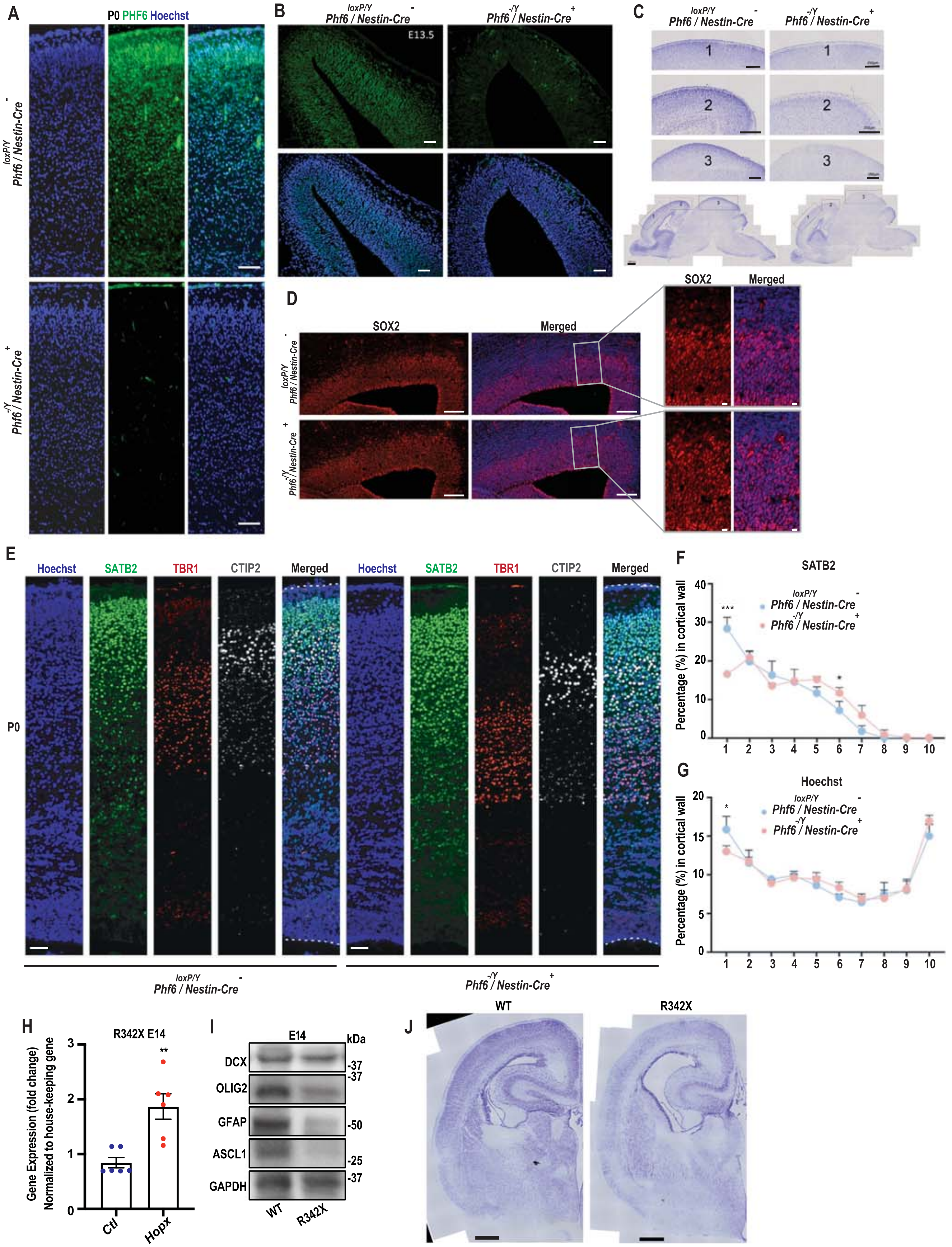

A
P0 PHF6 Hoechst
Phf6 loxP/Y / Nestin-Cre -
Phf6 -/Y / Nestin-Cre +
B
Phf6 loxP/Y / Nestin-Cre -
Phf6 -/Y / Nestin-Cre +
E13.5
C
Phf6 loxP/Y / Nestin-Cre -
Phf6 -/Y / Nestin-Cre +
1
2
3
D
SOX2
Merged
E
Hoechst
SATB2
TBR1
CTIP2
Merged
P0
F
SATB2
Percentage (%) in cortical wall
***
*
G
Hoechst
H
R342X E14
Gene Expression (fold change) Normalized to house-keeping gene
**
Ctl
Hopx
I
E14
kDa
DCX
OLIG2
GFAP
ASCL1
GAPDH
-37
-37
-50
-25
-37
WT
R342X
J
WT
R342X

Figure EV3. Characterization of *Phf6/Nestin-Cre* and BFLS mouse brain development.

(**A**,**B**) Immunofluorescence (IF) staining of coronal sections from P0 (**A**) and E13.5 (**B**) for *Phf6*$^{-/Y}$ / *Nestin-Cre*$^{+}$ and *Phf6*$^{loxp/Y}$ / *Nestin-Cre*$^{-}$ male mice using a PHF6 antibody (green) in the cerebral cortex. Nuclei were counterstained by Hoechst. Scale bars represent 50 μm. (**C**) *Phf6*$^{-/Y}$ / *Nestin-Cre*$^{+}$ and *Phf6*$^{loxp/Y}$ / *Nestin-Cre*$^{-}$ male mice were collected at P0 and subjected to Nissl staining with sagittal sections shown. Scale bars represent 500 μm in lower magnification and 250 μm in higher magnification photomicrographs. (**D**) IF staining of coronal sections from ~E15 male mice using a SOX2 antibody is shown. Scale bar represents 100 μm at lower magnification and 10 μm at higher magnification. (**E**) IF staining of coronal sections from P0 using cortical layer markers: SATB2 (green, layer II-V), TBR1 (red, layer VI), and CTIP2 (grey, layer V). Nuclei were counterstained by Hoechst. The cortical wall spanning from the basal of ventricle zone to the pial surface was equally divided into ten bins, the bin 1 covers the most superficial layer and bin 10 covers the deepest layer. (**F**) Comparative analysis of SATB2+ neurons in each segment of P0 male mice ($n = 3$). (**G**) Comparative analysis of Hoechst+ nuclei in each segment of P0 male mice (n=3). Scale bars represent 50 μm. (**H**,**I**) mRNA and protein of E14 R342X and wild-type control mice were subjected to RT-qPCR for *Hopx* expression ($n > 3$) (**H**) ($p = 0.0021$), and immunoblotting analysis of cell type-specific markers (**I**) ($n = 3$, representative blots shown). (**J**) R342X and WT mice were collected at P0 and subjected to Nissl staining ($n = 2$, representative image shown). Coronal sections are shown. Scale bars represent 500 μm. Data information: Data are presented as mean ± SEM. *$p < 0.05$, **$p < 0.01$, ***$p < 0.001$. two-tailed unpaired student *t*-test (**H**). two-way ANOVA with multiple comparisons (**F**,**G**). *n* represents an independent biological sample. Source data are available online for this figure.

 © The Author(s)

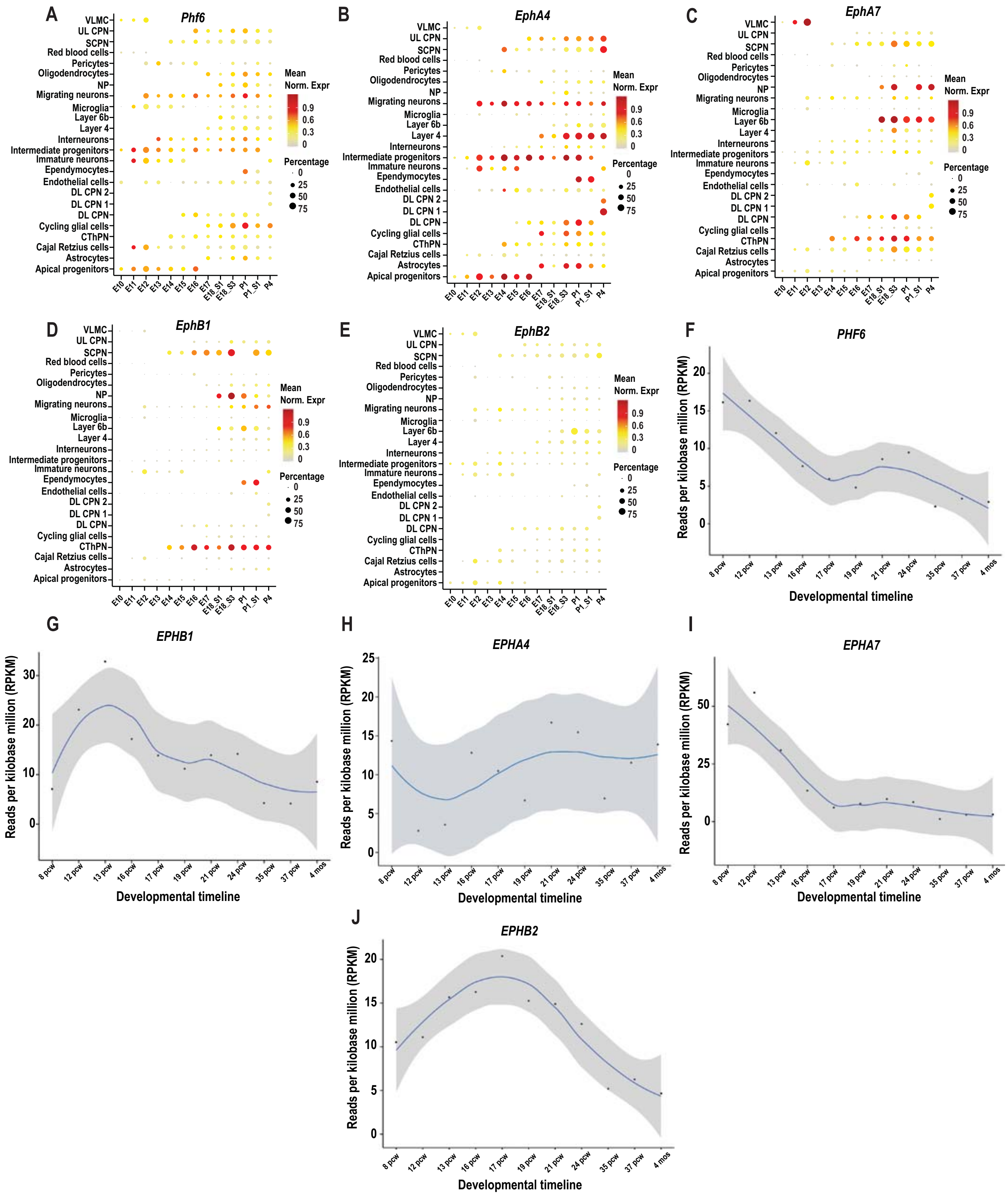
A
Phf6
B
EphA4
C
EphA7
D
EphB1
E
EphB2
VLMC
UL CPN
SCPN
Red blood cells
Pericytes
Oligodendrocytes
NP
Migrating neurons
Microglia
Layer 6b
Layer 4
Interneurons
Intermediate progenitors
Immature neurons
Ependymocytes
Endothelial cells
DL CPN 2
DL CPN 1
DL CPN
Cycling glial cells
CThPN
Cajal Retzius cells
Astrocytes
Apical progenitors
E10 E11 E12 E13 E14 E15 E16 E17 E18_S1 E18_S3 P1 P1_S1 P4
Mean
Norm. Expr
0.9
0.6
0.3
0
Percentage
0
25
50
75
F
PHF6
G
EPHB1
H
EPHA4
I
EPHA7
J
EPHB2
Reads per kilobase million (RPKM)
Developmental timeline
8 pcw 12 pcw 13 pcw 16 pcw 17 pcw 19 pcw 21 pcw 24 pcw 35 pcw 37 pcw 4 mos

Figure EV4. Analysis of *Phf6* and *EphR* mRNA expression across development.

(**A–E**) Dot plots showing expression of *Phf6* (**A**), *EphA4* (**B**), *EphA7* (**C**), *EphB1* (**D**), and *EphB2* (**E**) in the mouse cerebral cortex during development where the colour of each dot represents the mean normalized expression values per cell type for a given timepoint. The size of the circle represents the percentage of cells expressing each gene. Single cell mouse RNA-seq data was obtained from GEO GSE153164 [Data ref: (Di Bella et al, 2021)]. (**F–J**) Analysis of *PHF6* and *EPHR* expression in the human cortex. Average reads per kilobase million (RPKM) values over human developmental time (post-conceptual weeks; pcw) for gene analysis of *PHF6* (**F**), *EPHB1* (**G**), *EPHA4* (**H**), *EPHA7* (**I**), and *EPHB2* (**J**) are shown. Gene analysis was taken from publicly available RNA-seq data taken from the human ventral frontal cortex (VFC) of the Allen Brain Atlas BrainSpan dataset [Data ref: (BrainSpan Atlas of the Developing Human Brain, 2011)].

 © The Author(s)

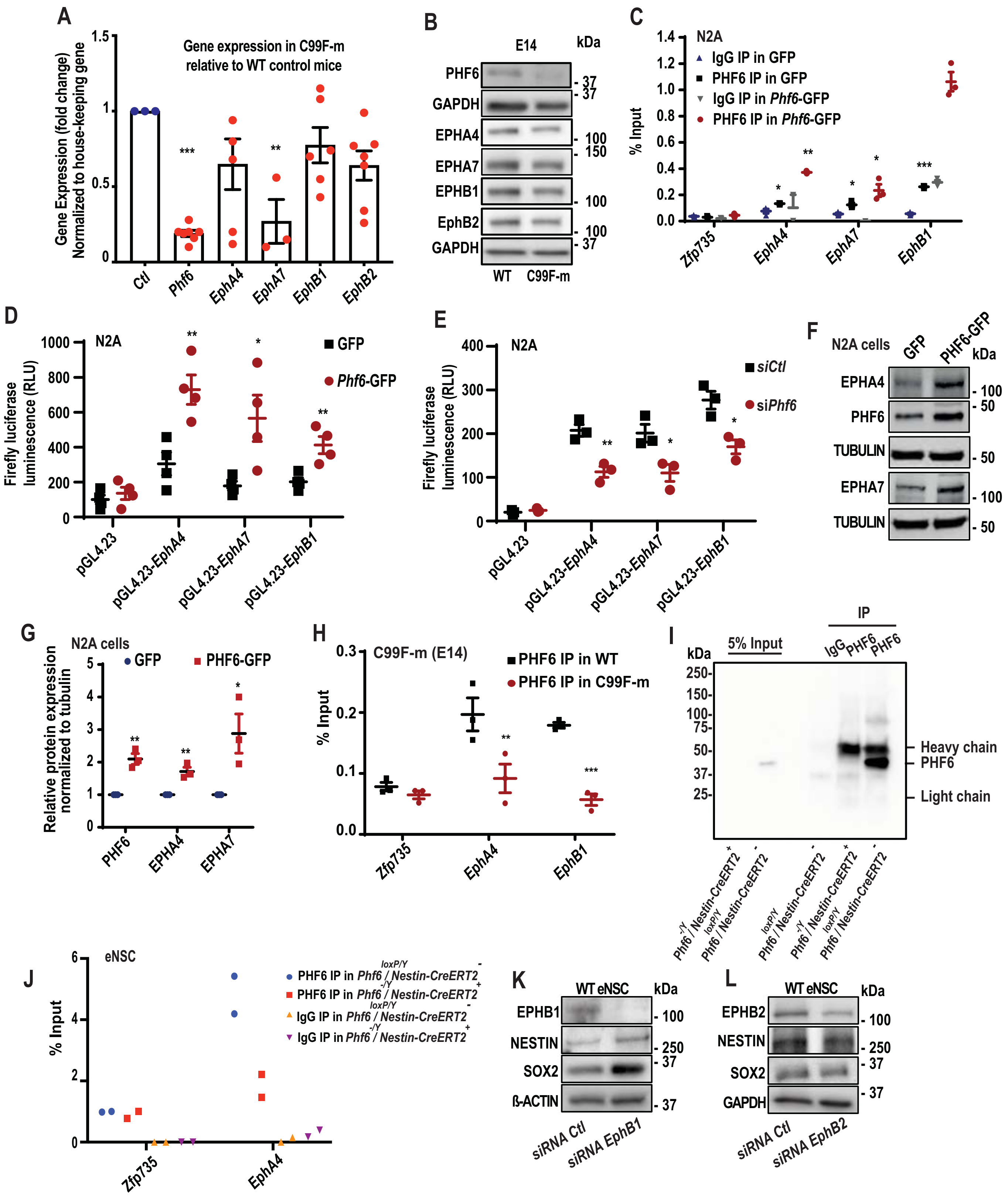

A
Gene expression in C99F-m relative to WT control mice
Gene Expression (fold change) Normalized to house-keeping gene
Ctl
Phf6
EphA4
EphA7
EphB1
EphB2
B
E14
kDa
PHF6
GAPDH
EPHA4
EPHA7
EPHB1
EphB2
GAPDH
WT
C99F-m
C
N2A
IgG IP in GFP
PHF6 IP in GFP
IgG IP in Phf6-GFP
PHF6 IP in Phf6-GFP
% Input
Zfp735
EphA4
EphA7
EphB1
D
N2A
GFP
Phf6-GFP
Firefly luciferase luminescence (RLU)
pGL4.23
pGL4.23-EphA4
pGL4.23-EphA7
pGL4.23-EphB1
E
N2A
siCtl
siPhf6
Firefly luciferase luminescence (RLU)
F
N2A cells
GFP
PHF6-GFP
kDa
EPHA4
PHF6
TUBULIN
EPHA7
TUBULIN
G
N2A cells
GFP
PHF6-GFP
Relative protein expression normalized to tubulin
PHF6
EPHA4
EPHA7
H
C99F-m (E14)
PHF6 IP in WT
PHF6 IP in C99F-m
% Input
Zfp735
EphA4
EphB1
I
kDa
5% Input
IP
IgG
PHF6
PHF6
Heavy chain
PHF6
Light chain
J
eNSC
% Input
Zfp735
EphA4
K
WT eNSC
kDa
EPHB1
NESTIN
SOX2
ß-ACTIN
siRNA Ctl
siRNA EphB1
L
WT eNSC
kDa
EPHB2
NESTIN
SOX2
GAPDH
siRNA Ctl
siRNA EphB2

Figure EV5. Analysis of PHF6 and EphR expression in BFLS mice.

(**A**,**B**) mRNA and protein of E14 C99F-m and wild-type control mice were subjected to RT-qPCR and immunoblotting analysis ($n \geq 3$). (**C**) GFP or PHF6-GFP expressing N2A cells were subjected to ChIP using an antibody to PHF6 or IgG control followed by PCR analysis using primers to *EphA4*, *EphA7* and *EphB1*. *Zfp* locus was used as control ($n = 3$). (**D**) GFP or PHF6-GFP- expressing cells were electroplated with a luciferase reporter plasmid driven by a promoter containing 583 bp of the *EphA4* gene (pGL4.23-*EphA4*), 550 bp of the *EphA7* gene (pGL4.23-*EphA7*) or 709 bp of the *EphB1* gene (pGL4.23-*EphB1*). The pGL4.23-basic reporter plasmid (pGL4.23) was used as a control. Renilla expression plasmid was used as an internal control for all samples. RLU Relative luminescence unit. Dual luciferase reporter assay was performed 48 h following electroporation ($n = 3$). (**E**) N2A cells were electroporated with siRNA against *Phf6* (si*Phf6*) or control siRNA (siCtl) followed by dual luciferase reporter assay at 48 h ($n = 3$). (**F**) EPHA4, EPHA7 and PHF6 levels were analyzed by immunoblotting in PHF6-GFP- expressing N2A cells. TUBULIN was used as a loading control. (**G**) Densitometric quantification of PHF6, EPHA4 and EPHA7 protein level normalized to TUBULIN is shown ($n = 3$). (**H**) E14-Cerebral cortical tissues from WT and C99F-m mice were subjected to ChIP-PCR analysis, as described in panel (**C**). (**I**) eNSCs cultured from *Phf6*$^{-/Y}$ / *Nestin-CreERT2*$^{+}$ and control *Phf6*$^{loxP/Y}$ / *Nestin-CreERT2*$^{-}$ ~E15 mouse brains were subjected to immunoprecipitation (IP) using PHF6 antibody or IgG as control followed by immunoblotting analysis using a PHF6 antibody. (**J**) eNSCs from *Phf6*$^{-/Y}$ / *Nestin-CreERT2*$^{+}$ and control *Phf6*$^{loxP/Y}$ / *Nestin-CreERT2*$^{-}$ mouse brains at ~E15, were subjected to ChIP-PCR using a PHF6 antibody. *Zfp735* loci was used as control for the PCR ($n = 2$). (**K**,**L**) Protein expression of EPHB1 (**K**), EPHB2 (**L**), SOX2 and NESTIN were analyzed by immunoblotting in *EphB1* and *EphB2* knockdown (KD) cells. Loading controls of ß-ACTIN and GAPDH were used ($n = 2$). Data information: Data are presented as mean ± SEM. *$p < 0.05$, **$p < 0.01$, ***$p < 0.001$. [(**C**,**H**) one-way ANOVA, (**A**,**D**,**E**,**G**) two-tailed unpaired student t-test]. n represents an independent biological sample.

 © The Author(s)
